# Gene–environment correlations and causal effects of childhood maltreatment on physical and mental health: a genetically informed approach

**DOI:** 10.1016/S2215-0366(20)30569-1

**Published:** 2021-05

**Authors:** Varun Warrier, Alex S F Kwong, Mannan Luo, Shareefa Dalvie, Jazz Croft, Hannah M Sallis, Jessie Baldwin, Marcus R Munafò, Caroline M Nievergelt, Andrew J Grant, Stephen Burgess, Tyler M Moore, Ran Barzilay, Andrew McIntosh, Marinus H van IJzendoorn, Charlotte A M Cecil

**Affiliations:** a Department of Psychiatry, University of Cambridge, Cambridge, UK; b MRC Biostatistics Unit, University of Cambridge, Cambridge, UK; c Department of Public Health and Primary Care, University of Cambridge, Cambridge, UK; d MRC Integrative Epidemiology Unit at the University of Bristol, University of Bristol, Bristol, UK; e Department of Population Health Sciences, Bristol Medical School, University of Bristol, Bristol, UK; f School of Psychological Science, University of Bristol, Bristol, UK; g Division of Psychiatry, Centre for Clinical Brain Sciences, University of Edinburgh, Edinburgh, UK; h Department of Psychology, Education and Child Studies, Erasmus University Rotterdam, Rotterdam, Netherlands; i Generation R Study Group, Erasmus MC, University Medical Center Rotterdam, Rotterdam, Netherlands; j South Africa MRC Unit on Risk and Resilience in Mental Disorders, Department of Psychiatry and Neuroscience Institute, University of Cape Town, Cape Town, South Africa; k Department of Clinical, Educational and Health Psychology, Division of Psychology and Language Sciences, University College London, London, UK; l Social, Genetic and Developmental Psychiatry Centre, Institute of Psychiatry, Psychology and Neuroscience, King's College London, London, UK; m NIHR Biomedical Research Centre at the University Hospitals Bristol NHS Foundation Trust and the University of Bristol, Bristol, UK; n Department of Psychiatry, University of California San Diego, La Jolla, CA, USA; o Center of Excellence for Stress and Mental Health, Veterans Affairs San Diego Healthcare System, San Diego, CA, USA; p Research Service, Veterans Affairs San Diego Healthcare System, San Diego, CA, USA; q Veterans Affairs San Diego Healthcare System, San Diego, CA, USA; r Department of Psychiatry, University of Pennsylvania, Philadelphia, PA, USA; s Lifespan Brain Institute of the Children's Hospital of Philadelphia and University of Pennsylvania, Philadelphia, PA, USA; t Division of Psychiatry, Centre for Clinical Brain Sciences, University of Edinburgh, Edinburgh, UK; u Centre for Cognitive Ageing and Cognitive Epidemiology, School of Philosophy, Psychology and Language Sciences, University of Edinburgh, Edinburgh, UK; v Department of Child and Adolescent Psychiatry, Erasmus MC, Rotterdam, Netherlands; w Department of Epidemiology, Erasmus MC, Rotterdam, Netherlands; x Molecular Epidemiology, Department of Biomedical Data Sciences, Leiden University Medical Center, Leiden, Netherlands

## Abstract

**Background:**

Childhood maltreatment is associated with poor mental and physical health. However, the mechanisms of gene–environment correlations and the potential causal effects of childhood maltreatment on health are unknown. Using genetics, we aimed to delineate the sources of gene–environment correlation for childhood maltreatment and the causal relationship between childhood maltreatment and health.

**Methods:**

We did a genome-wide association study meta-analysis of childhood maltreatment using data from the UK Biobank (n=143 473), Psychiatric Genomics Consortium (n=26 290), Avon Longitudinal Study of Parents and Children (n=8346), Adolescent Brain Cognitive Development Study (n=5400), and Generation R (n=1905). We included individuals who had phenotypic and genetic data available. We investigated single nucleotide polymorphism heritability and genetic correlations among different subtypes, operationalisations, and reports of childhood maltreatment. Family-based and population-based polygenic score analyses were done to elucidate gene–environment correlation mechanisms. We used genetic correlation and Mendelian randomisation analyses to identify shared genetics and test causal relationships between childhood maltreatment and mental and physical health conditions.

**Findings:**

Our meta-analysis of genome-wide association studies (N=185 414) identified 14 independent loci associated with childhood maltreatment (13 novel). We identified high genetic overlap (genetic correlations 0·24–1·00) among different maltreatment operationalisations, subtypes, and reporting methods. Within-family analyses provided some support for active and reactive gene–environment correlation but did not show the absence of passive gene–environment correlation. Robust Mendelian randomisation suggested a potential causal role of childhood maltreatment in depression (unidirectional), as well as both schizophrenia and ADHD (bidirectional), but not in physical health conditions (coronary artery disease, type 2 diabetes) or inflammation (C-reactive protein concentration).

**Interpretation:**

Childhood maltreatment has a heritable component, with substantial genetic correlations among different operationalisations, subtypes, and retrospective and prospective reports of childhood maltreatment. Family-based analyses point to a role of active and reactive gene–environment correlation, with equivocal support for passive correlation. Mendelian randomisation supports a (primarily bidirectional) causal role of childhood maltreatment on mental health, but not on physical health conditions. Our study identifies research avenues to inform the prevention of childhood maltreatment and its long-term effects.

**Funding:**

Wellcome Trust, UK Medical Research Council, Horizon 2020, National Institute of Mental Health, and National Institute for Health Research Biomedical Research Centre.

## Introduction

Childhood maltreatment, including abuse and neglect, is a complex global problem, affecting up to 36% of the population worldwide.^1^, ^2^ According to most definitions, childhood maltreatment is inflicted by an agent who can be held responsible for the wellbeing of the child, with up to 80% of perpetrators being the parents or other family members of the child.^2^, ^3^

Parental experience of childhood maltreatment is an important risk factor for maltreating their own children.^4^, ^5^ This intergenerational transmission of childhood maltreatment might partly be due to shared social factors or to shared genetics between parents and their offspring. Studies that quantify the variance in childhood maltreatment attributable to genetics (broad-sense heritability), show childhood maltreatment has a twin and familial heritability of 6–62%.^6^, ^7^, ^8^ It is also possible to investigate heritability attributable to the additive effects of single nucleotide polymorphisms (SNPs), and a study from 2020 has shown a SNP heritability of 6% for childhood maltreatment.^9^

The heritable component of childhood maltreatment is not immutable and is thought to manifest through gene–environment correlations: passive (parental genes influence family environments and are inherited by their children), active (children's genes shape their behaviours including risk-taking behaviour), and reactive or evocative (in which children's genes shape their behaviour and physical features, eliciting different responses from their parents or others).^6^, ^10^, ^11^, ^12^, ^13^ These mechanisms might vary among different groups of individuals. For example, children with ADHD and autism might be at an increased risk for childhood maltreatment compared to typically developing children,^14^, ^15^ owing to mismatch between parental expectation and child behaviour (eg, parental expectation of greater social responsiveness and lower activity levels), which is a form of reactive gene–environment correlation. Neither active or reactive gene–environment correlation indicates that the child is to blame, as the responsibility for protecting a child lies primarily with the parents and additionally with society at large. No study, to our knowledge, has delineated the proportion of variance in childhood maltreatment explained by different gene–environment correlation mechanisms, which could help guide family-based prevention strategies.

**Research in context**

**Evidence before this study**

Childhood maltreatment is known to be partly heritable but how different mechanisms of gene–environment correlation contribute to childhood maltreatment, and whether childhood maltreatment is causally related to mental and physical health outcomes is unknown. We searched PubMed and Google Scholar for genome-wide association studies and Mendelian randomisation analyses of childhood maltreatment from Jan 1, 1990, to Oct 1, 2020. Search terms were “childhood maltreatment” OR “childhood trauma” AND “GWAS” OR “genome-wide association study” OR “Mendelian Randomization”, restricting the search to articles published in English. We found one genome-wide association study of childhood maltreatment with two identified genetic loci. We did not identify any studies investigating mechanisms of gene–environment correlations or causal effects of childhood maltreatment using Mendelian randomisation.

**Added value of this study**

In this genome-wide meta-analysis (N=185 414) of childhood maltreatment—the largest to date to our knowledge—we identified 14 independent genetic variants (13 novel) associated with childhood maltreatment. Using within-family polygenic score analyses, we found that this genetic signal is due to multiple gene–environment correlation mechanisms. We found some direct evidence for active and reactive gene–environment correlation, but could not show an absence of passive gene–environment correlation. Finally, in this first Mendelian randomisation analyses of childhood maltreatment, to our knowledge, we identified potentially causal effects of childhood maltreatment on depression. We also identified bidirectional potentially causal effects between childhood maltreatment and both ADHD and schizophrenia. However, we found no evidence for causal effects of childhood maltreatment on physical health or inflammatory markers, or vice versa.

**Implications of all the available evidence**

Our study supports findings from observational epidemiological studies, providing the first evidence using Mendelian randomisation of potential causal relationships between childhood maltreatment and mental health conditions. These findings underscore the need to prevent childhood maltreatment and to identify mediating and moderating factors that can help minimise the effect of childhood maltreatment on mental health. However, in contrast to observational studies, we found no association between childhood maltreatment and physical health. Although childhood maltreatment is only partly heritable, delineating the source of the heritability is important as different gene–environment correlation mechanisms might indicate the need for different intervention strategies. Further research is needed to establish whether these gene–environment correlation mechanisms differ between populations and subtypes of childhood maltreatment.

Observational studies link childhood maltreatment with severe and long-lasting mental (eg, psychosis,^16^ depression,^17^ ADHD^14^) and physical health problems^2^, ^5^ (eg, diabetes,^18^ cardiovascular disease^19^). Yet, there is considerable heterogeneity in these observations, partly because studies differ in how childhood maltreatment is operationalised,^20^ whether they examine the effects of specific subtypes,^21^ and whether prospective or retrospective reports of childhood maltreatment are used, which overlap only modestly.^22^ Furthermore, it is unclear whether observational associations are due to causal effects of childhood maltreatment, or to genetic or environmental confounding (eg, deprivation).^2^, ^5^, ^23^ For example, poor parental mental health is a risk factor for childhood maltreatment. Propensity for mental illness in children who are maltreated might be inherited from parents independently of having experienced childhood maltreatment (genetic confounding). Similarly, social deprivation is a risk for maltreatment and might also be independently associated with adverse health outcomes, thus presenting incorrectly as an association between adverse health and childhood maltreatment (environmental confounding). Identifying causal effects could help to mitigate adverse sequalae of childhood maltreatment.

Some studies have sought to investigate causality using co-twin control methods.^24^, ^25^ However, ascribing causality in these studies is difficult, as twin studies rely on certain assumptions (eg, equal environment assumptions) and cannot exclude reverse causation, especially if longitudinal data are unavailable. It might be challenging to control for non-shared environments, particularly those that influence later health outcomes (eg, lifestyle choices and coronary artery disease). Mendelian randomisation^26^ assumes that a genetic variant associated with an exposure can be used as an unconfounded proxy for that exposure to investigate its effect on potential outcomes. For example, a Mendelian randomisation study found a protective effect of exercise on major depressive disorder.^27^ No study, to our knowledge, has used Mendelian randomisation to investigate the causal mechanisms of childhood maltreatment on mental and physical health.

Here, we present the largest genome-wide association study (GWAS) meta-analysis of childhood maltreatment to date, to our knowledge, to investigate the underlying mechanisms of childhood maltreatment, focusing on offspring DNA. We aimed to quantify the SNP heritability and genetic correlations across different operationalisations, subtypes, and reporting methods of childhood maltreatment. Finally, we investigated the genetic correlations and causal effects between childhood maltreatment and outcomes related to mental and physical health.

## Methods

### Overview

In this GWAS meta-analysis, we had access to individual-level data from 159 124 participants from four datasets: 143 473 participants from the UK Biobank (birth year 1936–70)^28^; 5400 from the Adolescent Brain Cognitive Development Study (birth year 2006–08)^29^; 8346 from the Avon Longitudinal Study of Parents and Children (ALSPAC; birth year 1991–92);^30^, ^31^ and 1905 from the Generation R Study (birth year 2002–06).^32^, ^33^ In addition, we obtained summary GWAS statistics for the Psychiatric Genomics Consortium (PGC_26K) dataset (n=26 290 from 18 cohorts^9^). All participants (N=185 414) were primarily of European genetic ancestry and provided information on childhood maltreatment. Further details are shown in the appendix (pp 5–7, 61–63).

Ethical approval was obtained from the Human Biology Research Ethics Committee, University of Cambridge (Cambridge, UK), and from the local cohort ethics committees.

### Phenotypes

We define childhood maltreatment here as consisting of emotional, sexual, and physical abuse, and emotional and physical neglect. All retrospective reports of childhood maltreatment were self-reported, whereas most prospective reports of childhood maltreatment were reported by a parent or caregiver.

Participants in the UK Biobank completed the retrospectively reported five-item Childhood Trauma Screener.^34^ This assessment consists of one question for each of the five trauma subtypes, each ranging from 0 (never true) to 4 (very often true), with total scores ranging from 0–20. Participants in the PGC_26K completed different retrospective questionnaires on childhood maltreatment, which included questions only on sexual, physical, and emotional abuse. Total scores differed between the questionnaires, but all were coded continuously. In the Adolescent Brain Cognitive Development Study, parent-completed information on prospectively reported childhood maltreatment was used. This assessment comprised 13 questions on childhood maltreatment from the Kiddie Schedule for Affective Disorders and Schizophrenia-PTSD^35^ and the Children's Report of Parental Behavior Inventory,^36^ with scores ranging from 0–13. We used continuous scores that were rank-based inverse normal transformed for the analyses. In ALSPAC, childhood maltreatment was prospectively recorded using multiple questionnaires at multiple instances (majority parent-report, several self-report), detailed elsewhere.^16^ Scores were binarised with any instance of childhood maltreatment indicated as 1 and no report of childhood maltreatment indicated as 0, in line with previous analyses.^16^ In Generation R, childhood maltreatment was prospectively measured using mother-completed questionnaires including items on the Life Event and Difficulty Schedule,^37^ with scores ranging from 0–2, which were continuously coded. Further details are provided in the appendix (pp 7–10, 27, 61–63).

With the UK Biobank data, we investigated heritability of and genetic correlations among different subtypes and operationalisations of childhood maltreatment (appendix pp 7–10). For subtype analyses, we binarised the five phenotypes (sexual, emotional, and physical abuse, and emotional and physical neglect) due to skewness in the phenotypes. We defined four operationalisations of childhood maltreatment: a log-transformed sum-score of childhood maltreatment; a binary maltreatment score (0 *vs* any); a binary severe maltreatment score, in which scores 2–4 for each individual item were recorded as 1; and a binary severe childhood abuse score, which is the same as above but restricted to the three abuse items only.

### GWAS meta-analysis and functional annotation

For each GWAS, age and sex were included as covariates. The primary GWAS in the UK Biobank was done using the BOLT-LMM, version 2.3.4, algorithm,^38^ with batch included as an additional covariate. GWAS in ALSPAC was done using BOLT-LMM, version 2.3.4,^38^ GWAS in Adolescent Brain Cognitive Development Study was done using FastGWA (using GCTA version 1.93.2),^39^ and we used linear regression using Plink,^40^ version 2.0, for Generation R. The linear mixed-effects models used in the Adolescent Brain Cognitive Development Study, UK Biobank, and ALSPAC account for both population stratification and relatedness. However, we included genetic principal components (up to 20) to accelerate the model identification process.^38^ In Generation R, we included five genetic principal components as covariates to account for population stratification (appendix pp 10–13). We confirmed that there was no evidence of inflation in the test statistics of the GWAS by using the linkage disequilibrium score regression (LDSC)-based intercept.^41^

Sample-size weighted meta-analysis was done in METAL, version 2011-03-25.^42^ We did three meta-analyses. First, we meta-analysed the UK Biobank and the PGC_26K datasets to obtain a GWAS of retrospectively reported childhood maltreatment (GWAS_retrospective_). Next, we meta-analysed Adolescent Brain Cognitive Development Study, ALSPAC, and Generation R datasets to obtain a GWAS of prospectively reported childhood maltreatment (GWAS_prospective_). Finally, we meta-analysed all five datasets to obtain a GWAS of childhood maltreatment (GWAS_childhoodmaltreatment_).

Independent significant loci were identified at a GWAS threshold of p<5 × 10^–8^, after clumping (*r*^2^=0·1, 1000 kb), using the linkage disequilibrium weights generated from the European subset of the 1000 Genomes phase 3 dataset^43^ in Plink, version 1.9. Functional annotation of top loci was done using expression quantitative trait loci data from GTEx,^44^ BRAINEAC,^45^ CommonMind Consortium,^46^ and PsychEncode,^47^ Hi-C data, positional mapping, and associations with other health-related phenotypes all using FUMA.^48^

Gene identification was done using MAGMA,^49^ with the summary GWAS statistics. We identified significantly associated genes after Bonferroni correction. Enrichment for tissues and cell types was done using MAGMA and LDSC-specifically expressed genes (SEG),^50^ using summary GWAS statistics (appendix p 14). Specifically, using LDSC-SEG, we investigated enrichment for tissue-specific chromatin marks (ENCODE^51^ and Roadmap Epigenomics Project^52^) and gene expression (GTEx), and corrected each of these analyses for multiple testing using Benjamini-Hochberg false discovery rate correction to account for the correlated nature of the variables tested and the enrichment for gene expression using Bonferroni correction. Using MAGMA, we investigated enrichment for genes with tissue-type (GTEx^44^) and cell-type specific expression in neuronal cell types (PsychEncode^47^), and corrected each of these analyses for multiple testing using Bonferroni correction.

### SNP heritability and genetic correlations

SNP heritability of and genetic correlations between operationalisations and subtypes of childhood maltreatment were done using GCTA-GREML, version 1.93.2^53^ in a random subset of 19 559 unrelated individuals from the UK Biobank (grm-cutoff for relatedness=0·05). For all analyses, we included year of birth, sex, genotyping batch, and the first 20 genetic principal components as covariates. Results were corrected for multiple testing using Benjamini-Hochberg false discovery rate correction owing to the correlated nature of the phenotypes.

Heritability analyses of the meta-analysed GWAS and other genetic correlations were done using LDSC^54^ (appendix p 13), focusing on mental and physical health conditions, and psychological, behavioural, and anthropometric traits. We corrected for the 97 phenotypes tested using Bonferroni correction.

### Polygenic score analyses

We investigated the variance explained by polygenic score (PGS) in two cohorts—a hold-out sample from the UK Biobank and ALSPAC. PGSs were calculated using PRSice-2^55^ (clump *r*^2^=0·1, 250kb, appendix pp 15–19). Only autosomes were included in the calculation of PGS as there is no consensus for how to handle sex chromosomes in PGS analyses.^56^ To identify variance explained by the GWAS of childhood maltreatment, we did PGS analyses using GWAS_retrospective_ (base sample) in a hold-out sample of 12 855 individuals from the UK Biobank (target sample). This process was done by doing a second GWAS of childhood maltreatment in the UK Biobank excluding the hold-out sample (log-transformed sum-score, n=130 618) and meta-analysing the results with the PGC_26K. PGSs were generated in 9924 unrelated individuals from this subset of 12 855 individuals at 11 p value thresholds. We included birth year, sex, genotyping batch, and the first 20 genetic principal components as covariates, and additionally Townsend Deprivation Index in a second model. We corrected for multiple testing using Benjamini-Hochberg false discovery rate correction.

In ALSPAC (target sample), we regressed the PGS for GWAS_retrospective_ (base sample) against binarised childhood maltreatment (prospectively reported) at four age groups (0–17, 0–4·9, 5–10·9, 11–17 years), with age (in months), sex, and the first ten genetic principal components as ALSPAC (maximum N=7453). We used only ten principal components as ALSPAC is a geographically—and subsequently, genetically—more homogenous cohort than the UK Biobank, and previous research has found poor evidence for population stratification in ALSPAC.^57^ We restricted the PGS analyses in ALSPAC to PGS at a p value threshold of 1 as scores calculated at this threshold explained the highest variance in the hold-out sample from the UK Biobank. We corrected for the four different timepoints using Benjamini-Hochberg false discovery rate correction.

### Contribution of different mechanisms to childhood maltreatment

We used three methods to delineate the contribution of different gene–environment correlation mechanisms to childhood maltreatment: (1) comparing between-sibling and between-family effects; (2) polygenic transmission disequilibrium tests in two autism cohorts; and (3) investigating the variance explained by PGS for retrospective childhood maltreatment in ALSPAC after accounting for well known familial risk factors for childhood maltreatment.

To quantify the variance explained by passive gene–environment correlation and by active and reactive gene–environment correlation combined, we simultaneously investigated between-sibling and between-family effects of PGS (base sample GWAS_childhoodmaltreatment_)^58^, ^59^ in a hold-out sample of 12 855 individuals from the UK Biobank (including 2849 sibling pairs, target sample; appendix pp 15–19), using a mixed-effects regression model with the following equation:

$$Childhood\_maltreatment_{ij}∼\beta_{bsib}(PGS_{ij}-\bar{PGS_{j}})+\beta_{bfam}(\bar{PGS_{j}})+Z_{(1..n)\mathrm{cov}ariates}$$

where β_bsib_ is the between-sibling effect of PGS (representing reactive and active gene–environment correlation combined); β_bfam_ is the between-family effects; and (*PGS*_j_) is the family-mean PGS. Covariates included were age, sex, genotyping batch, and 20 genetic principal components. Passive gene–environment correlation is estimated from the difference between β_bsib_ and β_bfam_. SE was calculated by 10 000 bootstraps. In the UK Biobank, the family mean is the sibling mean.

Although between-sibling PGS analysis assumes that a proportion of the familial environment is shared between siblings, this assumption might not always be true. An example is siblings who are discordant for neurodevelopmental conditions such as autism or ADHD. Because of the different support needs of the siblings, parental response to the two siblings will be different. This difference in familial environment between siblings indexes reactive gene–environment correlation, and to an extent active gene–environment correlation. To quantify this difference in gene–environment correlation, we did polygenic transmission disequilibrium tests^60^ in two cohorts: Simons Simplex Collection^61^ (n=2234 autistic individuals and n=1829 non-autistic siblings) and SPARK^62^ (n=2957 autistic individuals and n=1567 non-autistic siblings; appendix p 19) to investigate over-transmission of PGS for childhood maltreatment to autistic individuals versus non-autistic siblings.^15^, ^60^ We use the term autistic as identity-first language is preferred by many autistic individuals.

Finally, we repeated the PGS analyses in ALSPAC, as outlined earlier, after including in separate models four parental risk factors^5^ of childhood maltreatment (ie, smoking, alcohol consumption, depression, and parental maltreatment). The risk factors were all measured prenatally to minimise the influence of a child's behaviour on parental phenotypes, leading to reactive gene–environment correlation (N=5988 to N=4508; see appendix p 70 for individual sample sizes). We corrected for multiple testing using Benjamini-Hochberg false discovery rate correction for each of the risk factors tested. Incomplete attenuation of the effects of PGS after accounting for known parental risk factors provided support for the active and reactive gene–environment correlation. We adjusted for eight phenotypes: parental depression assessed in the first trimester of pregnancy; parental depressive symptoms assessed in the second trimester; maternal depressive symptoms assessed in the third trimester and paternal depressive symptoms assessed in the second trimester; parental alcohol consumption assessed in the second trimester; parental alcohol consumption assessed in the third trimester; parental smoking assessed in the second trimester; parental smoking assessed in the third trimester; and parental history of childhood maltreatment, assessed across all trimesters.

### Mendelian randomisation

We did two-sample, bidirectional Mendelian randomisation analyses^63^, ^64^ (appendix p 20) between childhood maltreatment and selected mental health outcomes (schizophrenia,^65^ major depressive disorder,^66^ bipolar disorder,^67^ ADHD,^68^ and autism^69^), physical health conditions (coronary artery disease^70^ and type 2 diabetes^71^), and C-reactive protein as a marker of inflammation,^72^ and corrected with the Bonferroni correction. These phenotypes have all been associated with childhood maltreatment, and their GWAS does not include the UK Biobank, reducing bias in Mendelian randomisation estimates due to sample overlap.^73^ Bidirectional Mendelian randomisation was done using the following methods: inverse variance-weighted Mendelian randomisation, which assumes that all SNPs are valid instruments; median-weighted, which provides valid estimates even if up to 50% of the instruments are invalid;^74^ Mendelian randomisation-Egger, which accounts for pleiotropy by including an intercept term in the inverse variance-weighted model;^75^ and Mendelian randomisation-PRESSO, which accounts for pleiotropy by detecting and removing outliers.^76^ We additionally did leave-one-out analysis as a sensitivity check to investigate it the effects are driven by a subset of the variants.

Scripts and Summary GWAS statistics are available online.

### Role of the funding source

The funders of the study had no role in study design, data analysis, data interpretation, or writing of the report.

## Results

**Figure 1:**
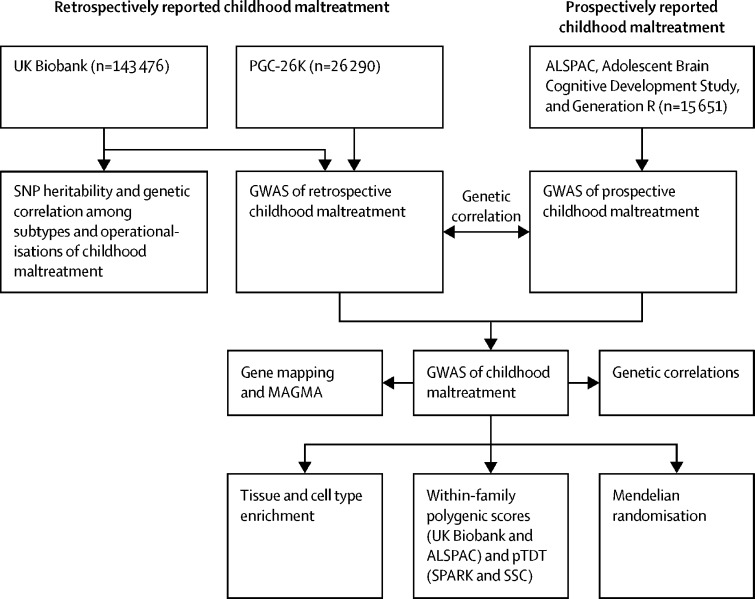
Study profile PGC=Psychiatric Genomics Consortium. ALSPAC=Avon Longitudinal Study of Parents and Children. SNP=single nucleotide polymorphism. GWAS=genome-wide association study. pTDT=polygenic transmission disequilibrium test. SSC=Simons Simplex Collection.

**Figure 2:**
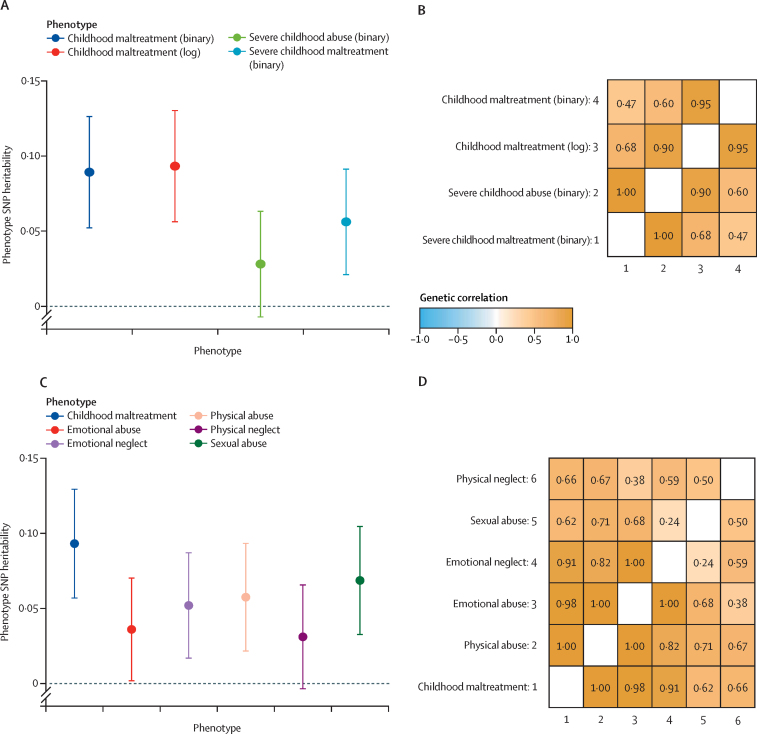
SNP heritability and genetic correlations between phenotypic operationalisations and subtypes of childhood maltreatment (A) SNP heritability of the four phenotypic definitions of childhood maltreatment and 95% CIs (n=19 559). (B) Genetic correlations between the four phenotypic operationalisations of childhood maltreatment (n=19 559). (C) SNP heritability of the subtypes of childhood maltreatment (binarised) alongside the log-transformed sum-score of childhood maltreatment and 95% CIs (n=19 559). (D) Genetic correlations between subtypes of childhood maltreatment and log-transformed sum-score of childhood maltreatment (n=19 559). SNP=single nucleotide polymorphism.

An overview of the study design is provided in figure 1. We did a series of analyses to quantify the heritabilities and genetic correlations of different operationalisations and subtypes of childhood maltreatment in the UK Biobank. Three of the four operationalisations had similar SNP heritability (0·093 [SE 0·019] to 0·056 [0·018], Δ SNP heritability all p values >0·05; figure 2A; see appendix p 63 for individual p values). However, restricting to severe abuse (binarised) identified lower SNP heritability (0·028 [SE 0·018]). All four operationalisations had modest to high genetic correlations with each other (genetic correlation (*r*_g_) 0·47–1·00, figure 2B, appendix p 64), suggesting largely similar common variant genetics between the different operationalisations. We used log-transformed childhood maltreatment scores for subsequent analyses due to the relatively high SNP heritability compared with the other operationalisations of childhood maltreatment and high genetic correlations with other phenotypic operationalisations.

We next investigated the heritability and genetic correlations between subtypes of childhood maltreatment. We identified modest but significant SNP heritability for all five subtypes of childhood maltreatment (figure 2C, appendix p 65). Genetic correlations were high between childhood (overall) maltreatment and subtypes (*r*_g_ 0·62–1·00; figure 2D, appendix p 66). Genetic correlations among the five subtypes themselves ranged from low to high (*r*_g_ 0·24–1·00; figure 2D, appendix p 66).

**Figure 3:**
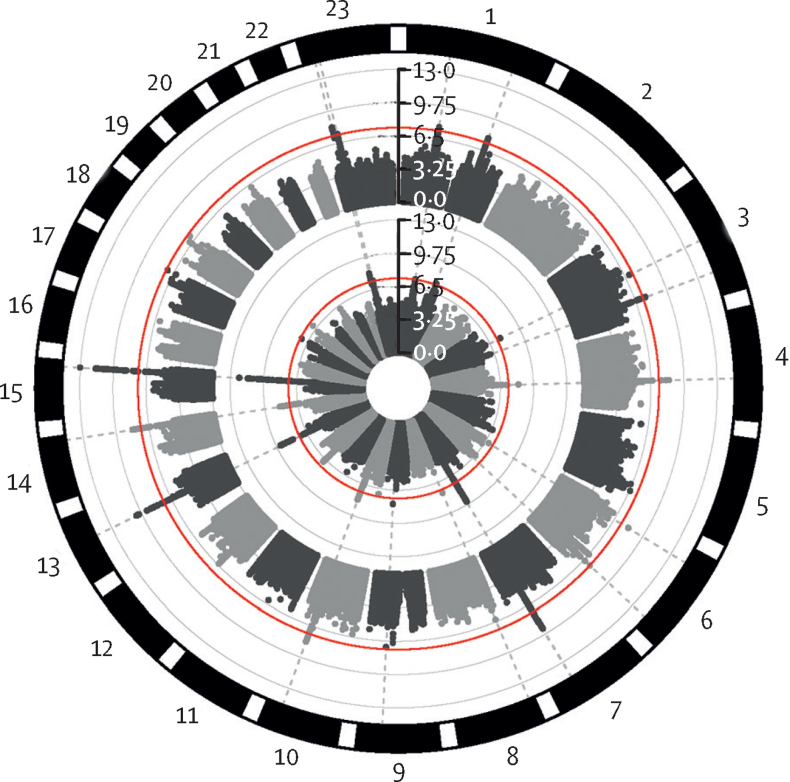
Circular Manhattan plot of childhood maltreatment Circular Manhattan plot for the GWAS meta-analysis of prospectively and retrospectively reported childhood maltreatment (n=185 414, outer ring), and retrospectively reported childhood maltreatment (n=169 766, inner ring). Red lines indicate GWAS significance threshold (p=5 × 10^–8^). The vertical axis provides the p values for single nucleotide polymorphisms included in the GWAS meta-analyses. GWAS=genome-wide association study.

Given the modest to high genetic correlations between operationalisations in the UK Biobank, we asked whether the two retrospective GWASs of childhood maltreatment were genetically correlated. We found a high genetic correlation between the UK Biobank and PGC_26K datasets (*r*_g_=0·64 [SE=0·12]; p=1·15 × 10^–7^). Meta-analysis of the UK Biobank and PGC_26K (GWAS_retrospective_) identified 257 significant SNPs representing 14 independent loci (figure 3, appendix pp 28, 67). In a hold-out sample of 9924 individuals from the UK Biobank, PGS from GWAS_retrospective_ explained a maximum of 0·91% of the variance in retrospectively reported childhood maltreatment (p value threshold=1, p=2 × 10^–16^, appendix p 69). This result represents approximately 10% of the SNP heritability (0·093, or 9·3% of the total phenotypic variance). PGS at all 11 p value thresholds was significant after Benjamini-Hochberg false discovery rate correction. Accounting for Townsend deprivation index (a risk factor for childhood maltreatment)^5^ did not substantially attenuate the variance explained (p value threshold=1, *r*^2^=0·87%; p=2 × 10^–16^). Similarly, we found no change in SNP heritability of childhood maltreatment and subtypes after accounting for Townsend deprivation index (appendix p 64). Thus, the common variant signal for childhood maltreatment is independent of the heritable aspects of social deprivation.^77^

To quantify the shared genetics between prospectively and retrospectively reported childhood maltreatment, we did a GWAS of prospectively reported childhood maltreatment (GWAS_prospective_ n=15 650, SNP heritability 0·046 [SE 0·033]) by meta-analysing data from three datasets (Adolescent Brain Cognitive Development Study, ALSPAC, and Generation R). Genetic correlation between GWAS_retrospective_ and GWAS_prospective_ was high (*r*_g_=0·72 [SE 0·36]; p=0·046), similar to that between the two GWAS of retrospectively reported childhood maltreatment (*r*_g_=0·64 [SE 0·12]). Confirming this result, PGS for GWAS_retrospective_ explained a significant proportion of the variance in prospectively reported childhood maltreatment in ALSPAC across multiple age groups (Nagelkerke's pseudo *r*^2^=0·26%; p=2·04 × 10^–7^, appendix pp 29, 70–71).

Given the relatively high genetic correlation between retrospectively and prospectively reported childhood maltreatment, we did a GWAS meta-analysis of retrospectively and prospectively reported childhood maltreatment (GWAS_childhoodmaltreatment_, UK Biobank, PGC_26K, ALSPAC, Adolescent Brain Cognitive Development Study, and Generation R). We identified 277 genome-wide significant SNPs, representing 14 independent loci (figure 3, appendix pp 30–44, 72). 12 of these lead SNPs had data available in GWAS_prospective_. All showed a concordant effect direction between the GWAS_prospective_ and GWAS_retrospective_ (p=0·0005, binomial sign test), with six loci nominally significant (GWAS_prospective_ p<0·050) and one significant after Bonferroni correction for the 12 loci (rs3851357, GWAS_prospective_ p=0·0035). The meta-analysed GWAS of childhood maltreatment had a significant SNP heritability (0·079 [SE 0·0042], n=185 414), and no inflation in statistics due to unresolved population stratification was detected (LDSC-based intercept 1·0076 [SE 0·0074]). Of the 14 loci, two were on the X-chromosome. Although our study was underpowered for sex-stratified GWAS, we note significant sex differences in overall childhood maltreatment and subtypes in the datasets in which we had access to individual level data. Notably, in most datasets, female individuals were more likely to report childhood maltreatment. In the UK Biobank, female individuals were more likely to report sexual abuse, while male individuals were more likely to report physical abuse in line with previous research (appendix pp 21–22).^78^

The genome-wide significant loci were significantly associated (p<5 × 10^–8^) with mental health problems (six loci), risky behaviour (four loci), smoking and cannabis use (three loci), cardiovascular health, sleep difficulties, and reduced intelligence and educational attainment (two loci each) identified in previous GWASs (appendix pp 73–77).

Positional mapping identified 12 genes, expression quantitative trait loci identified 14 genes, chromatin interaction identified 15 genes (appendix pp 77–82), and MAGMA identified 19 significant genes after Bonferroni correction (appendix p 82). Four of these genes were identified by all four methods: *FES, FOXP2, SORCS3,* and *SAMD5*. The GWAS signal was significantly enriched for foetal and adult brain-specific histone marks and DNAse hypersensitivity sites (appendix p 83), for genes with high expression in the excitatory neuron subpopulation (*Ex4*) in the adult post-mortem brain (appendix p 84), but not genes with tissue-specific expression (appendix pp 85–88).

**Figure 4:**
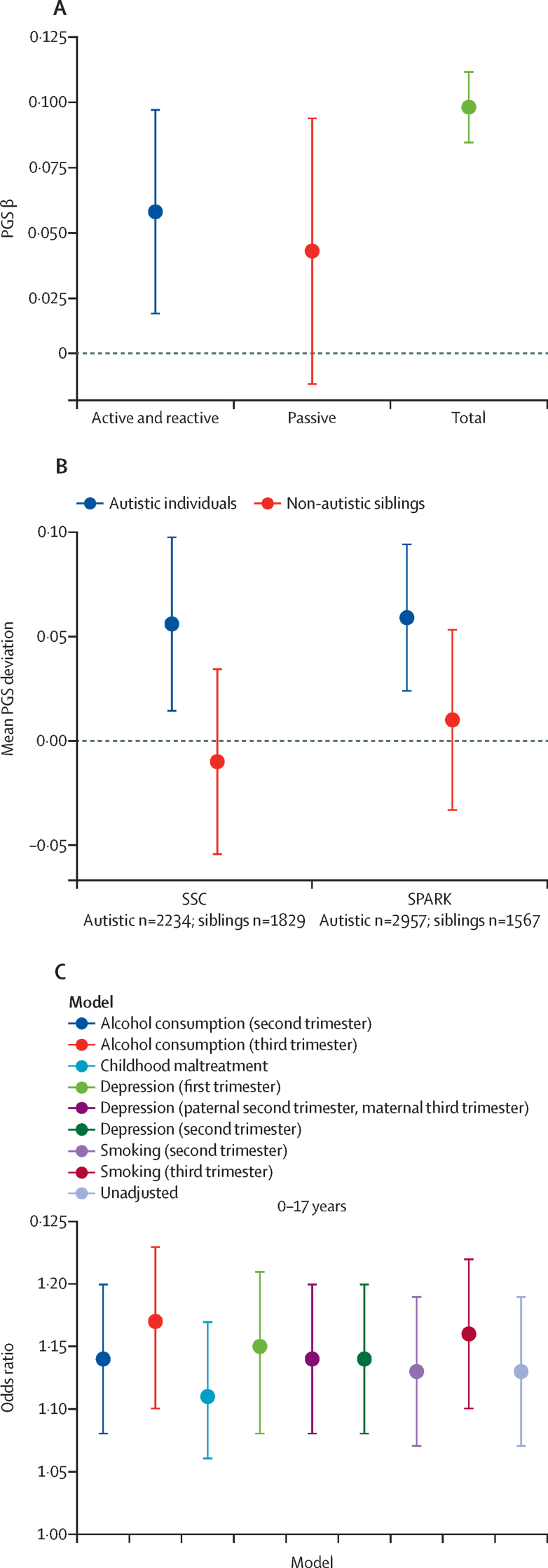
Delineating different gene–environment correlation mechanisms (A) Regression estimates and 95% CIs of active and reactive, passive, and total effects of childhood maltreatment PGS on childhood maltreatment (n=12 855 included 2849 sibling pairs). (B) Polygenic transmission disequilibrium test to investigate over-transmission of childhood maltreatment polygenic scores from parents to autistic children and non-autistic siblings in two cohorts: SSC (n=2234 autistic individuals and 1829 non-autistic siblings) and SPARK (n=2957 autistic individuals and 1567 non-autistic siblings). Mean PGS over-transmission (difference between standardised parental mean PGS and standardised child mean PGS) and 95% CIs provided. (C) Effect of childhood maltreatment PGS on prospectively measured childhood maltreatment (age 0–17 years) in Avon Longitudinal Study of Parents and Children (unadjusted model). The models were adjusted for parental alcohol consumption, parental depression, parental experience of childhood maltreatment, and parental smoking, all measured at the time of pregnancy. Odds ratios and 95% CIs are provided. Sample sizes are provided in the appendix (pp 71–72). PGS=polygenic scores. SSC=Simons Simplex Collection.

To better understand the gene–environment correlation mechanisms that contribute to childhood maltreatment, we did three within-family PGS analyses. First, we compared between-sibling (active and reactive gene–environment correlation) with between-family (ie, total) PGS of GWAS_childhoodmaltreatment_ effects using linear mixed-effects models. The total between-family effect was significant (β=0·095 [SE 0·007]; p<2 × 10^–16^). We identified a small between-sibling effect (β=0·053 [0·02]; p=0·015), accounting for 58% of the total effect. By contrast, the difference in between-family and between-sibling PGS effects (passive gene–environment correlation) did not reach statistical significance (β=0·039 [SE 0·026]; p=0·13) accounting for approximately 42% of the total effect. The difference between the combined active and reactive gene–environment correlation versus passive gene–environment correlation did not reach statistical significance (ΔPGS=0·014 [SE 0·05]; p=0·77; figure 4A).

The between-sibling analyses assumed that parenting (and consequently, familial environment) was similar, or shared, between siblings. However, parenting could systematically differ between, for example, autistic individuals and their non-autistic siblings, in which case the proportion of shared familial environment might be smaller than for families without autistic children. In these families, a larger proportion of sibling difference in childhood maltreatment could be attributable to reactive (eg, poor understanding and support) and active gene–environment correlation (eg, greater risk-taking behaviour, lower social responsiveness) than for siblings with no known neurodevelopmental disorders. Supporting this hypothesis, we found an over-transmission of PGS of GWAS_childhoodmaltreatment_ to autistic children from their parents (mean difference 0·056 [SE 0·02]; p=0·010) but not to their non-autistic siblings (mean difference −0·01 [0·02]; p=0·56) in the Simons Simplex Collection, suggesting that an increased risk for childhood maltreatment in autistic individuals was partly explained by increased active and reactive gene–environment correlation. This finding was replicated in the SPARK dataset (autistic individuals mean difference 0·059 [SE 0·02]; p=1·1 × 10^–3^; non-autistic siblings mean difference 0·01 [0·025]; p=0·65; figure 4B).

We used the family data in ALSPAC to investigate whether accounting for four known parental risk factors (alcohol consumption, childhood maltreatment, depression, and smoking—measured prenatally at multiple timepoints) for childhood maltreatment substantially attenuated the variance explained by PGS for GWAS_retrospective_. None of the four risk factors substantially reduced the variance explained by PGS (figure 4C; appendix pp 29, 71–72).

We next investigated the shared genetics between childhood maltreatment and several health-related phenotypes to contextualise the GWAS_childhoodmaltreatment_ signal. After Bonferroni correction, we identified significant positive genetic correlations between childhood maltreatment and mental health conditions (eg, schizophrenia and depression), anthropometric traits (eg, obesity and body-mass index), insomnia, and coronary artery disease. Childhood maltreatment also had modest negative genetic correlations with intelligence, educational attainment, and age of onset traits (eg, smoking, parental death, first birth; appendix pp 88–93).

**Figure 5:**
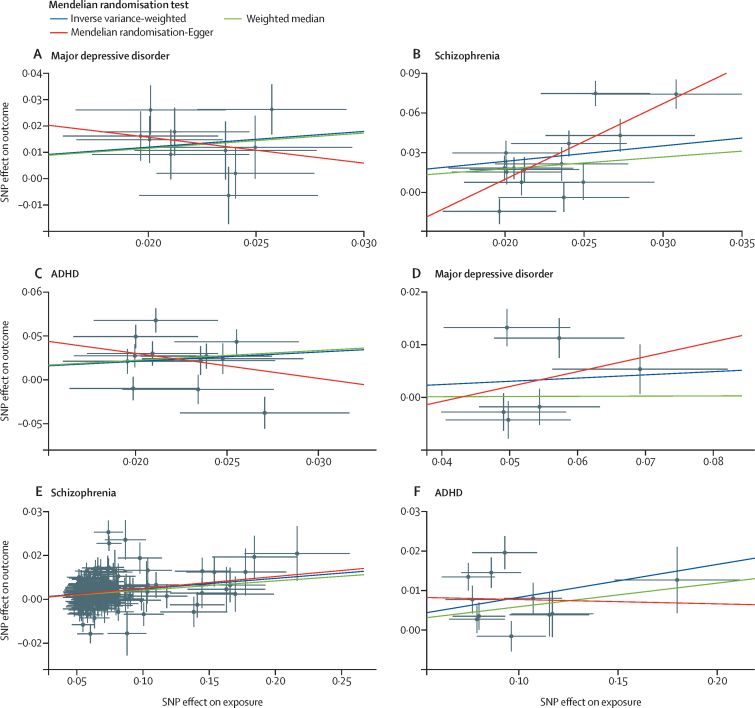
Mendelian randomisation analyses Scatter plots of the SNP effects of childhood maltreatment on major depressive disorder (A), schizophrenia (B), and ADHD (C). Scatter plots of the SNP effects of major depressive disorder (D), schizophrenia (E), and ADHD (F) on childhood maltreatment. All units of associations are log-odds ratios. Slopes provided correspond to three different Mendelian randomisation methods used (inverse variance-weighted, weighted median, and Mendelian randomisation-Egger). The Mendelian randomisation-Egger intercept was significant only for the causal effect of childhood maltreatment on schizophrenia (B; p=0·012). SNP=single nucleotide polymorphism.

To investigate the potential bidirectional causal effect between childhood maltreatment and selected mental and physical health conditions, we did Mendelian randomisation analyses. Inverse variance‑weighted Mendelian randomisation analyses found a significant causal effect of childhood maltreatment on major depressive disorder (figure 5A) but not vice versa (figure 5D). We found a significant bidirectional causal effect for childhood maltreatment with ADHD and schizophrenia (figure 5, appendix p 93). Sensitivity analyses using the weighted median method and Mendelian randomisation-PRESSO were also significant between childhood maltreatment and depression, ADHD, and schizophrenia. Mendelian randomisation-Egger analyses were significant only for the bidirectional effect between childhood maltreatment and schizophrenia (appendix pp 45–60, 93). Leave-one-out analyses did not suggest that any one genetic variant was driving the result, despite the locus-level pleiotropy, and Steiger analyses indicated that the causal direction between the exposure and outcome was correct in all analyses. By contrast, we did not identify a significant causal effect of childhood maltreatment on autism, bipolar disorder, coronary artery disease, type 2 diabetes, C-reactive protein concentration, or vice versa (appendix pp 45–60, 93).

## Discussion

In this study, we found modest SNP heritability and substantial genetic correlations among different operationalisations, subtypes, and reports of childhood maltreatment. This GWAS meta-analysis of childhood maltreatment in 185 414 individuals identified 14 genetic variants (13 novel). Our findings provide some evidence for the role of active and reactive gene–environment correlation in childhood maltreatment, although passive gene–environment correlation could not be excluded. Mendelian randomisation analyses found a unidirectional causal effect of childhood maltreatment on depression, bidirectional effects with ADHD and schizophrenia, but no evidence for unidirectional or bidirectional causal effects on physical health problems.

Childhood maltreatment is modestly heritable, with SNP heritability similar to that of depression.^79^ This SNP heritability provides a lower-bound for the variance attributable to genetic effects. Overall, we identify modest to high genetic correlations among different subtypes, operationalisations, and reports of childhood maltreatment. A 2019 meta-analysis^22^ found low agreement between prospectively and retrospectively reported childhood maltreatment, accounting for chance overlap. However, the raw agreement identified by the same study was 76%. Although it is difficult to compare phenotypic overlap with genetic correlation directly, we believe genetic correlations are more similar to raw agreement as chance overlap is not accounted for in genetic correlation analyses. Furthermore, the proportion of variance attributable to SNPs is modest, suggesting that factors that contribute to relatively low overlap between prospective and retrospective reports might be non-genetic or due to genetic effects not tagged by the SNPs tested.

Genome-wide analyses identified 14 independent loci for childhood maltreatment. The GWAS signal was enriched for regulatory chromatin marks in brain tissues and for genes that are highly expressed in excitatory neurons. These loci are implicated in mental health disorders, risky behaviours, cardiovascular disease, and intelligence, suggesting considerable locus-level pleiotropy. Although we did not test this, the identified genomic variants might interact with exposure to childhood maltreatment to influence epigenetic patterns, thereby contributing to the risk of developing mental and physical health disorders.^80^

Within-family analyses identified significant estimates for active and reactive gene–environment correlation (58%). However, there was no statistical evidence for a difference between passive gene–environment correlation and active and reactive gene–environment correlation. Providing additional evidence for the role of active and reactive gene–environment correlation, childhood maltreatment PGSs are over-transmitted in autistic individuals. It remains unclear which aspects of autism might explain such gene–environment correlation. Importantly, this analysis does not test for the presence or absence of passive gene–environment correlation as all analyses were within-family analyses. Finally, accounting for four known parental risk factors for childhood maltreatment (alcohol consumption, childhood maltreatment, parental depression, and smoking) did not substantially attenuate the variance explained by PGS in prospectively reported childhood maltreatment. These parental risk factors were, however, minimally phenotyped, and we did not have data to test for other parental risk factors (eg, aggression^5^). Together, the analyses support the combined role of active and reactive gene–environment correlation, but do not show the absence of passive gene–environment correlation. Better delineation of the mechanisms of gene–environment correlation can help improve family-based support, for instance, by directly addressing parental mental health in the case of passive gene–environment correlation, or coaching parents on positive discipline methods in the case of reactive and active gene–environment correlation. Further research in larger and more diverse samples is needed to inform interventions.

Given the complex antecedents and consequences of childhood maltreatment, we investigated genetic correlations between childhood maltreatment and several health-related traits. Childhood maltreatment was genetically correlated with some mental health, reproductive, and physical health traits. Notably, childhood maltreatment was negatively genetically correlated with a number of age-of-onset traits, in line with the life history theory—an evolutionary framework suggesting that childhood maltreatment leads to accelerated development and reproduction, with long-term costs for mental and physical health.^81^ However, these genetic correlations do not imply causality as the proposed associations could be due to genetic or environmental confounding or pleiotropy, all of which can be reflected in genetic correlation analyses.

Mendelian randomisation analyses found a potential causal effect of childhood maltreatment on major depressive disorder, and bidirectional causal effects on ADHD and schizophrenia, consistent with observational studies.^14^, ^16^, ^17^ The bidirectional causal effect could be explained by either passive gene–environment correlation (parents of children with ADHD and psychosis have elevated traits in similar domains and might maltreat their children,^5^ in turn contributing to the mental health problems) or by active and reactive gene–environment correlation (PGSs for schizophrenia and ADHD are associated with childhood difficulties,^57^, ^82^ leading to increased risk of maltreatment and subsequent mental health problems). In addition to existing literature using co-twin designs, this study provides evidence for the causal role of childhood maltreatment in some mental health disorders.^24^, ^25^

We did not identify potential causal effects of autism on childhood maltreatment, in contrast to the polygenic transmission disequilibrium test results. This finding might be because the weak instrument used in the autism Mendelian randomisation analysis consisted of only four SNPs or because of the underlying heterogeneity in autism.^83^ The heterogeneity is unlikely to affect within-family studies, but would influence the GWAS hits identified. Finally, the observed polygenic transmission disequilibrium test results could arise if both childhood maltreatment and autism were downstream of a common causal factor, but neither caused the other, an association that need not be reflected in Mendelian randomisation analyses. The current analyses found no causal relationship between childhood maltreatment and physical health conditions, which might be due to the relatively weak instruments (variance in exposure explained by the instruments was low, appendix pp 45–60) or because the observed associations were correlational rather than causal.

There are limitations to this study. First, common genetic variants explain a relatively small proportion of the variance in childhood maltreatment, thus non-genetic factors cannot be ignored. Genetic correlations and gene–environment correlation effects must be interpreted while keeping the modest SNP heritability in mind. For example, although there is a high genetic correlation between childhood maltreatment and some mental health conditions, the covariance will be low due to the modest SNP heritability. Second, maltreatment was not uniformly measured across cohorts, increasing variability due to non-harmonised data collection, including age when maltreatment was measured. Third, we do not distinguish between objective childhood maltreatment and subjective valence of childhood maltreatment.^22^ Fourth, although our study considers childhood maltreatment as a single phenotype (to increase statistical power) and we found considerable genetic correlation between operationalisations, subtypes, and reports of childhood maltreatment, there could be subtype-specific and sex-specific genetic effects. Fifth, individuals with severe mental health disorders are less likely to answer follow-up questionnaires in the UK Biobank,^84^ and the current GWAS reflects ascertainment bias. Finally, our analyses included only individuals of European ancestries due to limitations in data availability.

The disappointing efficacy of current interventions to curb child maltreatment^5^ requires new ideas about potential mechanisms to be targeted as well as biological pathways that might mediate the long-term effect of maltreatment. Our findings of a significant heritability do not imply that environmental factors are absent, that the child is to blame, or that the heritability is fixed. These ideas are discussed in the appendix (pp 93–95). Rather, our findings provide empirical support that the mechanisms underlying maltreatment are complex, reflecting in part the action of multiple forms of gene–environment correlation. The correlation mechanisms investigated here probably contribute to intergenerational transmission of childhood maltreatment, as both immediate environment and genes are inherited by children from parents. These findings highlight the importance of family-based support strategies, targeting parents and their interaction with their children to minimise the risk of child maltreatment, and limiting intergenerational transmission of childhood maltreatment.

## Data sharing

The study website contains details of all data available through a fully searchable data dictionary. Some of these data were collected using REDCap.
