# Correction to *Lancet Psychiatry* 2021; 8: 373–86

**DOI:** 10.1016/S2215-0366(22)00045-1

**Published:** 2022-04

*Warrier V, Kwong ASF, Luo M, et al. Gene–environment correlations and causal effects of childhood maltreatment on physical and mental health: a genetically informed approach.* Lancet Psychiatry *2021;* 8: *373–86*—The appendix of this Article has been corrected as of Feb 8, 2022.

