# Supplementary material for: Gene–environment correlations and causal effects of childhood maltreatment on physical and mental health: a genetically informed approach
Source: Lancet Psychiatry. 2021 May;8(5):373–86. doi: 10.1016/S2215-0366(20)30569-1 (PMC8055541; doi:10.1016/S2215-0366(20)30569-1)
Supplement: Supplementary appendix [file mmc1.pdf]

THE LANCET
Psychiatry

Supplementary appendix

This appendix formed part of the original submission and has been peer reviewed. We post it as supplied by the authors.

Supplement to: Warrier V, Kwong ASF, Luo M, et al. Gene–environment correlations and causal effects of childhood maltreatment on physical and mental health: a genetically informed approach. *Lancet Psychiatry* 2021; published online Mar 16. http://dx.doi.org/10.1016/S2215-0366(20)30569-1.

Contents

Supplementary Methods 1: Participant Characteristics 5

Supplementary Methods 2: Childhood maltreatment measures 7

Supplementary Methods 3: Genetic quality control and genetic association 10

Supplementary Methods 4: GCTA-GREML analyses 13

Supplementary Methods 5: Functional annotation, MAGMA, and enrichment 14

Supplementary Methods 6: Polygenic score analyses 15

Supplementary Methods 7: polygenic Transmission Disequilibrium Tests 19

Supplementary Methods 8: Mendelian Randomization 20

Supplementary Results: Sex differences 21

References 23

Supplementary Figure 1: Frequency histograms of childhood maltreatment and subtypes in the UK Biobank 27

Supplementary Figure 2: Manhattan and quantile-quantile plot of the GWAS meta-analysis of retrospectively reported childhood maltreatment 28

Supplementary Figure 3:Odds ratio and 95% CI of PGS for retrospective childhood maltreatment for having experienced any childhood maltreatment in ALSPAC 29

Supplementary Figure 4: Manhattan and QQplot of the GWAS meta-analysis of childhood maltreatment 30

Supplementary Figure 5: LocusZoom plot for rs12031035 31

Supplementary Figure 6: LocusZoom plot for rs61818983 32

Supplementary Figure 7: LocusZoom plot for rs3851357 33

Supplementary Figure 8: LocusZoom plot for rs611531 34

Supplementary Figure 9: LocusZoom plot for rs4895718 35

Supplementary Figure 10: LocusZoom plot for rs7763390 36

Supplementary Figure 11: LocusZoom plot for rs1859100 37

Supplementary Figure 12: LocusZoom plot for rs4305836 38

Supplementary Figure 13: LocusZoom plot for rs3896224 39

Supplementary Figure 14: LocusZoom plot for rs35560901 40

Supplementary Figure 15: LocusZoom plot for rs77987546 41
Supplementary Figure 16: LocusZoom plot for rs4702 42
Supplementary Figure 17: LocusZoom plot for rs5928362 43
Supplementary Figure 18: LocusZoom plot for rs6633421 44
Supplementary Figure 19: Causal effect of childhood maltreatment on major depressive disorder 45
Supplementary Figure 20: Causal effect of childhood maltreatment on schizophrenia 46
Supplementary Figure 21: Causal effect of childhood maltreatment on ADHD 47
Supplementary Figure 22: Causal effect of childhood maltreatment on autism 48
Supplementary Figure 23: Causal effect of childhood maltreatment on bipolar disorder 49
Supplementary Figure 24: Causal effect of childhood maltreatment on C-Reactive Protein 50
Supplementary Figure 25: Causal effect of childhood maltreatment on coronary artery disease 51
Supplementary Figure 26: Causal effect of childhood maltreatment on Type 2 diabetes 52
Supplementary Figure 27: Causal effect of major depressive disorder on childhood maltreatment 53
Supplementary Figure 28: Causal effect of schizophrenia on childhood maltreatment 54
Supplementary Figure 29: Causal effect of ADHD on childhood maltreatment 55
Supplementary Figure 30: Causal effect of autism on childhood maltreatment 56
Supplementary Figure 31: Causal effect of bipolar disorder on childhood maltreatment 57
Supplementary Figure 32: Causal effect of C-Reactive Protein on childhood maltreatment 58

Supplementary Figure 33: Causal effect of coronary artery disease on childhood maltreatment 59
Supplementary Figure 34: Causal effect of Type 2 diabetes on childhood maltreatment 60
Supplementary Table 1: Participant demographics in all cohort 61
Supplementary Table 2: GCTA-GREML based SNP heritability for operationalizations of childhood maltreatment in the UKBB 63
Supplementary Table 3: GCTA-GREML based genetic correlations between operationalizations of childhood maltreatment in the UKBB 64
Supplementary Table 4: GCTA-GREML based SNP heritability for different subtypes of childhood maltreatment in the UKBB 65
Supplementary Table 5: GCTA-GREML based genetic correlations between subtypes of childhood maltreatment in the UKBB 66
Supplementary Table 6: Lead SNPs from all independent loci with p-value < 1E-6 in the GWAS meta-analysis of retrospectively reported childhood maltreatment 67
Supplementary Table 7: Variance explained by PGS for retrospective childhood maltreatment in a hold-out sample in the UKBB 69
Supplementary Table 8: Variance explained by PGS for retrospective childhood maltreatment in ALSPAC 70
Supplementary Table 9: Lead SNPs from all independent loci with p-value < 5E-08 in the GWAS meta-analysis of childhood maltreatment 72
Supplementary Table 10: GWAS associations of SNPs in the independent loci with other phenotypes 73
Supplementary Table 11: GWAS associations of SNPs in the independent loci with other phenotypes 77
Supplementary Table 12: MAGMA – gene-based association statistics 82
Supplementary Table 13: LDSC-SEG based enrichment analyses for chromatin marks 83
Supplementary Table 14: MAGMA based enrichment analyses for genes with cell-specific expression 84
Supplementary Table 15: MAGMA based enrichment analyses for genes with tissue-specific expression 85

Supplementary Table 16: LDSC based enrichment analyses for genes with tissue-specific expression 87
Supplementary Table 17: Genetic correlation analyses 88
Supplementary Table 18: MR analyses 91
FAQs 93

Supplementary Methods 1: Participant Characteristics

UK Biobank: The UK Biobank is a large, population cohort established to investigate the genetic and non-genetic determinants of health[1]. All individuals included from the UK Biobank were predominantly of European ancestries. We included 143,473 participants from the UK Biobank (Year of birth: 1936 to 1970)[1], as these individuals provided information about childhood maltreatment, and passed the genetic quality control. Mean age of participants, and % of male participants in the UK Biobank are provided in **Supplementary Table 1**. Access to and analysis of de-identified data from the UK Biobank was approved by the Human Biology Research Ethics Committee, University of Cambridge.

PGC_26K: We used summary statistics from 26,290 individuals who were part of the PGC-PTSD dataset. Summary statistics were obtained from Dalvie et al., 2019[2]. This includes 18 different studies with sample sizes ranging from 42 to 7,995. Questionnaires completed, mean age of participants, and % of male participants in the PGC_26K are provided in **Supplementary Table 1**. Access to summary GWAS statistics from the PGC-26K was approved by the Human Biology Research Ethics Committee, University of Cambridge.

ABCD: ABCD is a prospective cohort which was set up to investigate brain development and child health. We conducted GWAS on 5,400 individuals from ABCD[3], all aged between 9 and 10 years at the time of recruitment and survey completion. These were participants who genetically clustered with non-Finnish European populations from the 1000 Genomes phase 3 data, using uMAP clustering based on the first five genetic principal components, and whose genetic sex matched their reported sex, and were not outliers for genetic heterozygosity. The mean age, questionnaires completed, and % of male participants are provided in **Supplementary Table 1.** Access to and analysis of de-identified data from the ABCD was approved by the Human Biology Research Ethics Committee, University of Cambridge.

ALSPAC: We conducted GWAS on 8,346 individuals primarily of European ancestries from the Avon Longitudinal Study of Parents and Children (ALSPAC)[4–6]. ALSPAC is an ongoing longitudinal population-based study that recruited pregnant women residing in Avon (South-West of England) with expected delivery dates between 1st April 1991 and 31st December 1992. The initial number of pregnancies enrolled is 14,541 (for these at least one questionnaire has been returned or a “Children in Focus” clinic had been attended by 19/07/99). Of these initial pregnancies, there was a total of 14,676 foetuses, resulting in 14,062 live births and 13,988 children who were alive at 1 year of age. When the oldest children were approximately 7 years of age, an attempt was made to bolster the initial sample with eligible cases who had failed to join the study originally. As a result, when considering variables collected from the age of seven onwards (and potentially abstracted from obstetric notes) there are data available for more than the 14,541 pregnancies mentioned above. The study website contains details of all data available through a fully searchable data dictionary (http://www.bristol.ac.uk/alspac/researchers/our-data/). Ethical approval for the study was obtained from the ALSPAC Ethics and Law Committee and the Local Research Ethics Committees. Part of this data was collected using REDCap, see the REDCap website for details (https://projectredcap.org/resources/citations/). GWAS data was generated by Sample

Logistics and Genotyping Facilities at Wellcome Sanger Institute and LabCorp (Laboratory Corporation of America) using support from 23andMe. The mean age, questionnaires completed, and % of male participants are provided in **Supplementary Table 1.**

Generation R: The Generation R Study is a population-based prospective cohort study. All children were born between April 2002 and January 2006. This study is designed to identify early environmental and genetic predictors of growth, development, and health from foetal life until young adulthood[7]. We conducted GWAS on 1,905 individuals of Northern European descent from Generation R. The mean age, questionnaires completed, and % of male participants are provided in **Supplementary Table 1.** The Medical Ethical Committee of the Erasmus Medical Centre, Rotterdam has approved the study protocol, and participants have given informed consent in writing.

Supplementary Methods 2: Childhood maltreatment measures

We use the term childhood maltreatment to refer primarily to abuse (physical, emotional, and sexual) and neglect (physical and emotional) that occurs to a child under the age of 18, always by an external agent who is typically in a position of power. Typically, but not always, the external agent is a family member. Due to the nature of the phenotypes used in the current study, we do not restrict our analyses to only questions that pertain to maltreatment at the hands of a family member, as we do not have sufficient information across all the studies included here to distinguish between familial and non-familial sources of maltreatment.

UK Biobank: Childhood maltreatment was retrospectively measured in the UK Biobank using 5 items from the Childhood Trauma Screener (CTS)[8], a retrospective measure of trauma designed for adults and adolescents. Participants completed this questionnaire in adulthood (Ages: 46 – 80). The CTS has good internal consistency (α = 0.75), correlates well with the scales of the longer Childhood Trauma Questionnaire. The CTS covers both abuse (sexual, physical, and emotional) and neglect (emotional and physical). Participants were excluded if they reported 'prefer not to answer'. Each item had options ranging from 'never true' to 'very often true', with scores from 0 to 4.
The items included are:

a. Felt loved as a child (inverse scored) (emotional neglect)
b. Someone to take me to the doctor as a child (inverse scored) (physical neglect)
c. Sexually molested as a child (sexual abuse)
d. Physically abused by family as a child (physical abuse)
e. Felt hated by family member as a child (emotional abuse)

Our primary phenotype was a total sum-score of all five variables, with scores ranging from 0 to 20. This sum-score was log-transformed to account for the skew in the variable. Additionally, we used three other definitions of childhood maltreatment:

1. A binary 'any maltreatment' score, where all scores above 0 were recoded to 1

2. A binary severe childhood maltreatment score, where scores 2 - 4 (equivalent to "Sometimes true", "Often true" and 'Very often true") on any of the five items were recoded to 1, giving a total score ranging from 0 - 1.

3. A binary severe childhood abuse score, where scores 2 - 4 on any of the three abuse items were recoded to 1, giving a total score ranging from 0 - 1

Additionally, we conducted subtype-specific SNP heritability and genetic correlation analyses for all five items. Subtype-specific analysis was conducted after binarizing the scores (1 for ever experiencing childhood trauma, and 0 for never experiencing childhood trauma). We chose to binarize the phenotype as the skew in the scores were not easily amenable to the methods used in the current study. Histograms of the log-transformed childhood maltreatment and binarized subtypes from the UK Biobank are provided in **Supplementary Figure 1**.

ALSPAC: Childhood maltreatment variables (inter-personal violence and neglect) were derived from responses to 121 questions completed by the parents of the participants or by the participants themselves[9,10]. This covered the domains of physical, sexual and emotional abuse, emotional neglect, and additionally, domestic violence and bullying. All variables were collected before the age of 17, with the exception of one questionnaire which was completed by participants at the age of 22 to supplement reports of sexual abuse, emotional neglect and physical abuse collected during the period from 0-17 years. Further information about the variables used is provided elsewhere[9,10]. For measures of child adversity that had several response options related to severity or frequency, we binarized variables to represent exposure to inter-personal violence and neglect throughout childhood and adolescence (0 – 17) that would have been distressing and traumatic for any individual to experience. We divided exposure to inter-personal violence and neglect according to developmental periods: early childhood (0 – 4.9 years), middle childhood (5 – 10.9 years), and adolescence (11 – 17 years). We used childhood inter-personal violence and neglect experienced from 0 – 17 for the GWAS analyses, and all phenotypes (0 – 17, 0 – 4.9, 5 – 10.9, 11 – 17) for PGS analyses.

ABCD: Prospective Childhood maltreatment in ABCD was constructed using 13 questions from Kiddie Schedule for Affective Disorders and Schizophrenia - PTSD (KSADS-PTSD)[11] and the Children's Report of Parental Behavior Inventory (CRPBI)[12]. This was completed by a caregiver when the child was 9 – 10 years of age. We identified three items relating to physical abuse ("shot/stabbed/beaten brutally by a family member", "shot/stabbed/beaten brutally by a non-family member", "Beaten to the point of having bruises by a grown up in the home"), three items relating to sexual abuse ("A grown up in the home touched your child in his or her privates, had your child touch their privates, or did other sexual things to your child", "An adult outside your family touched your child in his or her privates, had your child touch their privates or did other sexual things to your child", and "A peer forced your child to do something sexually"), and two items relating to emotional abuse ("A non-family member threatened to kill your child", and "A family member threatened to kill your child") all from KSADS -PTSD. Additionally, we identified five items pertaining to emotional neglect from CRPBI ("Caregiver makes me feel better after talking over my worries with him/her", "Caregiver smiles at me often", "Caregiver is able to make me feel better when I'm upset", "Caregiver believes in showing his/her love for me", and "Caregiver is easy to talk to"). All items were binarized, with 1 indicating maltreatment, and 0 indicating no maltreatment. The final phenotype was the sum-score of all 13 items, and was rank inverse-normal transformed to account for the skew in the phenotype.

Generation R: Childhood maltreatment variables were derived from a major life events inventory, which asked mothers to indicate whether the child had experienced specific life events at child age 10. The interview in Generation R was based on questionnaires previously used in the TRAILS study and on items in the Life Event and Difficulty Schedule (LEDS)[13]. Exposure to physical violence was defined based on two items: "someone threatened violence to the child" or "someone was violent to the child". Exposure to sexual violence was also defined based on two items: "someone made sexual comments or gestures to the child" or "the child was subject to inappropriate sexual misconduct." Children were coded as exposed to physical violence if either or both items were endorsed. Similarly, children were coded as exposed to sexual violence if either or both items were endorsed. The

total scores of childhood maltreatment were coded as: no maltreatment (0 point), at least one type of maltreatment (1 point), and both types of maltreatment (2 point).

Supplementary Methods 3: Genetic quality control and genetic association

UK Biobank: For the primary phenotype (log-maltreatment) we conducted a genome-wide association analysis using BOLT-LMM[14] , which uses a linear mixed effects model to control for relatedness while increasing statistical power. GWAS was conducted on the autosomes and X chromosome. We excluded individuals who were not of self-reported European Ancestry, or were 5 SDs away from the mean of the first and second genetic principal components in the self-reported European ancestry subset, had a genotyping rate < 95%, were of discordant sex (reported sex did not match genetic sex), and who were outliers for heterozygosity. We included SNPs with minor allele frequency > 0.1%, were in Hardy-Weinberg Equilibrium ($p > 1x10^{-6}$), has a genotyping rate > 90%, and imputation $R^2 > 0.4$. In the GWAS analyses, we included age, sex, the first 20 genetic principal components, and genotype batch as covariates. To calculate relatedness among individuals, we included 1 million SNPs with minor allele frequency > 1%, had a Hardy-Weinberg Equilibrium $p > 1x10^{-6}$, an imputation $R^2 > 0.9$, and genotyping rate > 0.9 in BOLT-LMM[14]. In total, GWAS was conducted for 16,754,618 SNPs covering autosomes and the X chromosome.

ALSPAC: ALSPAC-G1 were genotyped using the Illumina HumanHap550 quad chip genotyping platforms. The resulting raw genome-wide data were subjected to standard quality control methods. Individuals were excluded on the basis of gender mismatches; minimal or excessive heterozygosity; disproportionate levels of individual missingness (>3%) and insufficient sample replication (IBD < 0.8). Population stratification was assessed by multidimensional scaling analysis and compared with Hapmap II (release 22) European descent (CEU), Han Chinese, Japanese and Yoruba reference populations; all individuals with non-European ancestry were removed. SNPs with a minor allele frequency of < 1%, a call rate of < 95% or evidence for violations of Hardy-Weinberg equilibrium ($P < 5x10^{-7}$) were removed. Cryptic relatedness was measured as proportion of identity by descent (IBD > 0.1). Related subjects that passed all other quality control thresholds were retained during subsequent phasing and imputation. 9,115 subjects and 500,527 SNPs passed these quality control filters. We removed SNPs with genotype missingness above 1% due to poor quality (11,396 SNPs removed) and removed a further 321 subjects due to potential ID mismatches. We estimated haplotypes using ShapeIT (v2.r644) which utilises relatedness during phasing. We obtained a phased version of the 1000 genomes reference panel (Phase 1, Version 3) from the Impute2 reference data repository (phased using ShapeIt v2.r644, haplotype release date Dec 2013). Imputation of the target data was performed using Impute V2.2.2 against the reference panel (all polymorphic SNPs excluding singletons), using all 2,186 reference haplotypes (including non-Europeans). We conducted GWAS for autosomes in ALSPAC using BOLT-LMM[14] using sex and age as covariates.

ABCD: Prior to imputation, we undertook stringent quality control of the ABCD dataset. We filtered SNPs with genotyping rate < 90%, excessive and deviations from Hardy Weinberg Equilibrium ($p < 1x10^{-6}$). We removed individuals with genotyping rate < 95%, whose genetic sex did not match their reported sex, and who had excessive heterozygosity. As HWE and heterozygosity are incorrectly calculated in ancestrally mixed populations, these steps were conducted in genetic ancestral groups identified using principal-component based

clustering after combining the data with the 1000 Genomes phase 3 data. Principal components were calculated using GENESIS (http://www.imsbio.co.jp/RGM/R_rdfile?f=GENESIS/man/GENESIS-package.Rd&d=R_BC) after accounting for relatedness between samples as calculated using KING[15]. To identify genetically homogeneous groups, we used the first five principal components calculated to identify clusters in the 1000 Genomes data using UMAP, identifying 7 broad populations - Non-Finnish Europeans, Finnish Europeans, Africans, Americans, East Asians, South Asian, and Bengali. Then, using the first five PCs from the ABCD dataset, we projected individuals onto the seven clusters, identifying broadly homogeneous populations. HWE based filtering ($p < 1x10^{-6}$), and removing individuals with excessive heterozygosity (+/- 3 SD) was then conducted. On this final cleaned data, relatedness was calculated using KING and PCs were calculated after accounting for relatedness, all at the level of individual population categories. The data was then merged, and phased (Eagle v 2.4) and imputed (Minimac4) using the TOPMED Imputation Server[16]. From the imputed data, we removed SNPs with poor imputation ($r^2 < 0.4$), minor allele frequency < 0.1%. GWAS was conducted for all SNPs (N = 24,350,130) using FASTGWA[17] as using the same phenotypes and covariates as described in the UKB analyses. To ensure population stratification does not inflate the effect sizes, we conducted GWAS in individuals of Non-Finnish European ancestry (identified by clustering), and further removed individuals who were 5 SD away from the mean of the first two genetic PCs in this population group. We conducted GWAS for autosomes in individuals of European ancestry (N = 5,400), using the inverse transformed childhood maltreatment score. This was done using FASTGWA which uses a linear mixed effects model accounting for population stratification and relatedness. We included age (in months), sex, and the first 20 genetic principal components as covariates. Scripts for this are available here: https://github.com/vwarrier/ABCD_geneticQC

Generation R: Genotype data was derived from cord blood at birth or from venepuncture during a visit to the research centre on Illumina 610K and 660K genotype arrays (Illumina, San Diego, CA, USA). SNP-level filtering included genotype call rate < 95%, on minor allele frequency (MAF) < 1%, and Hardy-Weinberg Equilibrium (HWE) $p < .000001$. Individuals were filtered based on individual call rate < 95%, outlying heterozygosity, non-European ethnicity, missing phenotype, and relatedness (pairwise IBD >0.185). An extensive description of the calling procedures and subsequent quality control have been described elsewhere[18]. Genotype data that passed quality control were subsequently imputed using the Haplotype Reference Consortium (HRC) release 1.1 as the reference panel. After post-imputation filtering (INFO score < 0.9, MAF <1%), a total number of 5,319,950 SNPs (autosomes only) were available for GWAS. GWAS was conducted with generalized linear model using PLINK2. We included age (in years), sex, batch effect and the first 5 genetic principal components as covariates.

Meta-analyses and variant identification

Sample-size weighted meta-analysis was conducted in METAL[19]. We conducted two meta-analyses. The first was a retrospective childhood maltreatment meta-analysis where we meta-analysed the UK Biobank and the PGC_26K datasets to obtain a GWAS of retrospective childhood maltreatment. Additionally, we meta-analysed all prospective and retrospective

GWAS of childhood maltreatment to obtain a GWAS of childhood maltreatment. Independent significant loci were identified at a GWAS threshold of $p < 5x10^{-8}$, after clumping ($r^2 = 0.1$, 1000 kb), using the LD weights generated from the European subset of the 1000 Genomes Phase 3 dataset[20] in Plink.

Supplementary Methods 4: GCTA-GREML analyses

We used GCTA GREML[21] to investigate the SNP heritability of and genetic correlations between childhood maltreatment phenotypes. First, we generated a random subset of 19,559 unrelated participants from the UK Biobank (--grm-cutoff 0.05) for whom phenotypic data was available. Creating a genetic relatedness matrix is computationally expensive. Our previous analyses indicated that we have the computational resources to create GRMs for a maximum of 20,000 individuals. We thus identified 19,559 unrelated individuals by first creating a random subset of 20,000 individuals who passed our genetic quality control and for whom phenotypic data was available, and then removed related individuals.

Univariate h^2_{SNP} was calculated for different definitions of childhood maltreatment phenotypes and subtypes. Bivariate genetic correlations (r_g) were calculated between the various childhood maltreatment operationalizations, and subtypes. For all analyses, we included year of birth, sex, genotyping batch, and the first 20 genetic principal components as covariates.

Supplementary Methods 5: Functional annotation, MAGMA, and enrichment

We conducted three analyses to identify genes associated with the significant loci. All three were conducted using FUMA[22]. First, we used positional mapping to identify genes that are closest to the lead SNP. Second, we used eQTL data for brain tissues obtained from GTEx[23], BRAINEAC[24], CommonMind Consortium[25] and PsychEncode[26]. Further details are available here: https://fuma.ctglab.nl/tutorial#eQTLs. Finally, we used chromatin interaction data maps from multiple data sources, focussing on chromatin maps from brain tissues. Further details are available here: https://fuma.ctglab.nl/tutorial#chromatin-interactions.

We identified significant genes using MAGMA[27] which incorporates LD information into a regression framework to detect multi-marker effects. MAGMA was conducted using summary GWAS statistics.

We used MAGMA and LDSC-SEG[28] to conduct enrichment analyses. Within FUMA, MAGMA conducts gene set analyses to test if there is an enrichment of GWAS signal for genes with tissue specific expression. This is done using the following formula:

$$Z \sim \beta_0 + Et\beta_E + A\beta_A + B\beta_B + \epsilon$$

Where Z is the gene specific Z score obtained from MAGMA by combining SNP specific p-values. E_t is the expression of a given gene in a specific tissue, A is the average expression across all tissues, and B is the matrix of technical confounders. We tested for enrichment across 53 different genes using gene expression data from GTEx v7. We applied the same method to conduct cell-type specific enrichment. Here, the expression of genes in specific cell types is calculated with average expression across cell types included as a covariate. We used the largest dataset for single cell analyses – the PsychENCODE project[26]. For the adult dataset, this includes data from 27,380 cells. From 15,086 and 17,176 genes, 15,019 and 16,243 genes were mapped to unique ENSG ID for developmental and adult datasets, respectively. For developmental dataset, 4,249 cells were available.

In addition, we also tested for enrichment in genes with brain-specific expression using GTEx data using LDSC-SEG. LDSC-SEG tests for an enrichment in SNP heritability by using stratified LD-score regression. To identify specifically expressed genes, t-statistics are computed comparing the expression of each gene in the brain tissue of interest against the expression of the gene in all other brain tissues. Then, all genes are ranked by their t-statistic and the top 10% of genes are included in the gene set for specifically expressed genes. SNPs are mapped to genes by using 100kb physical dist ance upstream and downstream of the transcription start and end sites respectively. We also additionally investigated enrichment for 489 different cell-type specific chromatin marks, including DNAse-I hypersensitivity sites and histone marks from the ENCODE[29] and the Roadmap Epigenomics projects[30].

Supplementary Methods 6: Polygenic score analyses

UK Biobank: unrelated individuals

We conducted polygenic score analyses in the UK Biobank using unrelated individuals to quantify the variance explained by polygenic scores. To do this, we identified a hold-out sample of sibling-pairs as this group was also useful to quantify the effects of the different forms of rGE (see below). In the UK Biobank, we first identified 22,660 sibling pairs in the UK Biobank using IBS0 > 0.0012 and 0.176 > Kinship coefficient > 0.35. This represents 41,504 unique individuals who are siblings[31]. From this list of unique individuals, we identified 12,855 individuals for whom we had phenotypic data (childhood maltreatment scores) and met all the quality control criteria as outlined in the GWAS. Thus, this represents a group of individuals where phenotypic information is available for at least one of the siblings in the sibling pair. Within this subset of individuals, we identified 2,849 sibling pairs (N = 5,515 individuals) for whom phenotypic information was available for both the siblings. Further, from the 12,885 individuals, we identified 9,924 unrelated individuals using a GRM (--grm-cutoff 0.05) in GCTA[21].

To give us sufficient statistical power to evaluate the variance explained by polygenic score, we excluded the 12,855 individuals and conducted a second GWAS of childhood maltreatment (log-transformed sum-score), resulting in a total sample of 130,618 individuals included in the secondary GWAS. GWAS was conducted as outlined earlier. We first generated polygenic scores in 9,924 unrelated individuals from the 12,855 individuals excluded in the second childhood maltreatment GWAS using PRSice-2[32]. SNPs were pruned using an LD-threshold of 0.1, and 250 kb. We generated polygenic scores at 11 p-value thresholds (p = 1, 0.75, 0.5, 0.25, 0.1, 0.01,$1x10^{-3}$, $1x10^{-4}$, $1x10^{-5}$, $1x10^{-6}$, and $5x10^{-8}$) to identify a threshold that explains maximum variance. We included year of birth, sex, genetic batch and the first 2 0 genetic principal components as covariates (equation 1). In a second model, we additionally included Townsend Deprivation Index as a covariate to evaluate the predictive power of polygenic scores after accounting for deprivation (equation 2).

$$Childhood\_maltreatment_{ij} \sim \beta_1 PGS + Z_{1..n} covariates \quad \text{- (1)}$$

$$Childhood\_maltreatment_{ij} \sim \beta_1 PGS + \beta_2 Deprivation + Z_{1..n} covariates \quad \text{- (2)}$$

UK Biobank: between-sibling analyses

To quantify the variance explained by passive rGE, and active and reactive rGE combined, we simultaneously investigated between-sibling and between-family effects of PGS in a hold-out sample of 12,855 individuals from the UKBB (including 2,849 sibling pairs) using random intercept mixed-effects model. This includes two fixed effects, one which is the difference in PGS between the individual and the mean family PGS (between-siblings), and the other is the mean family PGS (between-family), in line with earlier research[33,34] . The between-sibling PGS indicates that any effect of PGS on childhood maltreatment is because of the sibling's PGS. This maps onto active or reactive rGE, but will be independent of passive rGE. The between-family PGS is an indicator of both individual and familial genetic influences on childhood maltreatment and will reflect all three aspects of rGE (active, reactive, and passive). Thus, the difference between between-family and between-sibling effects will

quantify the proportion of effects attributable to passive rGE. This is given by the equation below.

$$Childhood\_maltreatment_{ij} \sim \beta_{bsib}\left(PGS_{ij} - \overline{PGS_j}\right) + \beta_{bfam}\left(\overline{PGS_j}\right) + Z_{1..n}covariates - (3)$$

Where β_{bsib} is the between-sibling effect of the polygenic scores (representing reactive and active rGE combined), $\beta_{bfam}$is the between-family effects, PGS_j is family-mean PGS for family j. Covariates included were age, sex, genotyping batch and 40 genetic principal components. We include random intercepts for each family. We calculated the standard errors for each fixed effect term, the difference between the between-family and between-sibling estimates by bootstrapping 10,000 times.

We also investigated if complete (where all siblings provided information on childhood maltreatment) and incomplete sibling (where at least one sibling did not provide information on childhood maltreatment) pairs differed in terms of qualifications, sex-ratio, Townsend Deprivation Index, year of birth, and childhood maltreatment scores. There were some differences (Table below), but it is unlikely that this will unduly influence the results as the standard errors in the difference between the between-family and between-sibling estimates have been calculated by bootstrapping.

Supplementary Methods 6: Table 1 – differences between complete and incomplete sibling pairs

	Complete pairs	Incomplete pairs
Year of birth*: Mean (SD)	1951 (6.92)	1951 (6.96)
% Female*	60	58
Townsend Deprivation Index: Mean (SD)	-1.82 (2.73)	-1.77 (2.67)
% with college degree*	57	46
Childhood maltreatment score*: Mean (SD)	7.71 (1.41)	7.63 (1.46)

*p < .001

ALSPAC: unrelated individuals

To further investigate the shared genetics between retrospective and prospective childhood maltreatment, we investigated if PGS from the retrospective GWAS of childhood maltreatment explained a significant proportion of the variance in measures of childhood maltreatment in ALSPAC. Polygenic scores were generated in ALSPAC for a maximum of 7,453 unrelated individuals using PRSice-2[32] (p-value threshold = 1) using PRSice-2. The PGS was standardised to have a mean of 0 and a standard deviation of 1. We included SNPs that had a MAF of > 1% and info score > 80% and excluded SNPs with an R^2 of > 0.1, if they were within 250Kb of each other. We excluded SNPs located in the extended MHC region (chromosome 6 (26-33Mb)). The sample sizes varied between different phenotypes as different ages of

childhood maltreatment had different sample sizes. We regressed the PGS against the binarized exposure to childhood maltreatment at all ages (0 – 17) or at specific ages (0 – 4.9, 5 – 10.9, 11 – 17). We included age in months, sex, and the first 10 principal components as covariates.

To understand if this association primarily reflects passive rGE due to shared genetics with known familial risk factors for childhood maltreatment in ALSPAC, in a subset of participants we additionally included parental depression, parental experience of childhood maltreatment, parental smoking, and parental alcohol consumption as covariates. We ran separate regression models for the four different parental risk factors at all four age groups. These were assessed during pregnancy, and thus are prenatal measures of risk factors. We chose prenatal measures to minimize the effect of parental behaviour being a reaction of child's behaviour, though we note that parental behaviour before and after the birth of a child are often correlated[35–37]. These are:

1. Parental depression assessed in the first trimester: This was measured using a single item which asked: "Have you ever had depression?" with responses being 0 – no and 1 - yes.

2. Parental depressive symptoms assessed in the second trimester: This was assessed using the Edinburgh Postnatal Depression Scale (Cox et al., 1987). We coded responses as 0 (low depressive symptoms) if the scores were less than or equal to 11 and 1 (high depressive symptoms) if scores were greater than or equal to 12.

3. Maternal depressive symptom assessed in the third trimester and paternal depression assessed in the second trimester of pregnancy: Parental depressive symptoms in the third trimester for mothers and second trimester for fathers were assessed using the Edinburgh Postnatal Depression Scale (Cox et al., 1987). We coded responses as 0 (low depressive symptoms) if the scores were less than or equal to 11 and 1 (high depressive symptoms) if scores were greater than or equal to 12. Note fathers' depressive symptoms were only measured once in the antenatal period, hence the second trimester

4. Parental alcohol consumption assessed in the second trimester: This was assessed using a single item that asked: "Have you consumed alcohol in the first 3 months of pregnancy?" with responses coded as 0 – no and 1 – yes (any drinking)."

5. Parental alcohol consumption assessed in the third trimester : This was assessed using a single item which asked: "How many days have you consumed alcohol in the last 2 months of pregnancy?" with responses coded as 0–none and 1–at least one day or more.

6. Parental smoking assessed in the second trimester : This was assessed using a single item which asked: "Have you smoked tobacco in the first 3 months of pregnancy?" with responses coded as 0 – no and 1 – yes (any smoking).

7. Parental smoking assessed in the third trimester of pregnancy: This was assessed using a single item which asked: "How many days have you smoked tobacco in the last 2 months of pregnancy?" with responses coded as 0–no and 1–at least one day or more

8. Parental history of childhood maltreatment, assessed across all trimesters: This was assessed using the several items which asked: “Have you experienced physical cruelty before the age of 17?”, “Have you experienced emotional cruelty before the age of 17?”, “Have you experienced sexual abuse before the age of 17?” with responses 0 – no and 1 – yes. Cases were defined if participants said yes to any of the maltreatment questions.

Supplementary Methods 7: polygenic Transmission Disequilibrium Tests

While between-sibling PGS analyses assumes that a proportion of the familial environment is shared between siblings, this may not always be the case. A case in point is siblings who are discordant for neurodevelopmental conditions such as autism or ADHD. Because of the different support needs of the siblings, parental response to the two siblings will be very different. This indexes reactive rGE, and to an extent active rGE. To quantify this, we conducted polygenic Transmission Disequilibrium Tests (pTDT)[38] in two cohorts (Simons Simplex Collection (SSC)[39] and SPARK[40]). In both cohorts, we restricted it to participants who were primarily of European ancestries (using genetic multidimensional scaling) in the SSC and using uMAP clustering in SPARK. PGS were created using PRSice2[32] as stated above, using a p-value threshold of 1 as this explained the highest variance in PGS in the UK Biobank. Quality control of the SSC cohort is provided elsewhere[41]. Quality control and imputation of the SPARK cohort was conducted using the same pipeline as used for the ABCD cohort. pTDT is a within-family method which is a modified t-test that compares the mean PGS in autistic individuals compared to the mean mid-parent PGS. As it is a within-family method, it is not confounded by population stratification or assortative mating.

Supplementary Methods 8: Mendelian Randomization

We conducted Mendelian Randomization (MR) analyses to investigate the potential causal effects of childhood maltreatment on selected mental health conditions (ADHD[42], autism[43], bipolar disorder[44], major depressive disorder[45], and schizophrenia[46]), selected physical health conditions (type 2 diabetes[47] and coronary artery disease[48]), and C-reactive protein[49] as a marker of inflammation. These GWAS were selected as: 1. Their samples did not overlap with the samples included in the GWAS of childhood maltreatment, minimizing bias; 2. There is either moderate shared genetics between these conditions and/or there is empirical evidence to suggest that people exposed to childhood maltreatment have an increased risk for developing these conditions[50–52].

We conducted two-sample bidirectional MR using summary GWAS statistics using the Two-sample MR package in R (https://mrcieu.github.io/TwoSampleMR/articles/index.html). This package harmonizes exposure and outcome data and ensures that the SNPs are strand aligned where possible. For all MR analyses, we created instruments using independent GWAS loci with a p-value < $5x10^{-8}$. The only exception was the GWAS of major depressive disorders. We used summary stats for major depressive disorder which excluded participants from the UK Biobank (downloaded from: https://www.med.unc.edu/pgc/download-results/mdd/). Only two GWAS loci were significant, so when the GWAS was used to create the instrument for the exposure, we included 6 SNPs with p-value < $1x10^{-7}$.

For each MR analyses, we first conducted inverse variance-weighted (IVW) meta-analysis, which is a fixed-effect meta-analyses that translates to a weighted regression of the effects of the SNPs in the exposure against the effects of the same SNPs in the outcome. To account for horizontal pleiotropy, we conducted weighted median MR, which is a majority-valid method of MR[53]. This provides a reliable estimate even if 50% of the instruments are invalid. We also conducted MR-Egger analyses[54], which is a modification of the IVW analyses, but allows for the intercept to deviate from 0. However, MR-Egger reduces statistical power and requires the InSIDE assumption to hold. We also investigated if the intercept in the MR-Egger analyses significantly deviates from 0, which is an indicator of average bias due to pleiotropy. Finally, we also conducted MR-PRESSO (Pleiotropy Residual Sum and Outlier)[55] analyses to detect and exclude outliers in the instruments which likely represent pleiotropic effects. We identified instruments as outliers if they had a p-value < 0.05 in the test for outliers as incorporated in MR-PRESSO.

Given the substantial shared genetics between childhood maltreatment and the various phenotypes included, we investigated if the instruments actually represent the right causal direction using Steiger analyses[56]. This investigates if the instrument explains a greater proportion of the variance in the exposure compared to the outcome. Finally, we also conducted leave-one-out analyses to investigate if the effects are driven by one or a subset of the SNPs. These analyses provide additional robustness to our primary results.

Supplementary Results: Sex differences

We investigated sex differences in childhood maltreatment in the UK Biobank, ALSPAC, ABCD, and Generation R. Additionally, we investigated sex differences in subtypes in the UK Biobank.

UK Biobank

In the UK Biobank, females were significantly more likely to indicate higher scores on childhood maltreatment and all subtype measures except physical abuse and emotional neglect. For physical abuse, males were significantly more likely to indicate physical abuse. No sex difference was identified for emotional neglect. Means and standard deviations are provided for the continuous scores of childhood maltreatment and subtypes. We conducted Students T-tests to investigate sex differences.

Supplementary Results: Table 1 – Sex differences in childhood maltreatment in UKBB

	**Females: Mean (SD)**	**Males: Mean (SD)**	**t**	**p-value**
n	80,608	62,865		
Childhood maltreatment	1.80 (2.52)	1.60 (2.11)	15.71	< .001
Emotional neglect	0.75 (0.98)	0.76 (0.94)	-1.63	.1029
Physical neglect	0.27 (0.73)	0.24 (0.71)	7.44	< .001
Emotional abuse	0.33 (0.81)	0.21 (0.64)	29.92	< .001
Physical abuse	0.26 (0.65)	0.30 (0.66)	-11.58	< .001
Sexual abuse	0.18 (0.58)	0.08 (0.37)	38.23	< .001

ALSPAC

We identified a significant sex-difference in childhood trauma between the ages of 5 – 10 and 10 – 17, with females more likely to indicate experiencing maltreatment. However, we do not identify statistically significant sex difference between ages 0 – 5 or overall. We conducted chi-square tests on binarized measures of childhood maltreatment. Results are provided below.

Supplementary Results: Table 2 – Sex differences in childhood maltreatment in ALSPAC

	**0-5 years**			**5-10 years**		
	No	Yes	χ2 test	No	Yes	χ^2 test
n	10880	2686		7657	3660	
Female	5614	1387	$\chi^2(1) = 0.001, p = .971$	3817	1984	$\chi^2(1) = 18.823, p < .001$
Male	5266	1299		3840	1676	
	**10-17 years**			**0-17 years**		
	No	Yes	Chi-Square test	No	Yes	Chi-Square test
n	6880	2681		7488	6143	

Female	3473	1238	$\chi^2(1)$ = 14.291, < .001	3873	3157	$\chi^2(1)$ = 0.148, *p* = .70
Male	3407	1443		3615	2986	

ABCD

In ABCD, we identify significant sex differences in childhood maltreatment, with males more likely to report childhood maltreatment. We report the mean and standard deviation for the continuous score of childhood maltreatment. Students T-test was conducted to investigate sex differences.

Supplementary Results: Table 3 – Sex differences in childhood maltreatment in ABCD

	Females: Mean (SD)	Males: Mean (SD)		
n	2,531	2,869	t	p-value
Childhood maltreatment	1.65 (2.40)	2.09 (2.64)	-6.3345	< 0.001

Generation R

We identify significant sex differences in childhood maltreatment in Generation R, with females experiencing higher childhood maltreatment. We conducted chi-square tests on counts of childhood maltreatment.

Supplementary Results: Table 4 – Sex differences in childhood maltreatment in Generation R

	No maltreatment	At least one type	Both types	χ^2 test
n	1548	337	20	
Female	831	130	8 (40)	$\chi^2(2) = 26.23$, $p < .001$
Male	717	207	12 (60)	

References

1. Sudlow C, Gallacher J, Allen N, et al. UK Biobank: An Open Access Resource for Identifying the Causes of a Wide Range of Complex Diseases of Middle and Old Age. *PLOS Med*. 2015;12(3):e1001779. doi:10.1371/journal.pmed.1001779
2. Dalvie S, Maihofer AX, Coleman JRI, et al. Genomic influences on self-reported childhood maltreatment. *Transl Psychiatry*. 2020;10(1):1234567890. doi:10.1038/s41398-020-0706-0
3. Casey BJ, Cannonier T, Conley MI, et al. The Adolescent Brain Cognitive Development (ABCD) study: Imaging acquisition across 21 sites. *Dev Cogn Neurosci*. March 2018. doi:10.1016/J.DCN.2018.03.001
4. Fraser A, Macdonald-Wallis C, Tilling K, et al. Cohort Profile: the Avon Longitudinal Study of Parents and Children: ALSPAC mothers cohort. *Int J Epidemiol*. 2013;42(1):97-110. doi:10.1093/ije/dys066
5. Northstone K, Lewcock M, Groom A, et al. The Avon Longitudinal Study of Parents and Children (ALSPAC): an update on the enrolled sample of index children in 2019 [version 1; peer review: 2 approved]. *Wellcome Open Res*. 2019;4. doi:10.12688/wellcomeopenres.15132.1
6. Boyd A, Golding J, Macleod J, et al. Cohort Profile: The "Children of the 90s"—the index offspring of the Avon Longitudinal Study of Parents and Children. *Int J Epidemiol*. 2013;42(1):111-127. doi:10.1093/ije/dys064
7. Jaddoe VWV, Van Duijn CM, Van Der Heijden AJ, et al. The generation R study: Design and cohort update until the age of 4 years. *Eur J Epidemiol*. 2008;23(12):801-811. doi:10.1007/s10654-008-9309-4
8. Glaesmer H, Schulz A, Häuser W, Freyberger H, Brähler E, Grabe H-J. Der Childhood Trauma Screener (CTS) - Entwicklung und Validierung von Schwellenwerten zur Klassifikation. *Psychiatr Prax*. 2013;40(4):220-226. doi:10.1055/s-0033-1343116
9. Croft J, Heron J, Teufel C, et al. Association of Trauma Type, Age of Exposure, and Frequency in Childhood and Adolescence with Psychotic Experiences in Early Adulthood. *JAMA Psychiatry*. 2019;76(1):79-86. doi:10.1001/jamapsychiatry.2018.3155
10. Houtepen LC, Heron J, Suderman MJ, Tilling K, Howe LD. Adverse childhood experiences in the children of the avon longitudinal study of parents and children (ALSPAC) [version 1; referees: 3 approved]. *Wellcome Open Res*. 2018;3:106. doi:10.12688/wellcomeopenres.14716.1
11. Geller B, Zimerman B, Williams M, et al. Reliability of the Washington University in St. Louis Kiddie Schedule for Affective Disorders and Schizophrenia (WASH-U-KSADS) Mania and Rapid Cycling Sections. *J Am Acad Child Adolesc Psychiatry*. 2001;40(4):450-455. doi:10.1097/00004583-200104000-00014
12. Schaefer ES. Children's Reports of Parental Behavior: An Inventory. *Child Dev*. 1965;36(2):413. doi:10.2307/1126465
13. Dunn EC, Nishimi K, Neumann A, et al. Time-Dependent Effects of Exposure to Physical and Sexual Violence on Psychopathology Symptoms in Late Childhood: In Search of Sensitive Periods in Development. *J Am Acad Child Adolesc Psychiatry*. 2020;59(2):283-295.e4. doi:10.1016/j.jaac.2019.02.022
14. Loh P-R, Kichaev G, Gazal S, Schoech AP, Price AL. Mixed-model association for biobank-scale datasets. *Nat Genet*. 2018;50(7):906-908. doi:10.1038/s41588-018-0144-6

15. Manichaikul A, Mychaleckyj JC, Rich SS, Daly K, Sale M, Chen W-M. Robust relationship inference in genome-wide association studies. *Bioinformatics*. 2010;26(22):2867-2873. https://www.ncbi.nlm.nih.gov/pmc/articles/PMC3025716/. Accessed August 10, 2020.
16. Kowalski MH, Qian H, Hou Z, et al. Use of >100,000 NHLBI Trans-Omics for Precision Medicine (TOPMed) Consortium whole genome sequences improves imputation quality and detection of rare variant associations in admixed African and Hispanic/Latino populations. *PLoS Genet*. 2019;15(12):e1008500. doi:10.1371/journal.pgen.1008500
17. Jiang L, Zheng Z, Qi T, et al. A resource-efficient tool for mixed model association analysis of large-scale data. *Nat Genet*. 2019;51(12):1749-1755. doi:10.1038/s41588-019-0530-8
18. Medina-Gomez C, Felix JF, Estrada K, et al. Challenges in conducting genome-wide association studies in highly admixed multi-ethnic populations: the Generation R Study. *Eur J Epidemiol*. 2015;30(4):317-330. doi:10.1007/s10654-015-9998-4
19. Willer CJ, Li Y, Abecasis GR. METAL: fast and efficient meta-analysis of genomewide association scans. *Bioinformatics*. 2010;26(17):2190-2191. doi:10.1093/bioinformatics/btq340
20. Delaneau O, Marchini J, McVean GA, et al. Integrating sequence and array data to create an improved 1000 Genomes Project haplotype reference panel. *Nat Commun*. 2014;5:3934. doi:10.1038/ncomms4934
21. Yang J, Lee SH, Goddard ME, Visscher PM. GCTA: a tool for genome-wide complex trait analysis. *Am J Hum Genet*. 2011;88(1):76-82. doi:10.1016/j.ajhg.2010.11.011
22. Watanabe K, Taskesen E, van Bochoven A, Posthuma D. Functional mapping and annotation of genetic associations with FUMA. *Nat Commun*. 2017;8(1):1826. doi:10.1038/s41467-017-01261-5
23. Lonsdale J, Thomas J, Salvatore M, et al. The Genotype-Tissue Expression (GTEx) project. *Nat Genet*. 2013;45(6):580-585. doi:10.1038/ng.2653
24. Ramasamy A, Trabzuni D, Guelfi S, et al. Genetic variability in the regulation of gene expression in ten regions of the human brain. *Nat Neurosci*. 2014;17(10):1418-1428. doi:10.1038/nn.3801
25. Hoffman GE, Bendl J, Voloudakis G, et al. CommonMind Consortium provides transcriptomic and epigenomic data for Schizophrenia and Bipolar Disorder. *Sci data*. 2019;6(1):180. doi:10.1038/s41597-019-0183-6
26. Akbarian S, Liu C, Knowles JA, et al. The PsychENCODE project. *Nat Neurosci*. 2015;18(12):1707-1712. doi:10.1038/nn.4156
27. de Leeuw CA, Mooij JM, Heskes T, Posthuma D. MAGMA: generalized gene-set analysis of GWAS data. *PLoS Comput Biol*. 2015;11(4):1-19. doi:10.1371/journal.pcbi.1004219
28. Finucane HK, Reshef YA, Anttila V, et al. Heritability enrichment of specifically expressed genes identifies disease-relevant tissues and cell types. *Nat Genet*. 2018;50(4):621-629. doi:10.1038/s41588-018-0081-4
29. Bernstein BE, Birney E, Dunham I, Green ED, Gunter C, Snyder M. An integrated encyclopedia of DNA elements in the human genome. *Nature*. 2012;489(7414):57-74. doi:10.1038/nature11247
30. Roadmap Epigenomics Consortium, Kundaje A, Meuleman W, et al. Integrative analysis of 111 reference human epigenomes. *Nature*. 2015;518(7539):317-329.

doi:10.1038/nature14248
31. Bycroft C, Freeman C, Petkova D, et al. The UK Biobank resource with deep phenotyping and genomic data. *Nature*. 2018;562(7726):203-209. doi:10.1038/s41586-018-0579-z
32. Choi SW, O'Reilly PF. PRSice-2: Polygenic Risk Score software for biobank-scale data. *Gigascience*. 2019;8(7). doi:10.1093/GIGASCIENCE/GIZ082
33. Demange PA, Hottenga JJ, Abdellaoui A, et al. Parental influences on offspring education: indirect genetic effects of non-cognitive skills. *bioRxiv*. September 2020:2020.09.15.296236. doi:10.1101/2020.09.15.296236
34. Selzam S, Ritchie SJ, Pingault JB, Reynolds CA, O'Reilly PF, Plomin R. Comparing Within- and Between-Family Polygenic Score Prediction. *Am J Hum Genet*. 2019;105(2):351-363. doi:10.1016/j.ajhg.2019.06.006
35. Kendler KS, Gardner CO, Edwards A, et al. Dimensions of Parental Alcohol Use/Problems and Offspring Temperament, Externalizing Behaviors, and Alcohol Use/Problems. *Alcohol Clin Exp Res*. 2013;37(12):2118-2127. doi:10.1111/acer.12196
36. Håberg SE, Stigum H, Nystad W, Nafstad P. Effects of pre- and postnatal exposure to parental smoking on early childhood respiratory health. *Am J Epidemiol*. 2007;166(6):679-686. doi:10.1093/aje/kwm134
37. Pearson RM, Evans J, Kounali D, et al. Maternal depression during pregnancy and the postnatal period risks and possible mechanisms for offspring depression at age 18 years. *JAMA Psychiatry*. 2013;70(12):1312-1319. doi:10.1001/jamapsychiatry.2013.2163
38. Weiner DJ, Wigdor EM, Ripke S, et al. Polygenic transmission disequilibrium confirms that common and rare variation act additively to create risk for autism spectrum disorders. *Nat Genet*. May 2017. doi:10.1038/ng.3863
39. Fischbach GD, Lord C. The Simons Simplex Collection: A Resource for Identification of Autism Genetic Risk Factors. *Neuron*. 2010;68(2):192-195. doi:10.1016/J.NEURON.2010.10.006
40. Feliciano P, Daniels AM, Snyder LG, et al. SPARK: A US Cohort of 50,000 Families to Accelerate Autism Research. *Neuron*. 2018;97(3):488-493. doi:10.1016/j.neuron.2018.01.015
41. Warrier V, Toro R, Won H, et al. Social and non-social autism symptoms and trait domains are genetically dissociable. *Commun Biol*. 2019;2(1):328. doi:10.1038/s42003-019-0558-4
42. Demontis D, Walters RK, Martin J, et al. Discovery of the first genome-wide significant risk loci for attention deficit/hyperactivity disorder. *Nat Genet*. 2019;51(1):63-75. doi:10.1038/s41588-018-0269-7
43. Grove J, Ripke S, Als TD, et al. Identification of common genetic risk variants for autism spectrum disorder. *Nat Genet*. 2019;51(3):431-444. doi:10.1038/s41588-019-0344-8
44. Stahl EA, Breen G, Forstner AJ, et al. Genome-wide association study identifies 30 loci associated with bipolar disorder. *Nat Genet*. 2019;51(5):793-803. doi:10.1038/s41588-019-0397-8
45. Wray NR, Ripke S, Mattheisen M, et al. Genome-wide association analyses identify 44 risk variants and refine the genetic architecture of major depression. *Nat Genet*. 2018;50(5):668-681. doi:10.1038/s41588-018-0090-3
46. Lam M, Chen C-Y, Li Z, et al. Comparative genetic architectures of schizophrenia in

East Asian and European populations. *Nat Genet*. November 2019:1-9. doi:10.1038/s41588-019-0512-x
47. Maier RM, Zhu Z, Lee SH, et al. Improving genetic prediction by leveraging genetic correlations among human diseases and traits. *Nat Commun*. 2018;9(1):989. doi:10.1038/s41467-017-02769-6
48. the CARDIoGRAMplusC4D Consortium. A comprehensive 1,000 Genomes-based genome-wide association meta-analysis of coronary artery disease. *Nat Genet*. 2015;47(10). doi:10.1038/NG.3396
49. Wojcik GL, Graff M, Nishimura KK, et al. Genetic analyses of diverse populations improves discovery for complex traits. *Nature*. 2019;570(7762):514-518. doi:10.1038/s41586-019-1310-4
50. Shields ME, Hovdestad WE, Pelletier C, Dykxhoorn JL, O'Donnell SC, Tonmyr L. Childhood maltreatment as a risk factor for diabetes: findings from a population-based survey of Canadian adults. *BMC Public Health*. 2016;16(1):879. doi:10.1186/s12889-016-3491-1
51. Gilbert R, Widom CS, Browne K, Fergusson D, Webb E, Janson S. Burden and consequences of child maltreatment in high-income countries. *Lancet*. 2009;373(9657):68-81. doi:10.1016/S0140-6736(08)61706-7
52. Chandan JS, Thomas T, Gokhale KM, Bandyopadhyay S, Taylor J, Nirantharakumar K. The burden of mental ill health associated with childhood maltreatment in the UK, using The Health Improvement Network database: a population-based retrospective cohort study. *The Lancet Psychiatry*. 2019;6(11):926-934. doi:10.1016/S2215-0366(19)30369-4
53. Bowden J, Davey Smith G, Haycock PC, Burgess S. Consistent Estimation in Mendelian Randomization with Some Invalid Instruments Using a Weighted Median Estimator. *Genet Epidemiol*. 2016;40(4):304-314. doi:10.1002/gepi.21965
54. Bowden J, Davey Smith G, Burgess S. Mendelian randomization with invalid instruments: effect estimation and bias detection through Egger regression. *Int J Epidemiol*. 2015;44(2):512-525. doi:10.1093/ije/dyv080
55. Verbanck M, Chen CY, Neale B, Do R. Detection of widespread horizontal pleiotropy in causal relationships inferred from Mendelian randomization between complex traits and diseases. *Nat Genet*. 2018;50(5):693-698. doi:10.1038/s41588-018-0099-7
56. Hemani G, Tilling K, Davey Smith G. Orienting the causal relationship between imprecisely measured traits using GWAS summary data. Li J, ed. *PLOS Genet*. 2017;13(11):e1007081. doi:10.1371/journal.pgen.1007081

Supplementary Figure 1: Frequency histograms of childhood maltreatment and subtypes in the UK Biobank

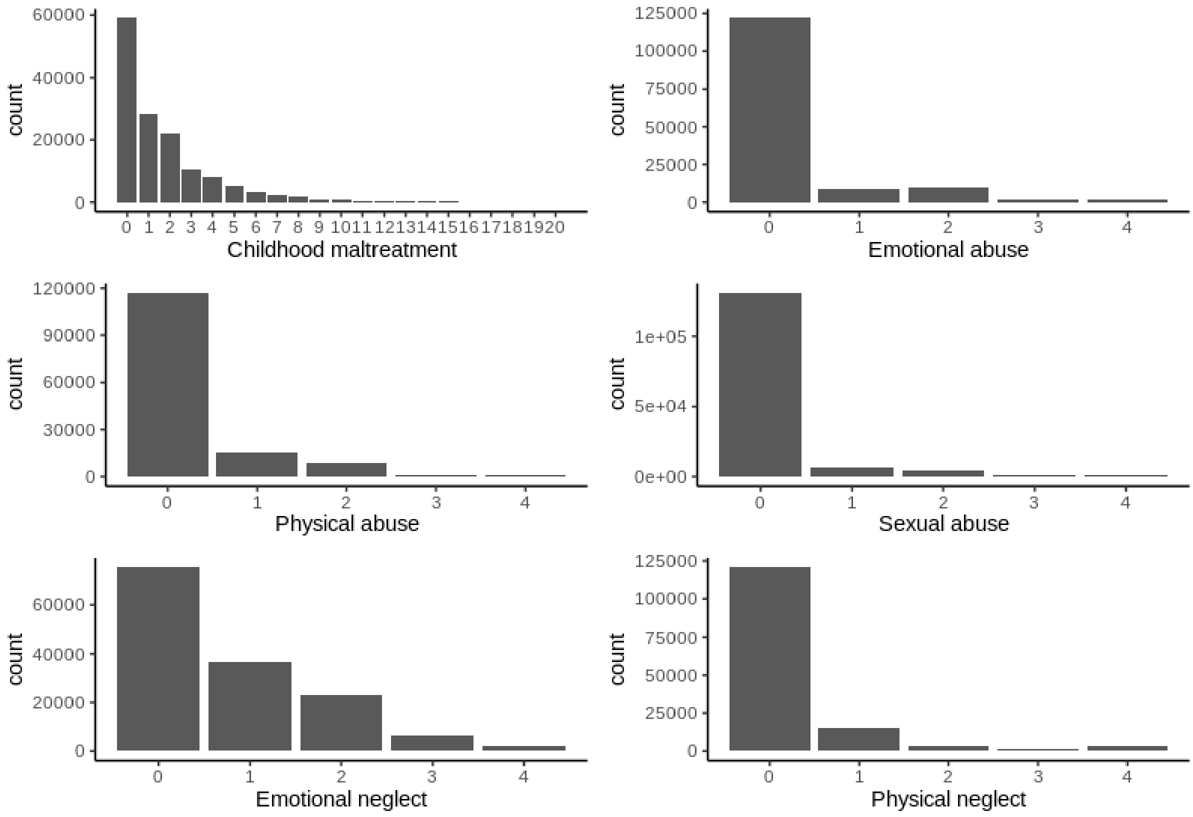

Frequency histogram of childhood maltreatment and subtypes in the UKBB. None of the data has been transformed in this figure. We log-transformed childhood maltreatment and binarized the subtypes for the GWAS analyses.

Supplementary Figure 2: Manhattan and quantile-quantile plot of the GWAS meta-analysis of retrospectively reported childhood maltreatment

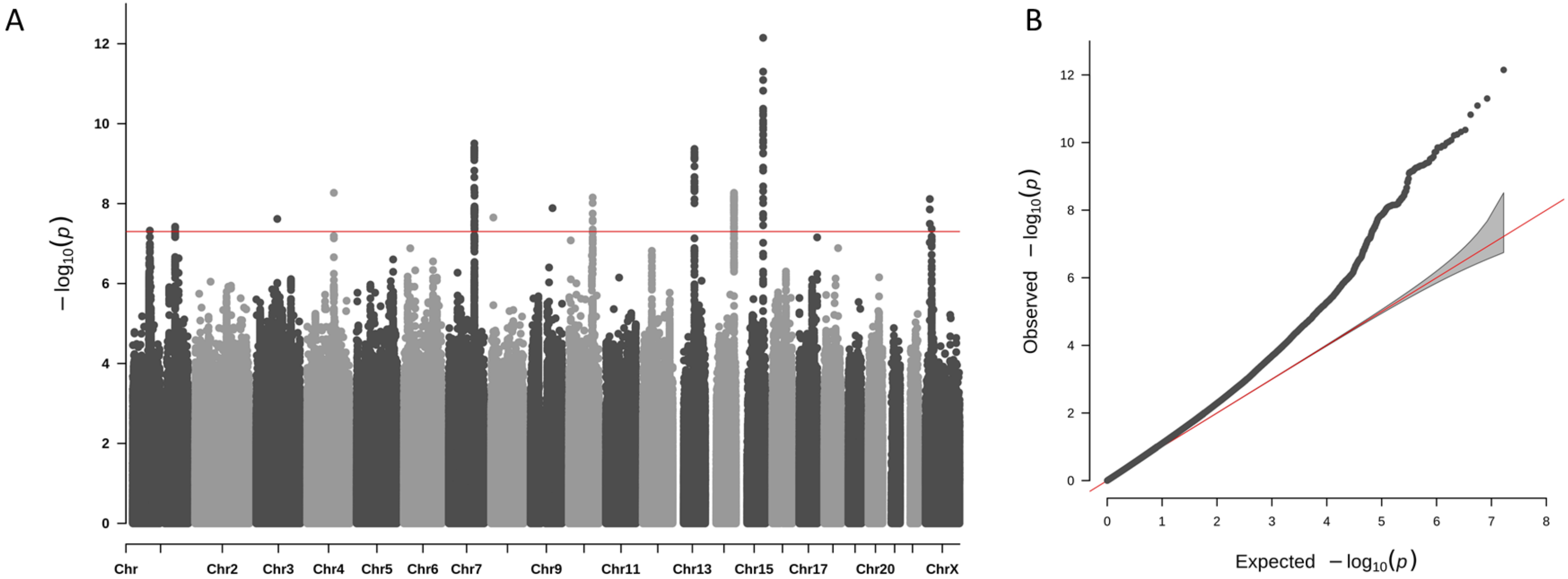

Manhattan plot (A) and quantile-quantile plot (B) of the GWAS meta-analysis of retrospectively reported childhood maltreatment .

Supplementary Figure 3:Odds ratio and 95% CI of PGS for retrospective childhood maltreatment for having experienced any childhood maltreatment in ALSPAC

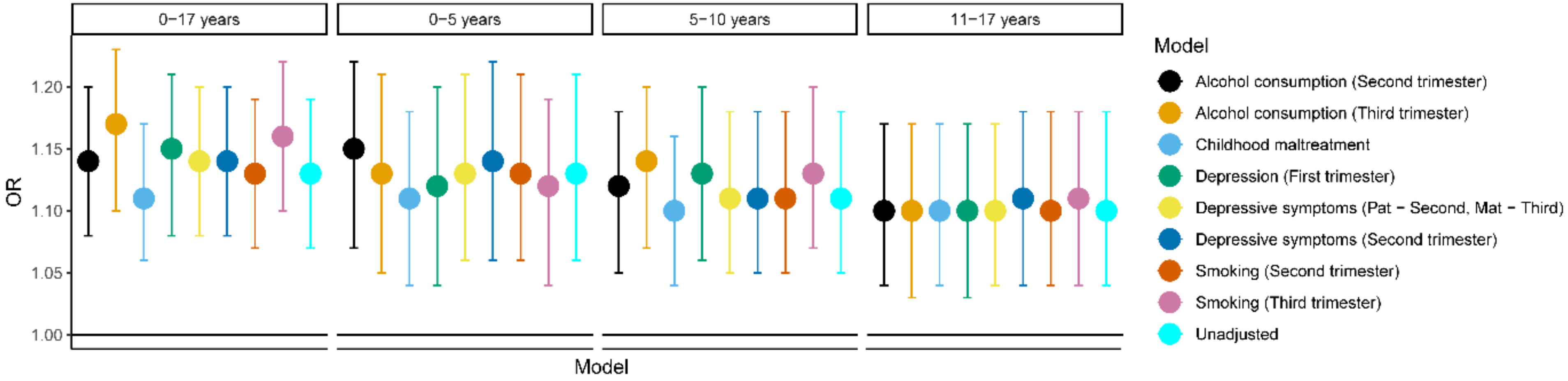

Odds Ratios and 95% CI interval for polygenic scores from retrospective childhood maltreatment being associated with having experienced any childhood maltreatment in ALSPAC at four different age ranges (0 – 17, 0 – 4.9, 5 – 10.9, and 11 – 17). Odds Ratios were calculated after taking the log of the regression beta values. In the unadjusted models, age, sex, and the first five genetic principal components were included as covariates across the different age ranges (0 – 17 [$N_{unadjusted}$ = 7453], 0 – 4.9 [$N_{unadjusted}$ = 7424], 5 – 10.9 [$N_{unadjusted}$ = 6881], and 11 – 17 [$N_{unadjusted}$ = 6303]). In the adjusted models, we additionally adjusted for parental prenatal depression, alcohol consumption, childhood maltreatment, and smoking assessed at different trimesters, which are provided in parenthesis. Sample sizes are provided in Supplementary Table 8.

Supplementary Figure 4: Manhattan and QQplot of the GWAS meta-analysis of childhood maltreatment

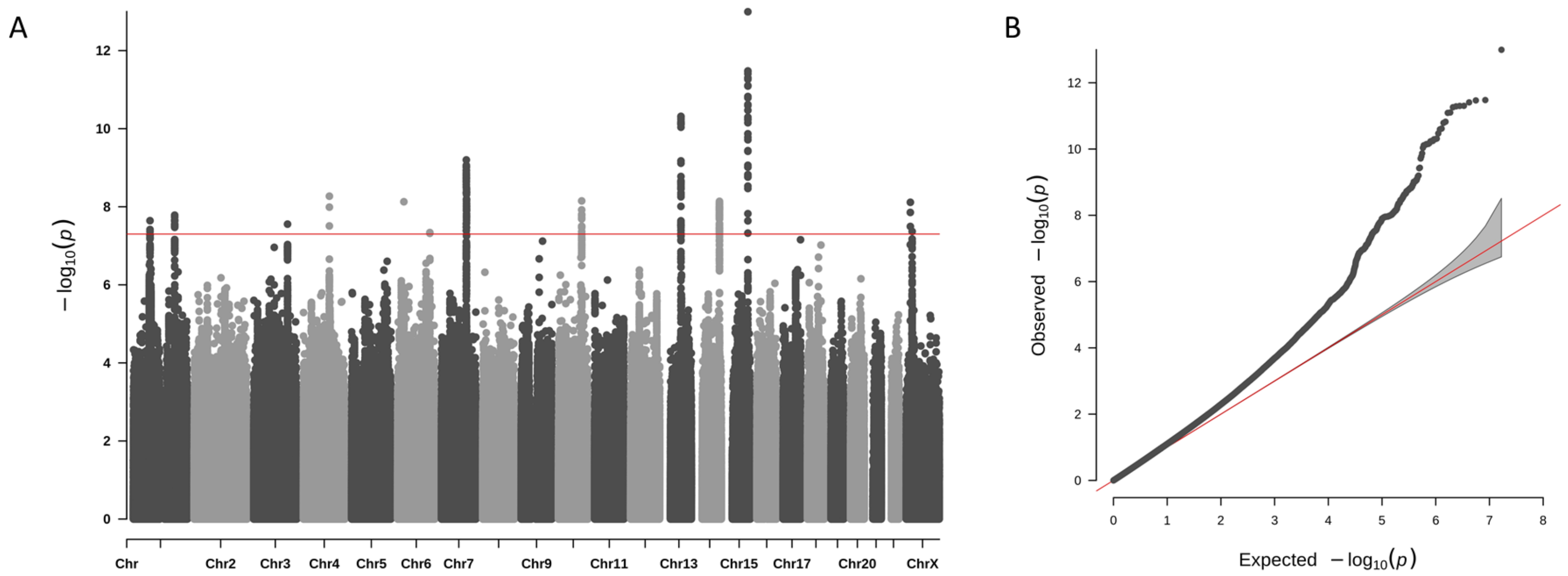

Manhattan plot (A) and quantile-quantile plot (B) of the GWAS meta-analysis of childhood maltreatment.

Supplementary Figure 5: LocusZoom plot for rs12031035

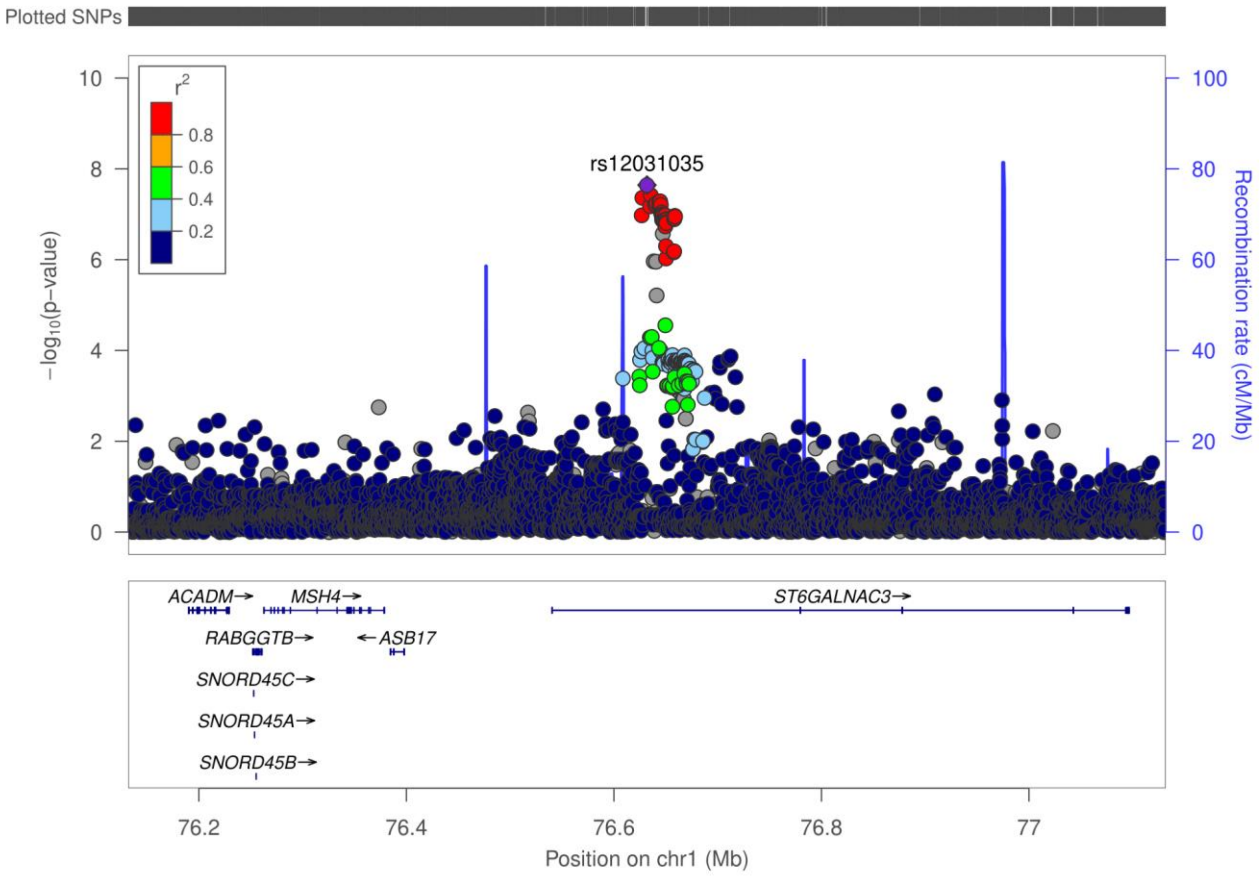

The purple diamond is the most statistically significant SNP (index SNP) in the region, with other SNPs indicated as circles. Colours represent the degree of linkage disequilibrium with the index SNP (key provided in inset) .

Supplementary Figure 6: LocusZoom plot for rs61818983

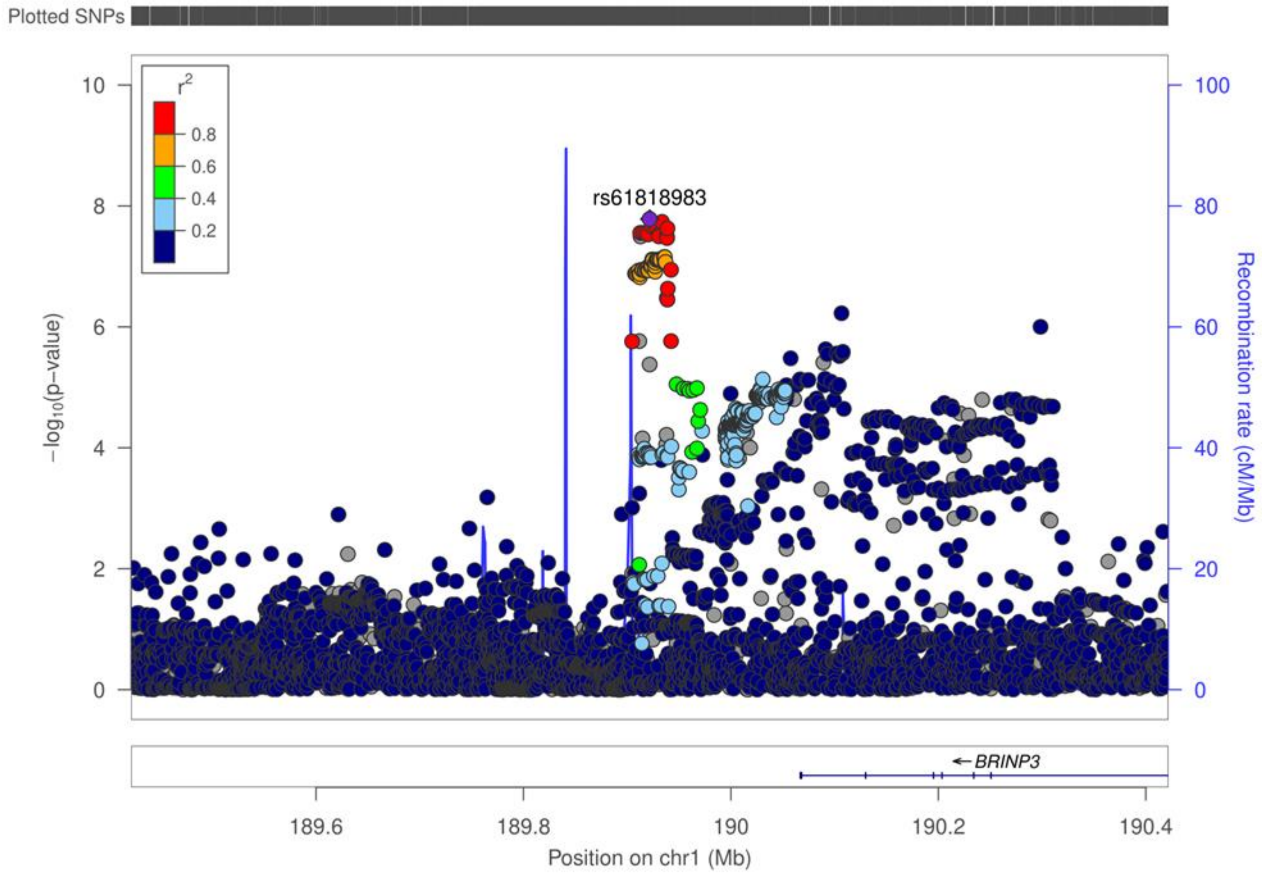

The purple diamond is the most statistically significant SNP (index SNP) in the region, with other SNPs indicated as circles. Colours represent the degree of linkage disequilibrium with the index SNP (key provided in inset) .

Supplementary Figure 7: LocusZoom plot for rs3851357

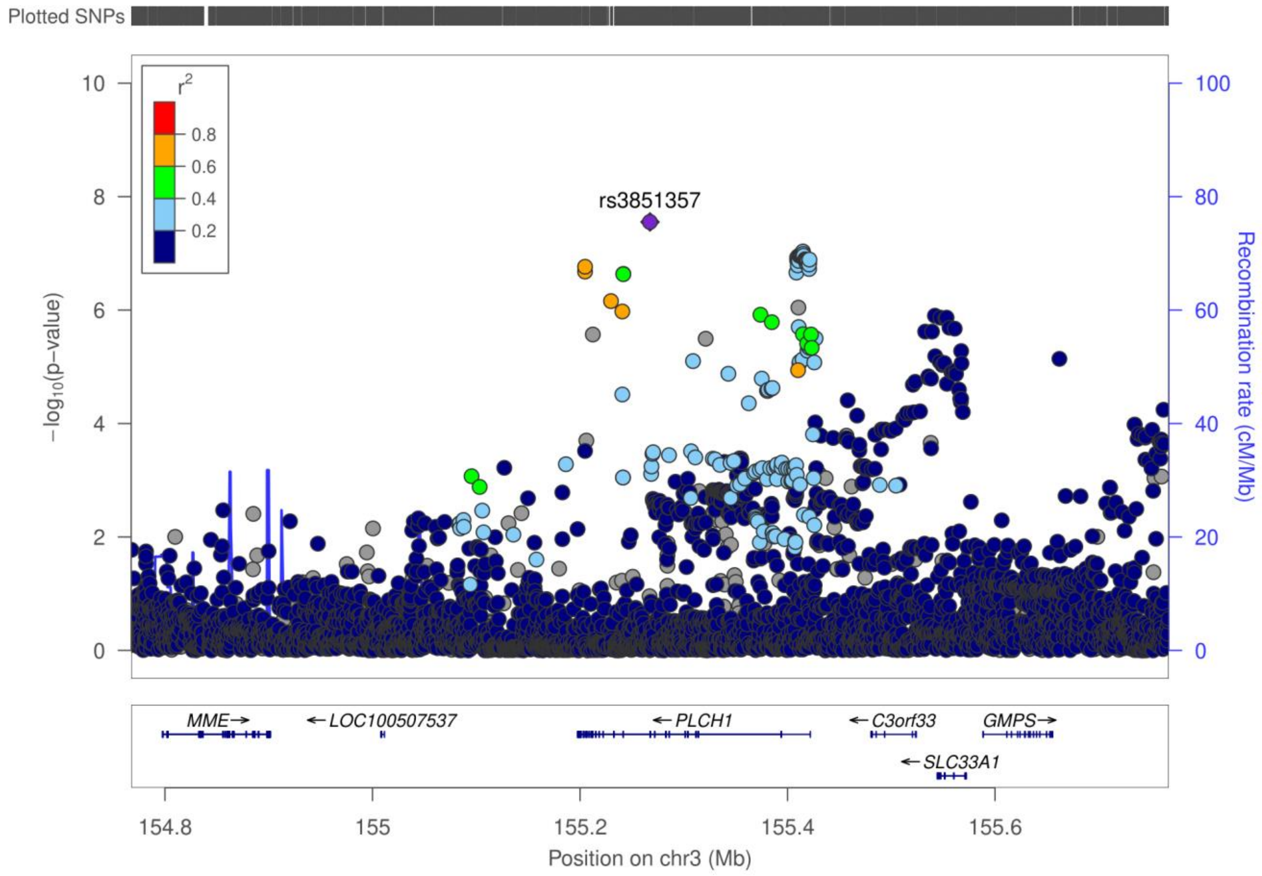

The purple diamond is the most statistically significant SNP (index SNP) in the region, with other SNPs indicated as circles. Colours represent the degree of linkage disequilibrium with the index SNP (key provided in inset).

Supplementary Figure 8: LocusZoom plot for rs611531

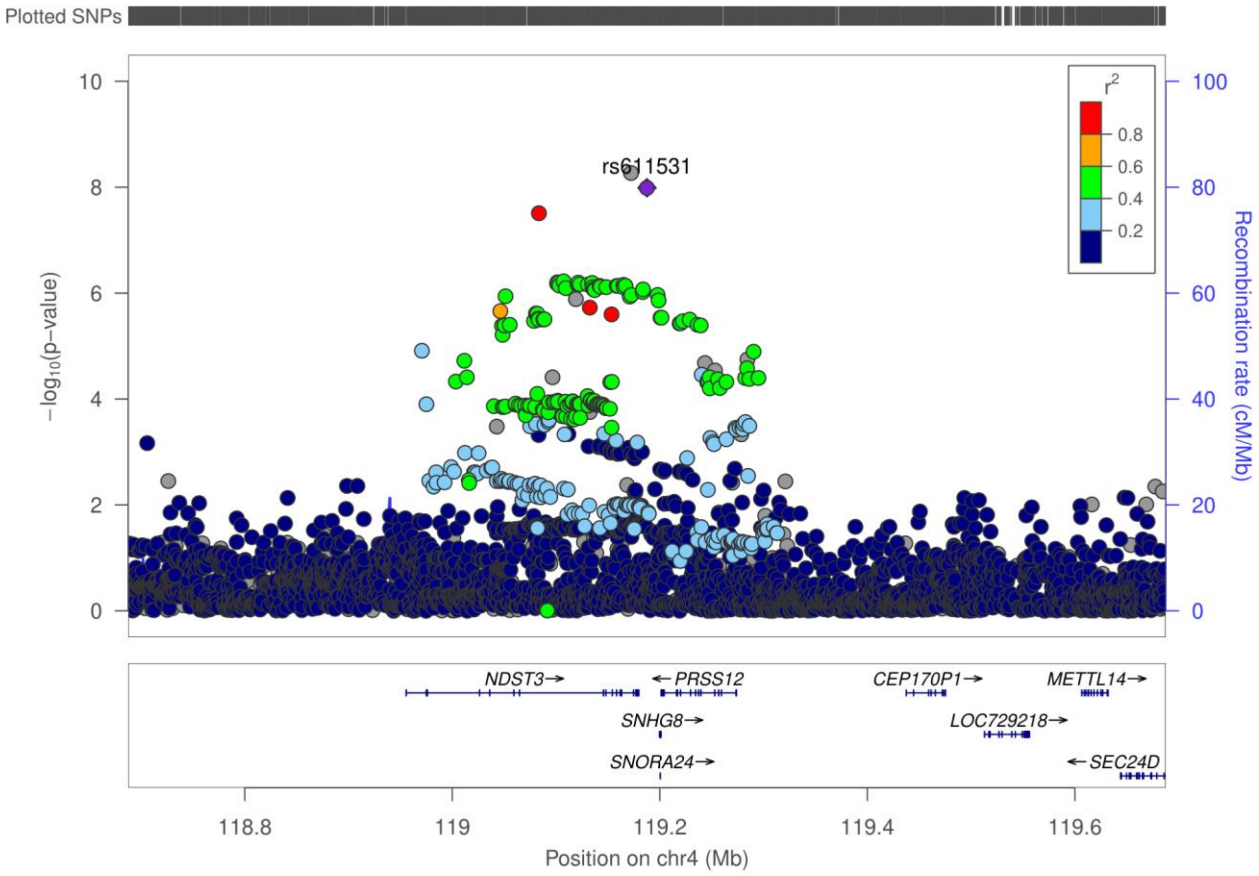

The purple diamond is the most statistically significant SNP (index SNP) in the region, with other SNPs indicated as circles. Colours represent the degree of linkage disequilibrium with the index SNP (key provided in inset).

Supplementary Figure 9: LocusZoom plot for rs4895718

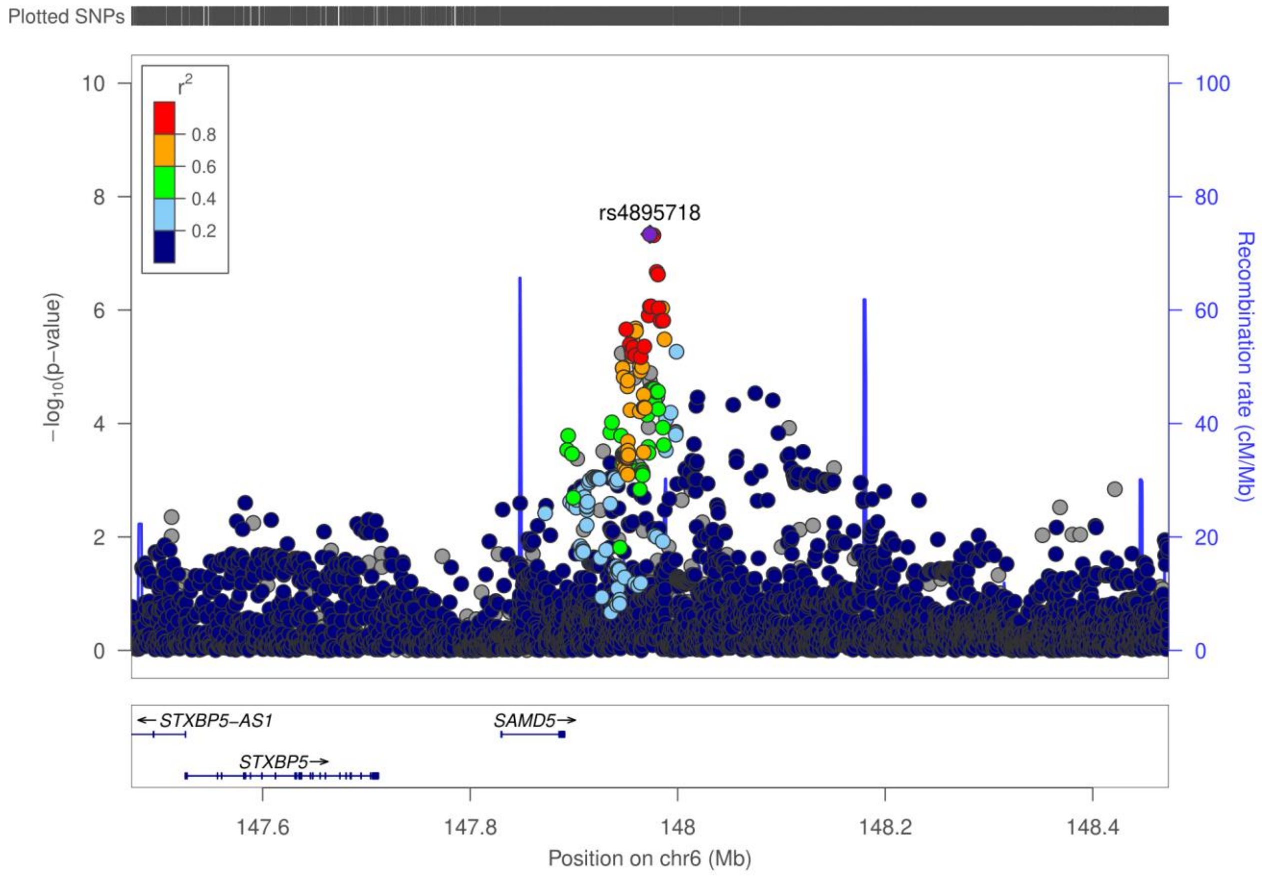

The purple diamond is the most statistically significant SNP (index SNP) in the region, with other SNPs indicated as circles. Colours represent the degree of linkage disequilibrium with the index SNP (key provided in inset).

Supplementary Figure 10: LocusZoom plot for rs7763390

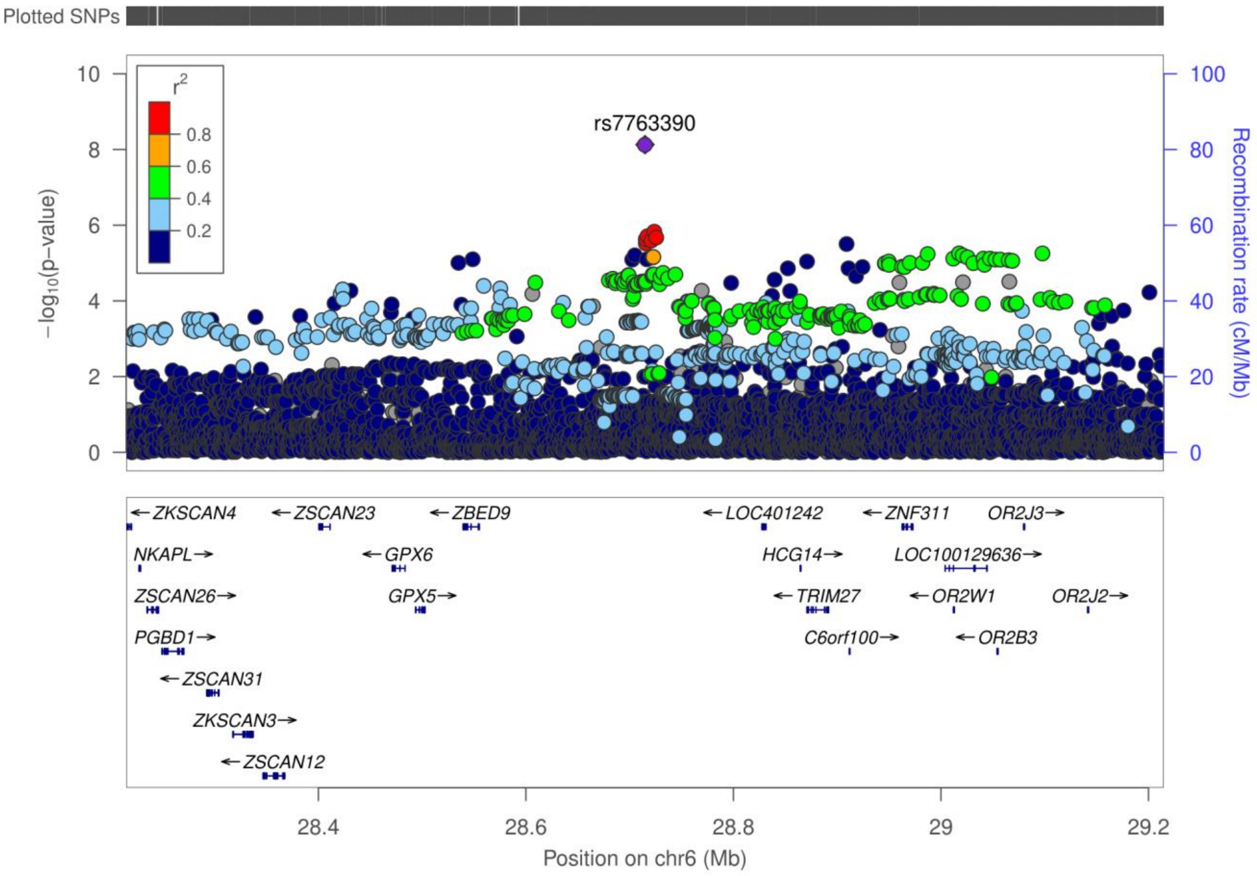

The purple diamond is the most statistically significant SNP (index SNP) in the region, with other SNPs indicated as circles. Colours represent the degree of linkage disequilibrium with the index SNP (key provided in inset).

Supplementary Figure 11: LocusZoom plot for rs1859100

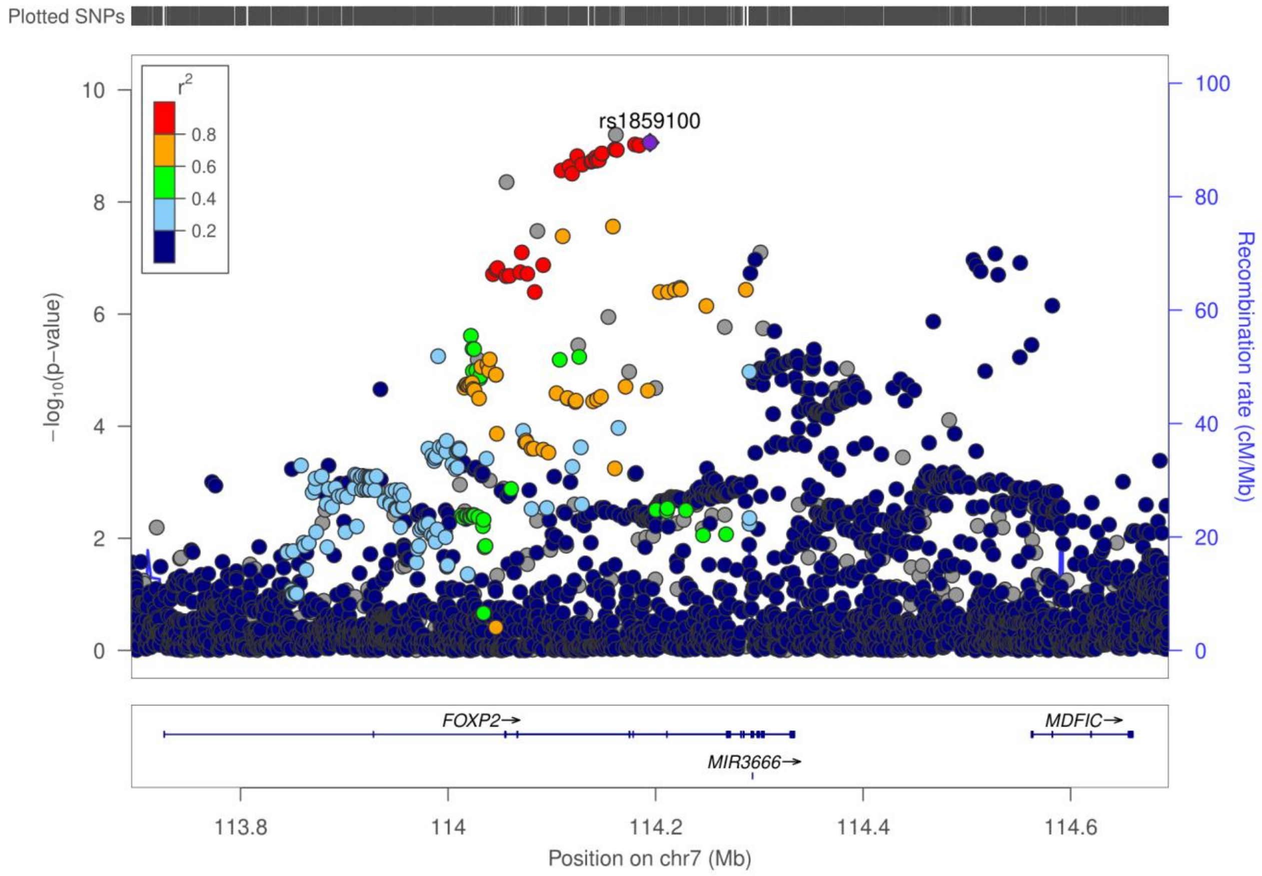

The purple diamond is the most statistically significant SNP (index SNP) in the region, with other SNPs indicated as circles. Colours represent the degree of linkage disequilibrium with the index SNP (key provided in inset).

Supplementary Figure 12: LocusZoom plot for rs4305836

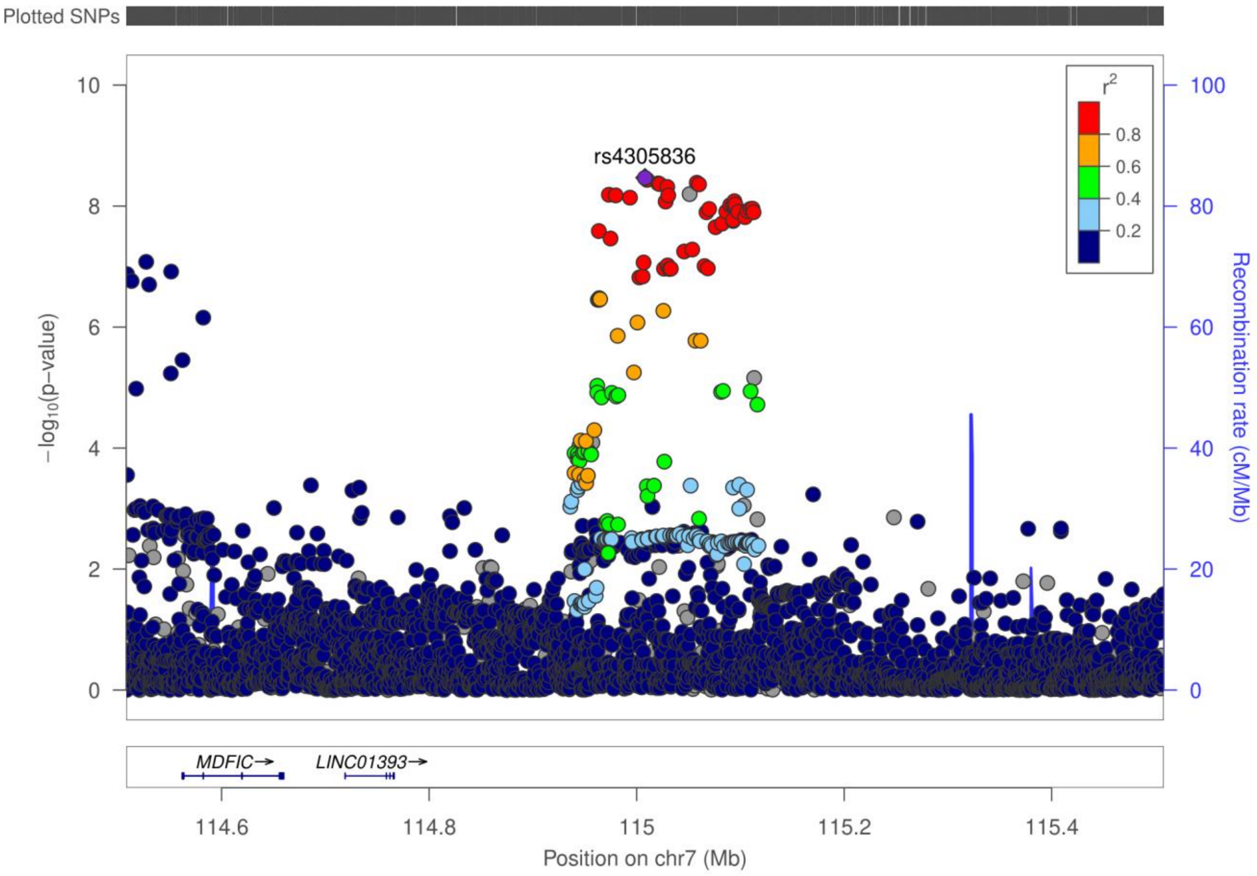

The purple diamond is the most statistically significant SNP (index SNP) in the region, with other SNPs indicated as circles. Colours represent the degree of linkage disequilibrium with the index SNP (key provided in inset).

Supplementary Figure 13: LocusZoom plot for rs3896224

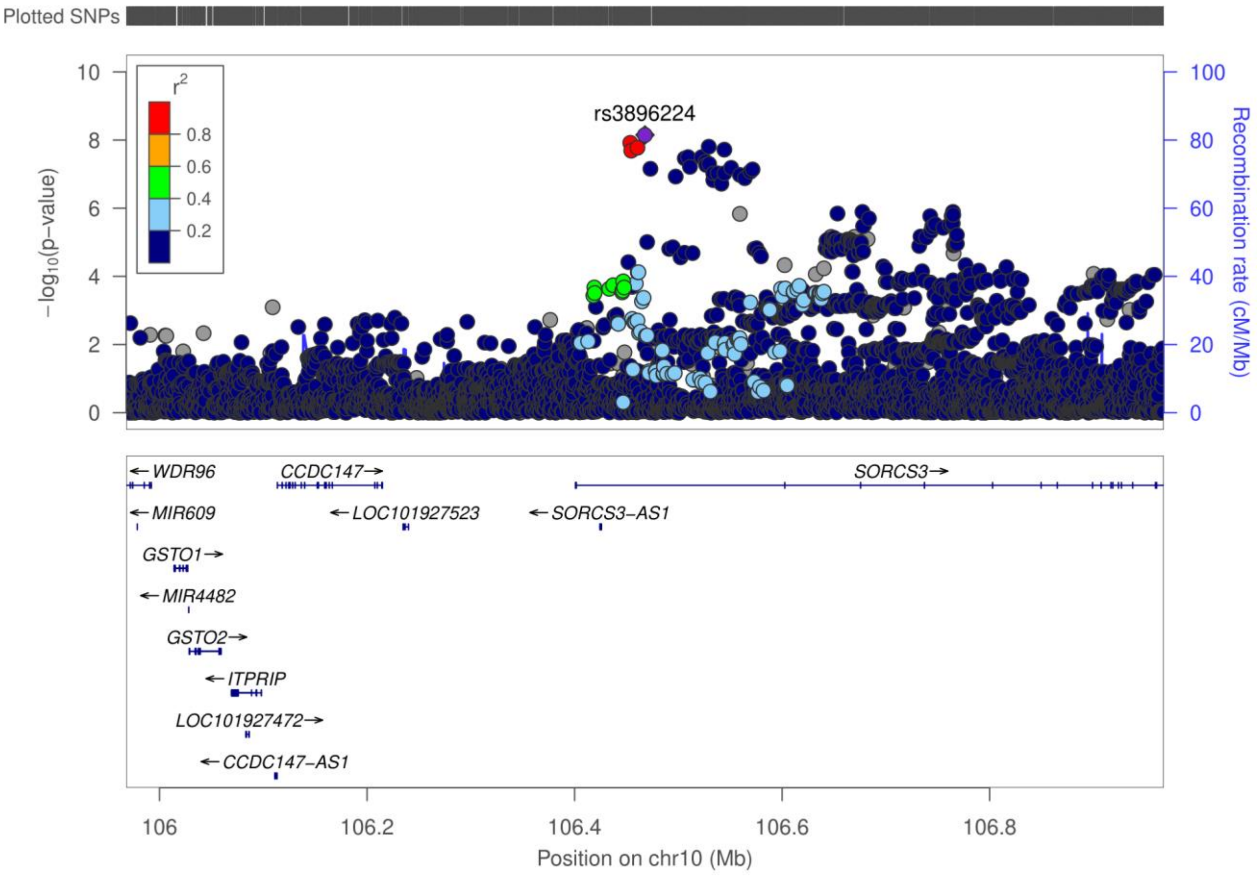

The purple diamond is the most statistically significant SNP (index SNP) in the region, with other SNPs indicated as circles. Colours represent the degree of linkage disequilibrium with the index SNP (key provided in inset).

Supplementary Figure 14: LocusZoom plot for rs35560901

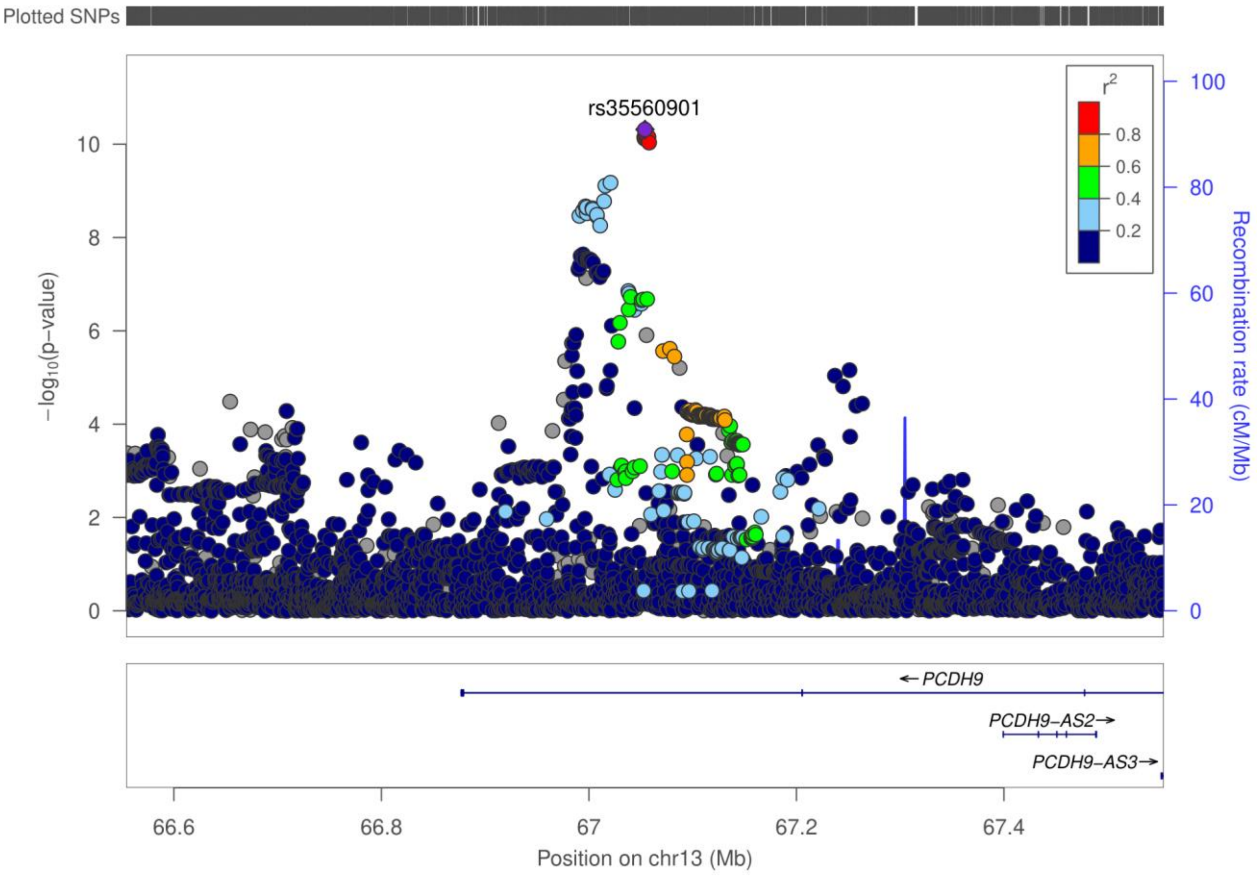

The purple diamond is the most statistically significant SNP (index SNP) in the region, with other SNPs indicated as circles. Colours represent the degree of linkage disequilibrium with the index SNP (key provided in inset).

Supplementary Figure 15: LocusZoom plot for rs77987546

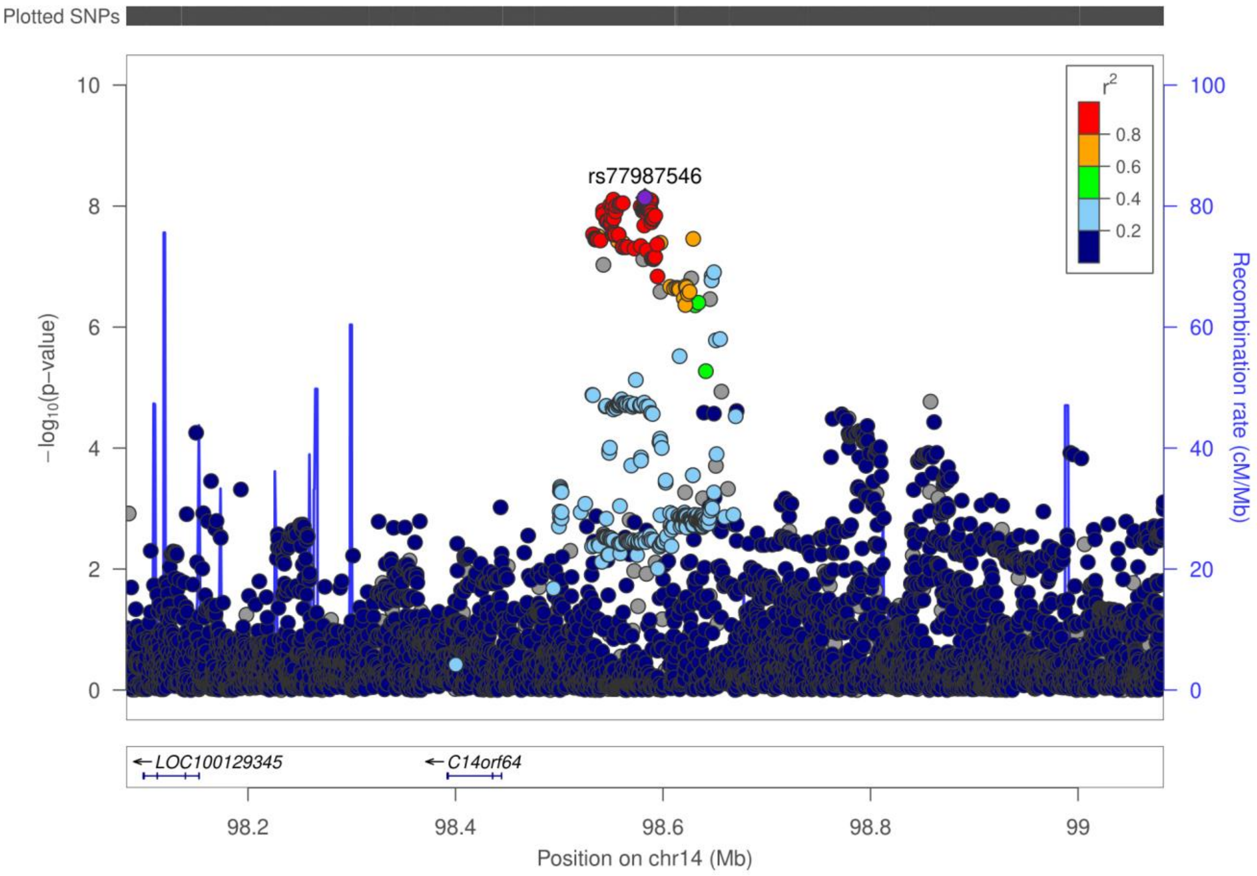

The purple diamond is the most statistically significant SNP (index SNP) in the region, with other SNPs indicated as circles. Colours represent the degree of linkage disequilibrium with the index SNP (key provided in inset).

Supplementary Figure 16: LocusZoom plot for rs4702

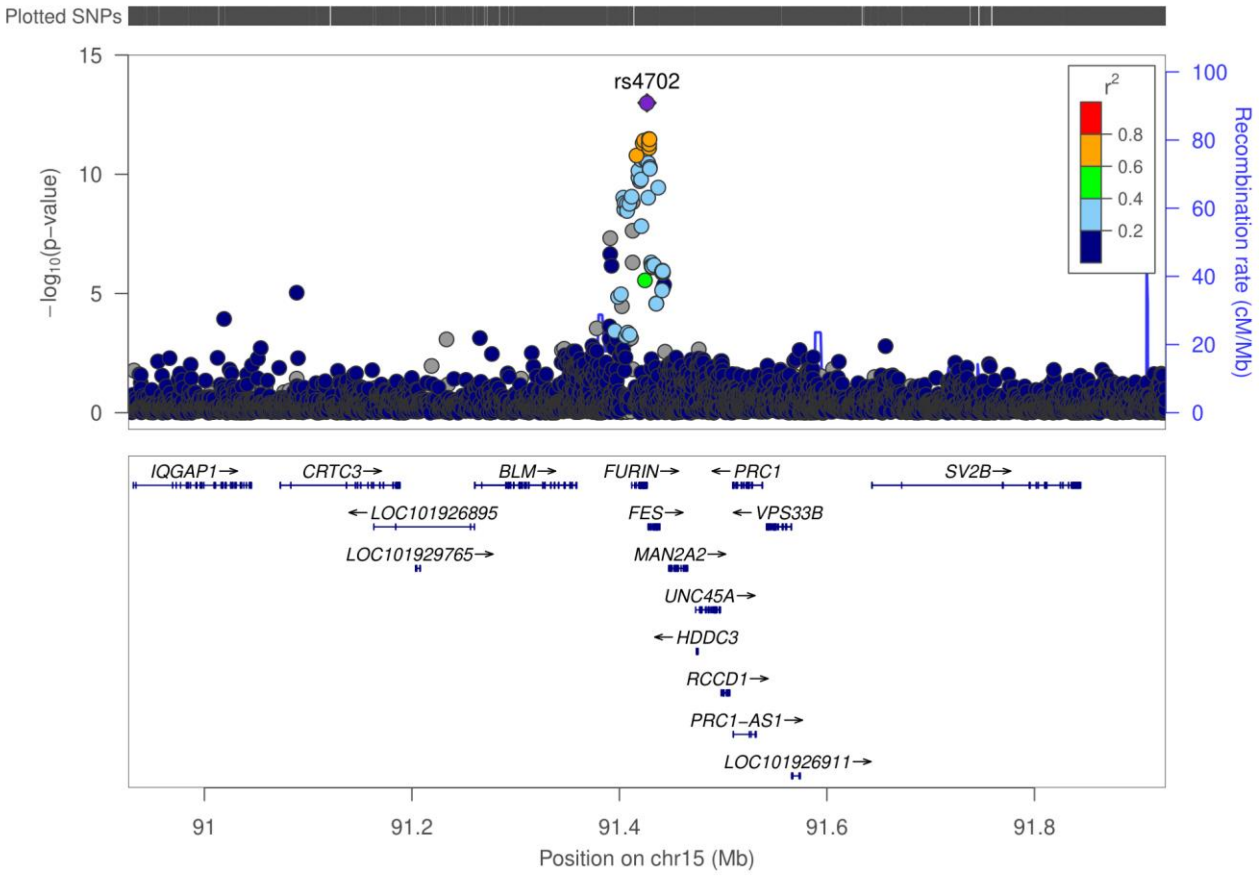

The purple diamond is the most statistically significant SNP (index SNP) in the region, with other SNPs indicated as circles. Colours represent the degree of linkage disequilibrium with the index SNP (key provided in inset).

Supplementary Figure 17: LocusZoom plot for rs5928362

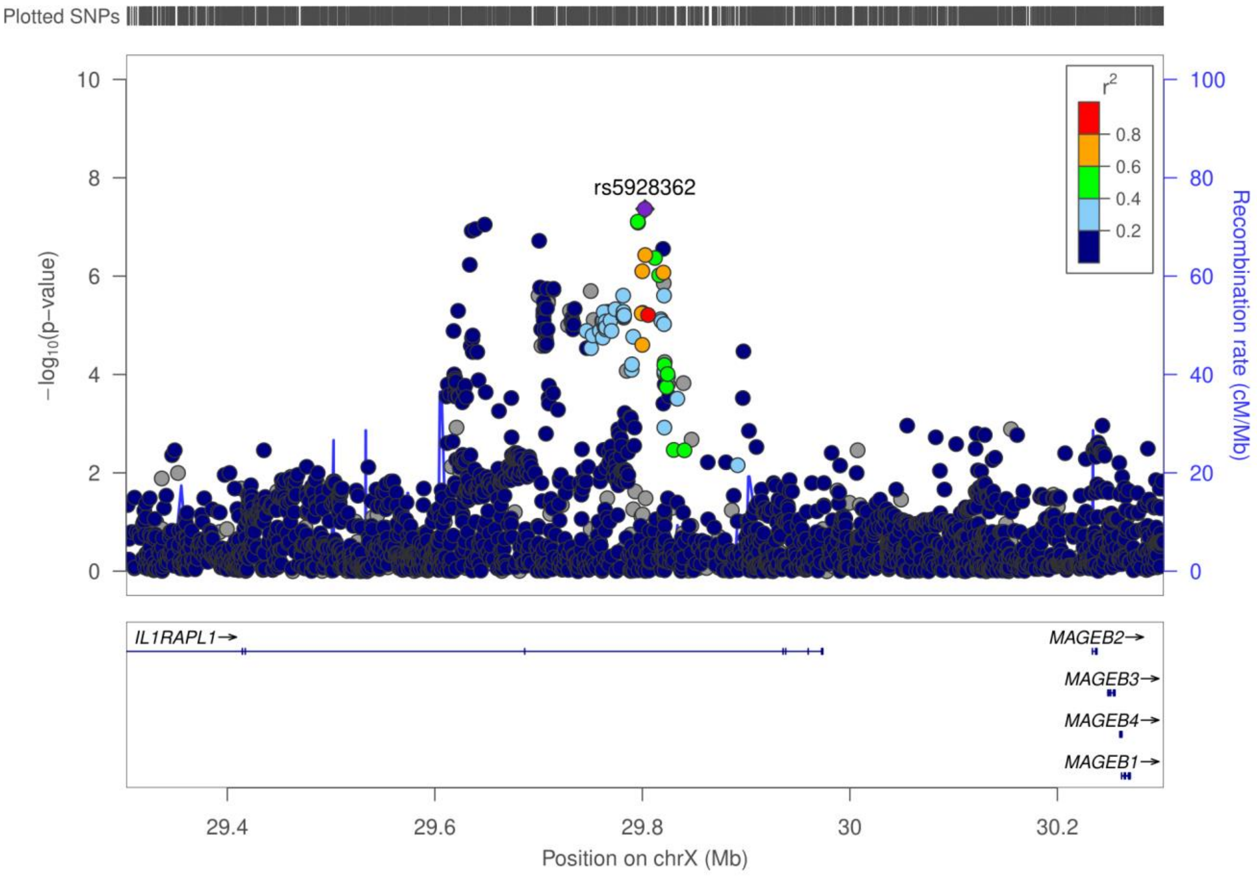

The purple diamond is the most statistically significant SNP (index SNP) in the region, with other SNPs indicated as circles. Colours represent the degree of linkage disequilibrium with the index SNP (key provided in inset).

Supplementary Figure 18: LocusZoom plot for rs6633421

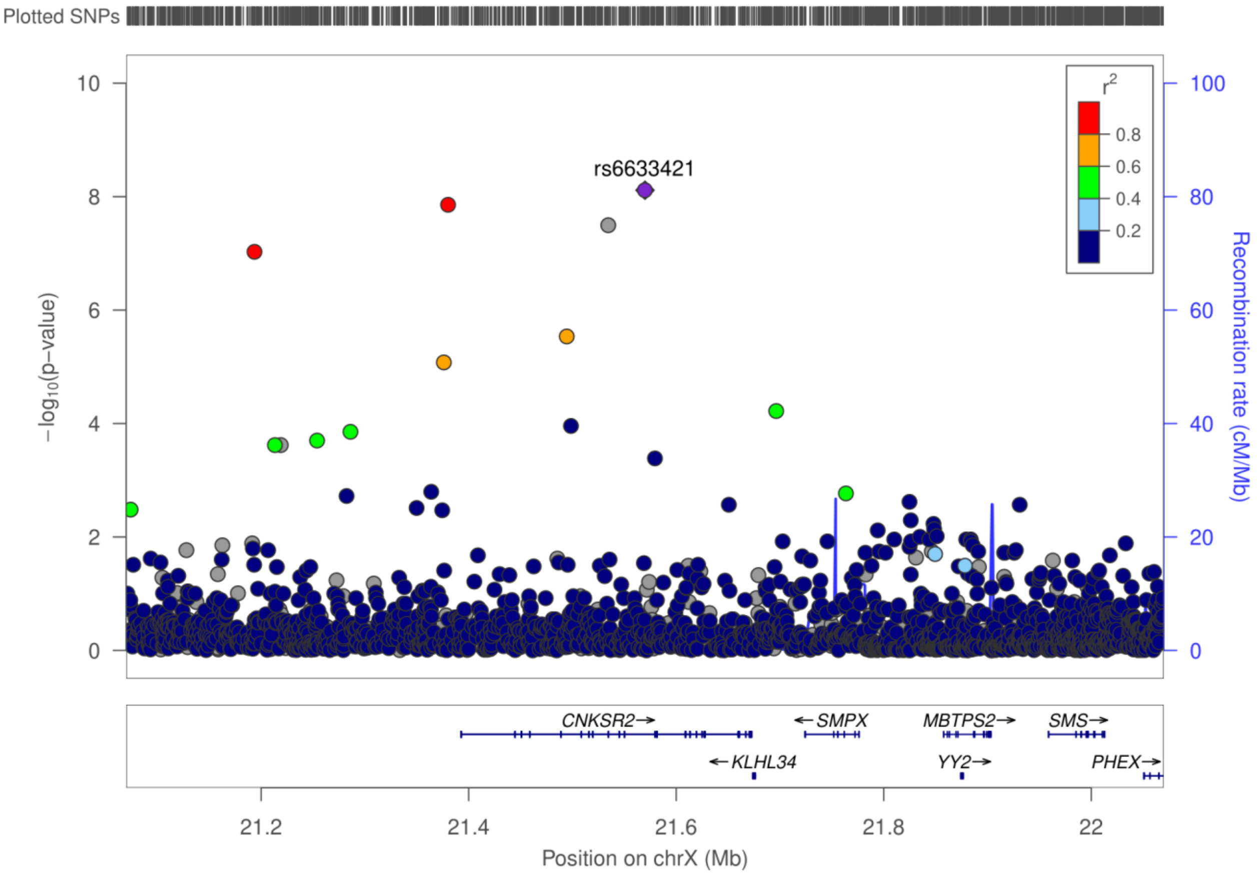

The purple diamond is the most statistically significant SNP (index SNP) in the region, with other SNPs indicated as circles. Colours represent the degree of linkage disequilibrium with the index SNP (key provided in inset).

Supplementary Figure 19: Causal effect of childhood maltreatment on major depressive disorder

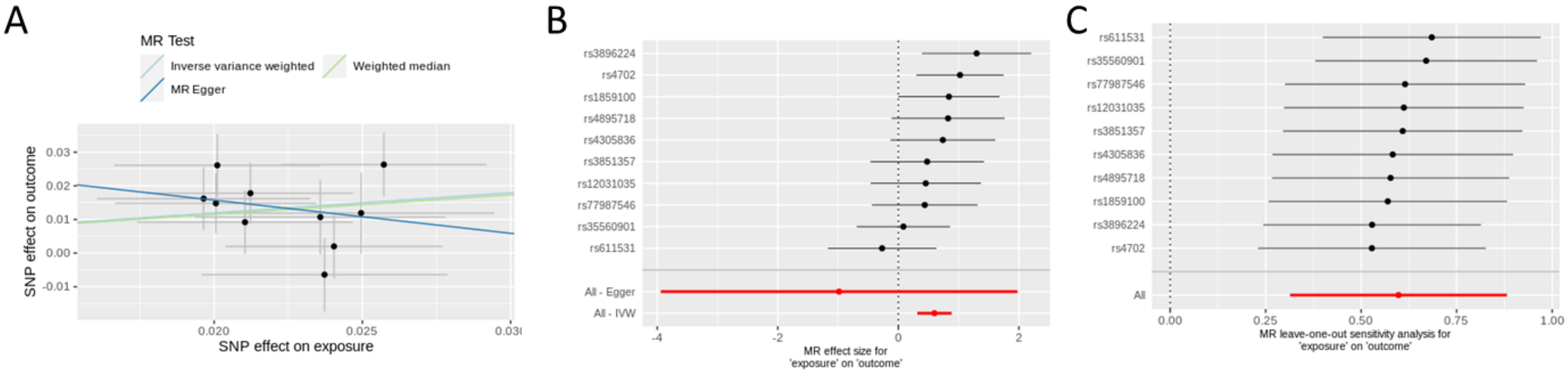

MR-egger intercept: 0.035 (0.033), p = 0.3233

Steiger analyses: snp_r2.exposure = 0.0022, snp_r2.outcome = 0.002, snp_r2.outcome = 0.00019, correct_causal_direction = TRUE, steiger_pval = 1.26e-18

(A) Scatterplot of SNP effects on the exposure and outcome. (B) Forest plot demonstrating the causal effects of each SNPs on their own and using methods that use all SNPs. (C) Plot estimating the effect after excluding each single SNP one-by-one.

Supplementary Figure 20: Causal effect of childhood maltreatment on schizophrenia

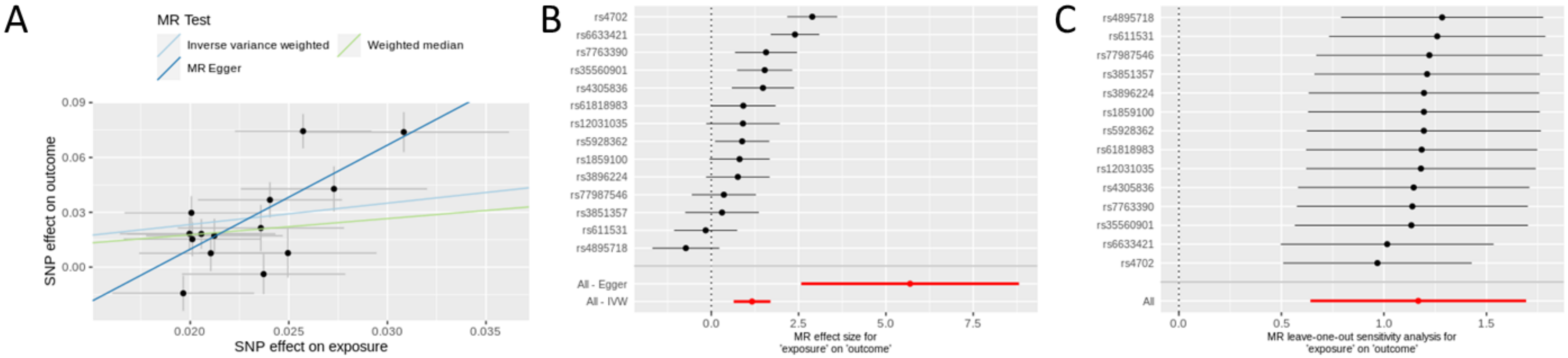

MR-egger intercept: -0.104 (0.036), p = 0.01

Steiger analyses: snp_r2.exposure = 0.0027, snp_r2.outcome = 0.00122, correct_causal_direction = TRUE, steiger_pval = 1.02e-06

(A) Scatterplot of SNP effects on the exposure and outcome. (B) Forest plot demonstrating the causal effects of each SNPs on their own and using methods that use all SNPs. (C) Plot estimating the effect after excluding each single SNP one-by-one.

Supplementary Figure 21: Causal effect of childhood maltreatment on ADHD

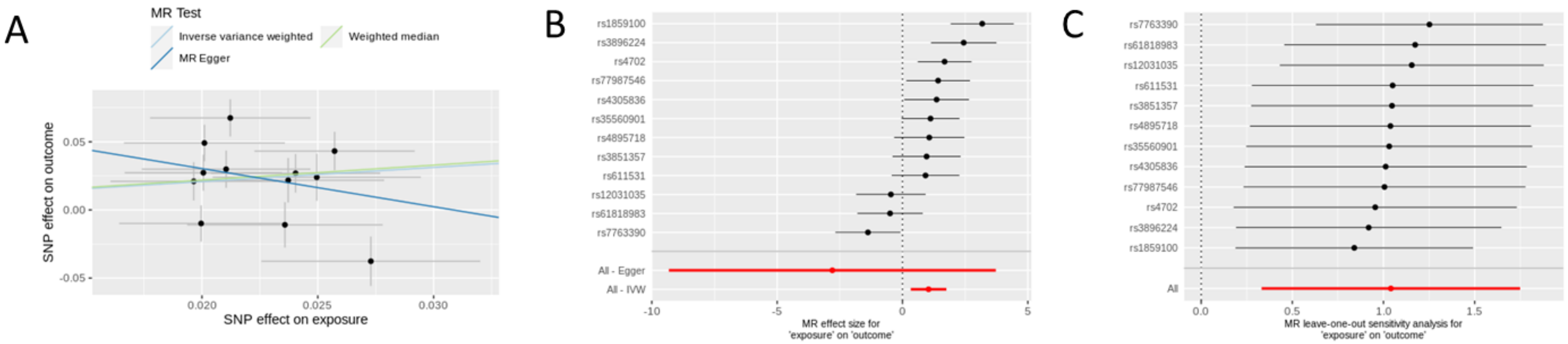

MR-egger intercept: -0.086 (0.074), p = 0.27

Steiger analyses: snp_r2.exposure = 0.0024, snp_r2.outcome = 0.0012, correct_causal_direction = TRUE, steiger_pval = 0.007

(A) Scatterplot of SNP effects on the exposure and outcome. (B) Forest plot demonstrating the causal effects of each SNPs on their own and using methods that use all SNPs. (C) Plot estimating the effect after excluding each single SNP one-by-one.

Supplementary Figure 22: Causal effect of childhood maltreatment on autism

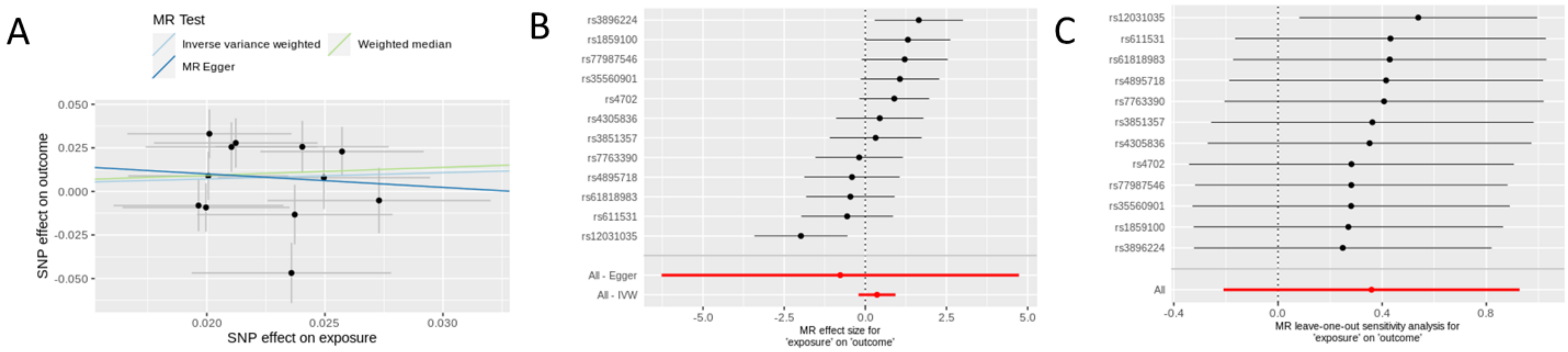

MR-egger intercept: 0.025 (0.062), p = 0.69

Steiger analyses: snp_r2.exposure = 0.0024, snp_r2.outcome = 0.0006, correct_causal_direction = TRUE, steiger_pval = 4.014e-06

(A) Scatterplot of SNP effects on the exposure and outcome. (B) Forest plot demonstrating the causal effects of each SNPs on their own and using methods that use all SNPs. (C) Plot estimating the effect after excluding each single SNP one-by-one.

Supplementary Figure 23: Causal effect of childhood maltreatment on bipolar disorder

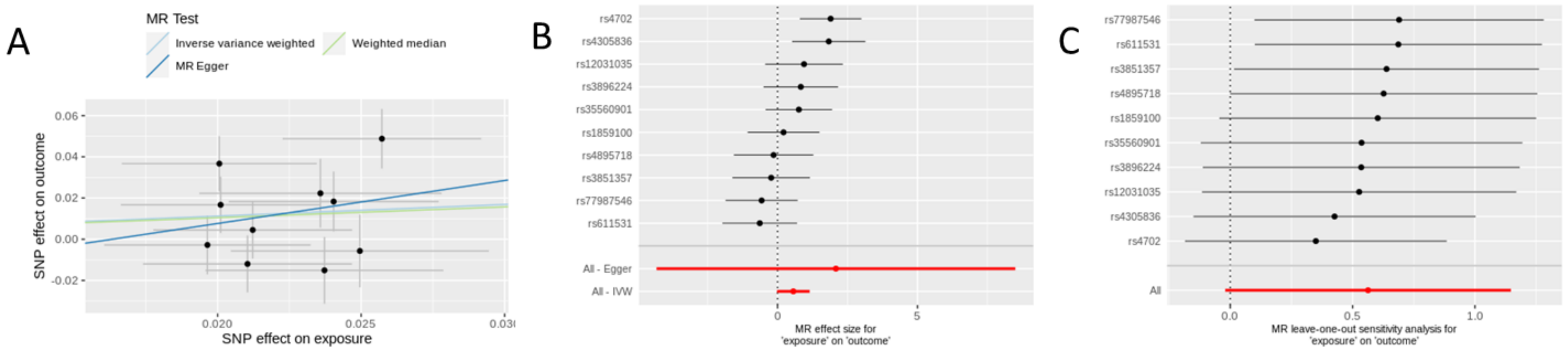

MR-egger intercept: -0.034 (0.072), p = 0.65

Steiger analyses: snp_r2.exposure = 0.002, snp_r2.outcome = 0.0005, correct_causal_direction = TRUE, steiger_pval = 5.624e-06

(A) Scatterplot of SNP effects on the exposure and outcome. (B) Forest plot demonstrating the causal effects of each SNPs on their own and using methods that use all SNPs. (C) Plot estimating the effect after excluding each single SNP one-by-one.

Supplementary Figure 24: Causal effect of childhood maltreatment on C-Reactive Protein

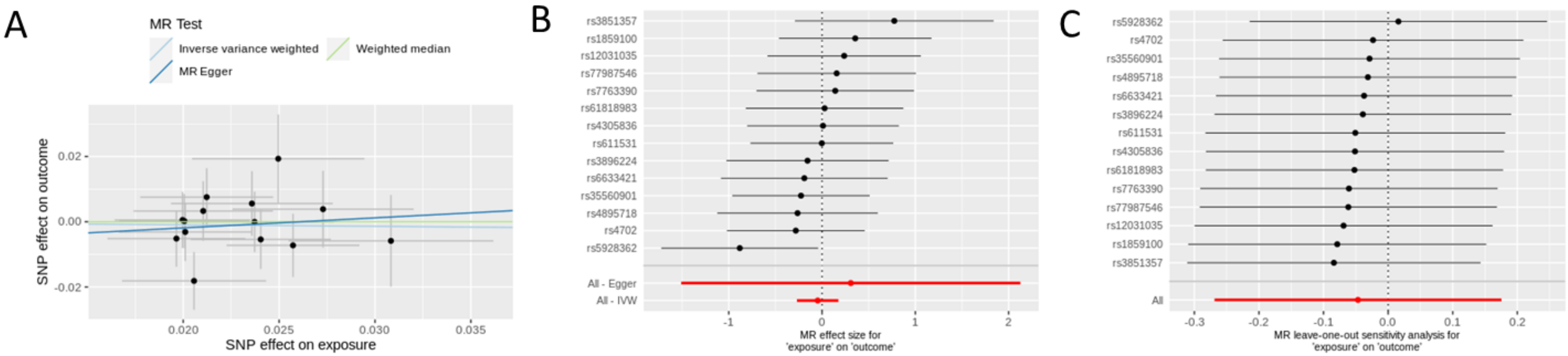

MR-egger intercept: -0.008 (0.02), p = 0.70

Steiger analyses: snp_r2.exposure = 0.0027, snp_r2.outcome = 0.0003, correct_causal_direction = TRUE, steiger_pval = 4.95e-08

(A) Scatterplot of SNP effects on the exposure and outcome. (B) Forest plot demonstrating the causal effects of each SNPs on their own and using methods that use all SNPs. (C) Plot estimating the effect after excluding each single SNP one-by-one.

Supplementary Figure 25: Causal effect of childhood maltreatment on coronary artery disease

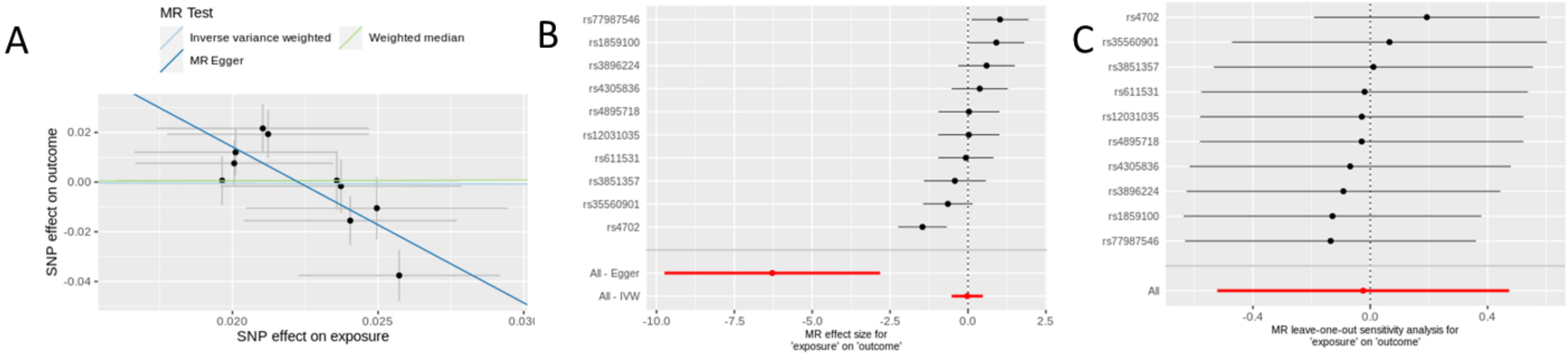

MR-egger intercept: 0.139 (0.039), p = 0.007

Steiger analyses: snp_r2.exposure = 0.002, snp_r2.outcome = 0.0001, correct_causal_direction = TRUE, steiger_pval = 1.63e-23

(A) Scatterplot of SNP effects on the exposure and outcome. (B) Forest plot demonstrating the causal effects of each SNPs on their own and using methods that use all SNPs. (C) Plot estimating the effect after excluding each single SNP one-by-one.

Supplementary Figure 26: Causal effect of childhood maltreatment on Type 2 diabetes

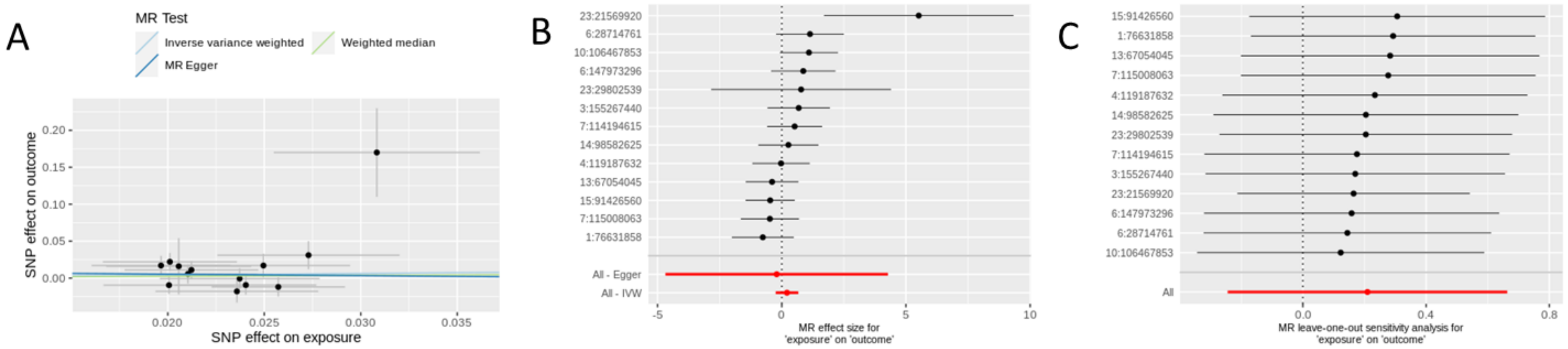

MR-egger intercept: 0.009 (0.05), p = 0.85

Steiger analyses: snp_r2.exposure = 0.0025, snp_r2.outcome = 0.00013, correct_causal_direction = TRUE, steiger_pval = 2.86e-29

(A) Scatterplot of SNP effects on the exposure and outcome. (B) Forest plot demonstrating the causal effects of each SNPs on their own and using methods that use all SNPs. (C) Plot estimating the effect after excluding each single SNP one-by-one.

Supplementary Figure 27: Causal effect of major depressive disorder on childhood maltreatment

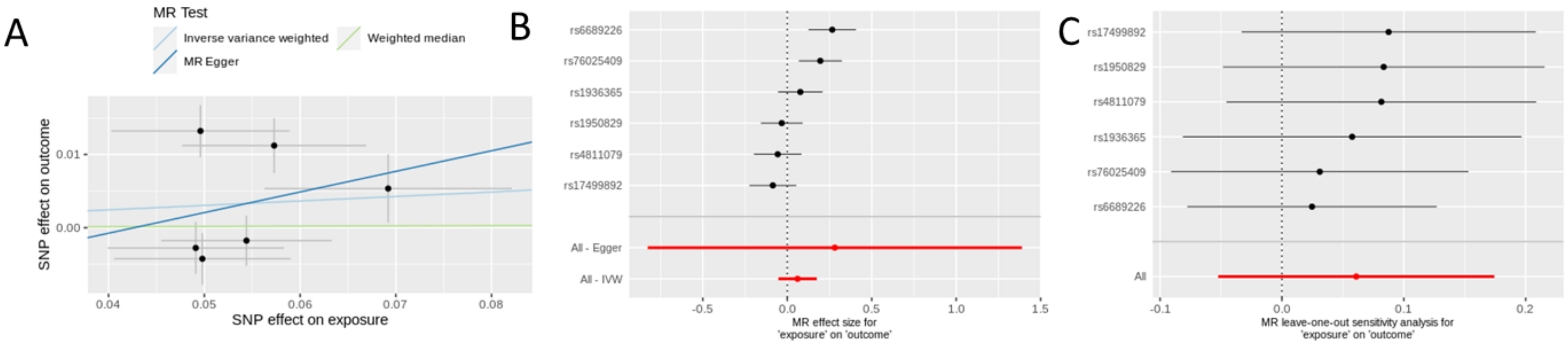

MR-egger intercept: -0.012 (0.03), p = 0.71

Steiger analyses: snp_r2.exposure = 0.0013, snp_r2.outcome = 0.00014, correct_causal_direction = TRUE, steiger_pval = 1.0183e-12

(A) Scatterplot of SNP effects on the exposure and outcome. (B) Forest plot demonstrating the causal effects of each SNPs on their own and using methods that use all SNPs. (C) Plot estimating the effect after excluding each single SNP one-by-one.

Supplementary Figure 28: Causal effect of schizophrenia on childhood maltreatment

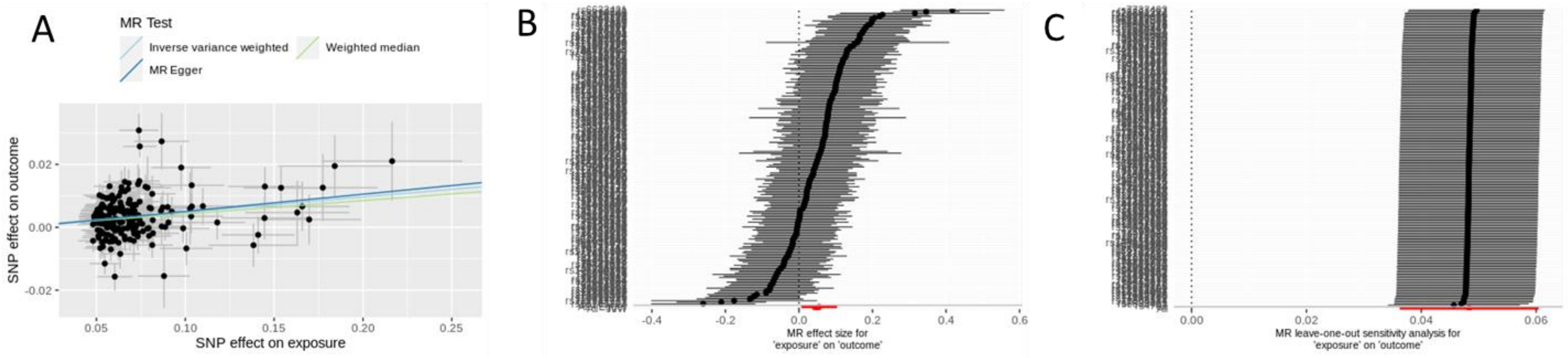

MR-egger intercept: -0.0004 (0.002), p = 0.78

Steiger analyses: snp_r2.exposure = 0.0600, snp_r2.outcome = 0.0027, correct_causal_direction = TRUE, steiger_pval = 0

(A) Scatterplot of SNP effects on the exposure and outcome. (B) Forest plot demonstrating the causal effects of each SNPs on their own and using methods that use all SNPs. (C) Plot estimating the effect after excluding each single SNP one-by-one.

Supplementary Figure 29: Causal effect of ADHD on childhood maltreatment

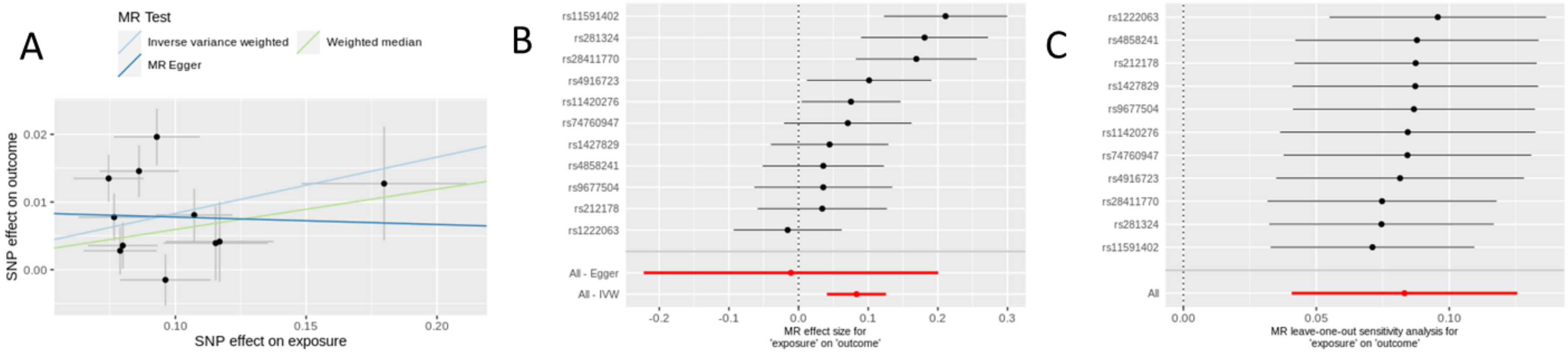

MR-egger intercept: 0.008 (0.009), p = 0.39

Steiger analyses: snp_r2.exposure = 0.007, snp_r2.outcome = 0.0003, correct_causal_direction = TRUE, steiger_pval = 7.796e-42

(A) Scatterplot of SNP effects on the exposure and outcome. (B) Forest plot demonstrating the causal effects of each SNPs on their own and using methods that use all SNPs. (C) Plot estimating the effect after excluding each single SNP one-by-one.

Supplementary Figure 30: Causal effect of autism on childhood maltreatment

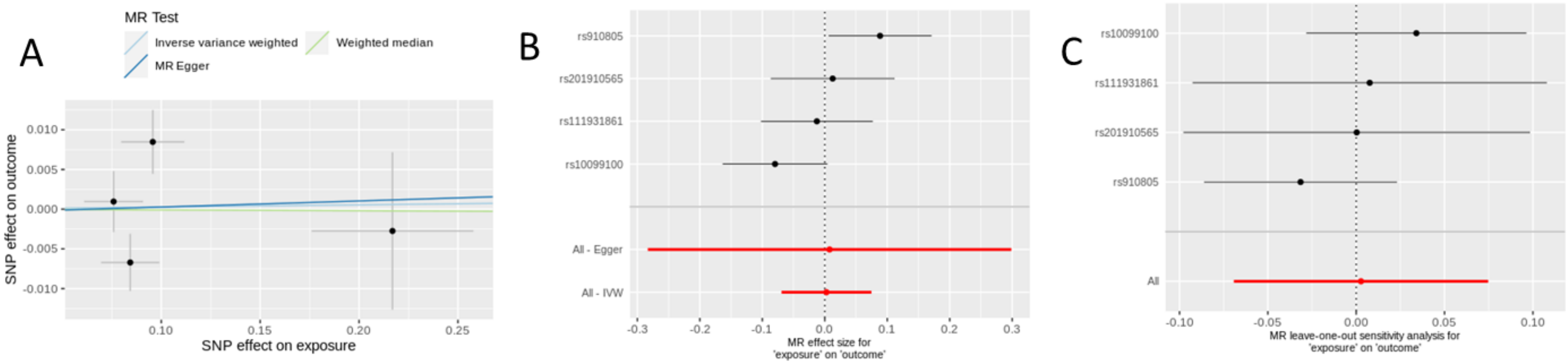

MR-egger intercept: -0.0004 (0.014), p = 0.97

Steiger analyses: snp_r2.exposure = 0.0028, snp_r2.outcome = 4.33e-05, correct_causal_direction = TRUE, steiger_pval = 5.12e-19

(A) Scatterplot of SNP effects on the exposure and outcome. (B) Forest plot demonstrating the causal effects of each SNPs on their own and using methods that use all SNPs. (C) Plot estimating the effect after excluding each single SNP one-by-one.

Supplementary Figure 31: Causal effect of bipolar disorder on childhood maltreatment

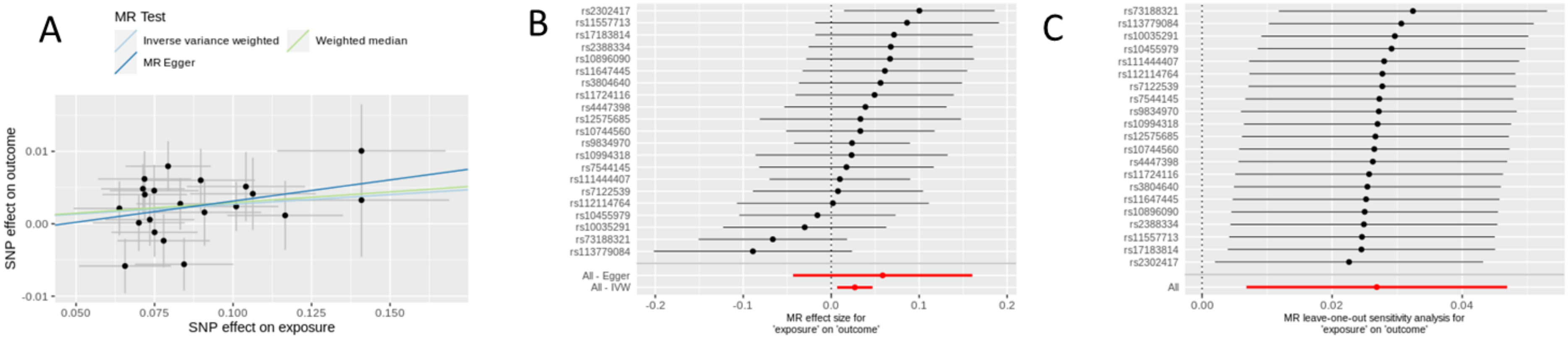

MR-egger intercept: -0.002 (0.004), p = 0.54

Steiger analyses: snp_r2.exposure = 0.0126, snp_r2.outcome = 0.0001, correct_causal_direction = TRUE, steiger_pval = 2.024e-90

(A) Scatterplot of SNP effects on the exposure and outcome. (B) Forest plot demonstrating the causal effects of each SNPs on their own and using methods that use all SNPs. (C) Plot estimating the effect after excluding each single SNP one-by-one.

Supplementary Figure 32: Causal effect of C-Reactive Protein on childhood maltreatment

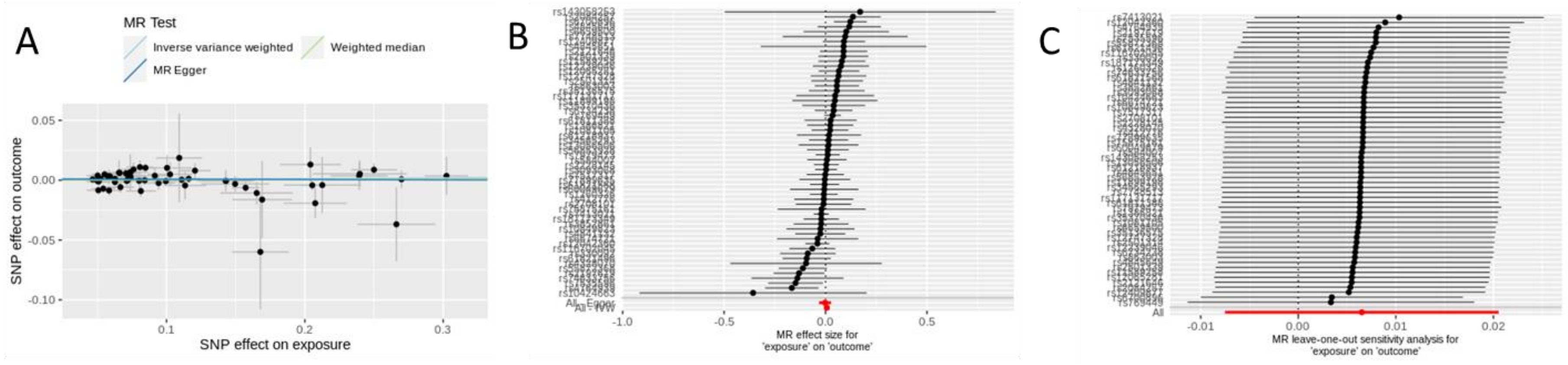

MR-egger intercept: 0.0009 (0.0014), p = 0.50

Steiger analyses: snp_r2.exposure = 0.180, snp_r2.outcome = 0.00036, correct_causal_direction = TRUE, steiger_pval = 0

(A) Scatterplot of SNP effects on the exposure and outcome. (B) Forest plot demonstrating the causal effects of each SNPs on their own and using methods that use all SNPs. (C) Plot estimating the effect after excluding each single SNP one-by-one.

Supplementary Figure 33: Causal effect of coronary artery disease on childhood maltreatment

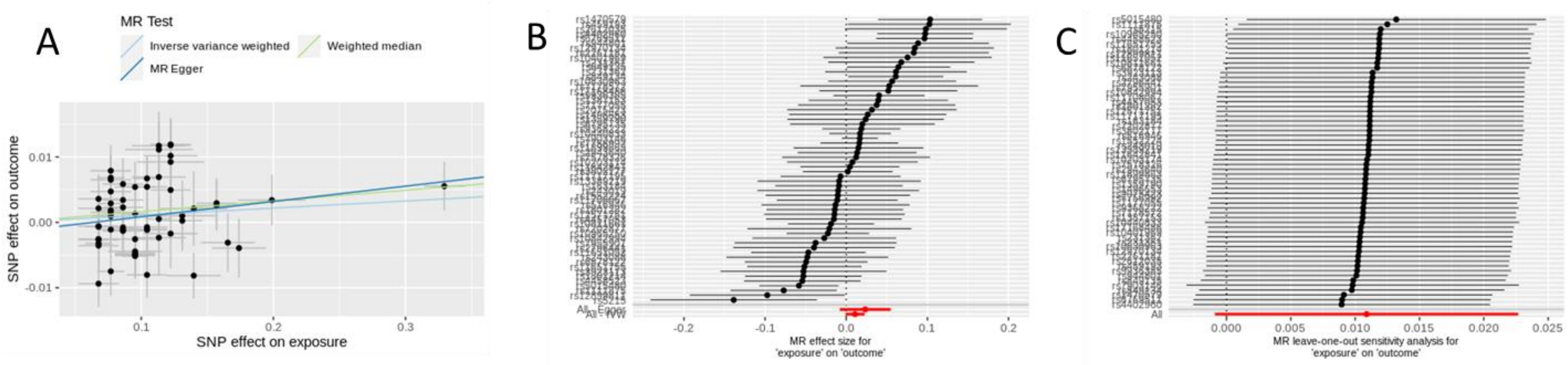

MR-egger intercept: -0.001 (0.0017), p = 0.40

Steiger analyses: snp_r2.exposure = 0.021, snp_r2.outcome = 0.0006, correct_causal_direction = TRUE, steiger_pval = 9.28e-277

(A) Scatterplot of SNP effects on the exposure and outcome. (B) Forest plot demonstrating the causal effects of each SNPs on their own and using methods that use all SNPs. (C) Plot estimating the effect after excluding each single SNP one-by-one.

Supplementary Figure 34: Causal effect of Type 2 diabetes on childhood maltreatment

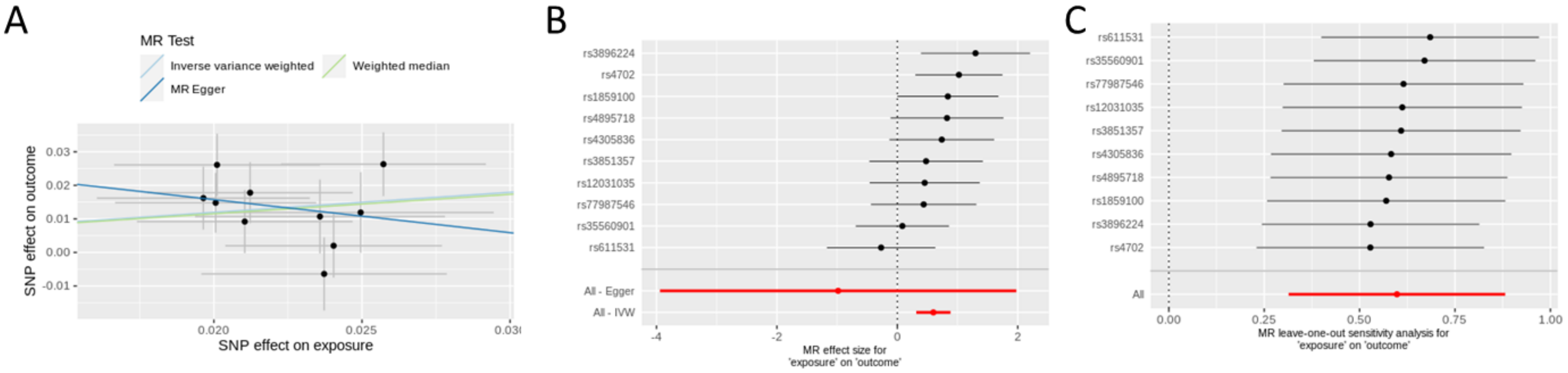

MR-egger intercept: 0.035 (0.033), p = 0.3233

Steiger analyses: snp_r2.exposure = 0.0022, snp_r2.outcome = 0.002, snp_r2.outcome = 0.00019, correct_causal_direction = TRUE, steiger_pval = 1.26e-18

(A) Scatterplot of SNP effects on the exposure and outcome. (B) Forest plot demonstrating the causal effects of each SNPs on their own and using methods that use all SNPs. (C) Plot estimating the effect after excluding each single SNP one-by-one.

Supplementary Table 1: Participant demographics in all cohort

Study number	Study abbreviation	Genotyping array	Childhood maltreatment measure	N	Score range	Score mean (SE)	Sex (% male)	Mean age (SD)	Dataset	Retrospective/Prospective
1	MRSC	OmniExpressExome8 + Custom	Childhood Trauma Questionnaire (CTQ)	2258	0-3	0.43 (0.01)	100%	23.3 (3.2)	PGC_26K	Retrospective
2	ONGA	PsychArray	abbreviated CTQ	212	0-3	0.53 (0.06)	77.30%	33.1 (10.2)	PGC_26K	Retrospective
3	NHS2	PsychArray	CTQ, Conflict Tactics Scale (CTS) , Sexual Experiences Survey	1331	1-3	1.81 (0.02)	0%	35.8 (4.3)	PGC_26K	Retrospective
4	GSDC	Omni1-Quad	Semi-Structured Assessment for Drug Dependence and Alcoholism	1315	0-4	0.53 (0.03)	57.70%	38.2 (10.9)	PGC_26K	Retrospective
5	BRYA	PsychArray	Early life stress questionnaire	315	0-4	0.45 (0.05)	73.70%	47.2 (13.7)	PGC_26K	Retrospective
6	NHRV	PsychArray	Trauma History Screen	1891	0-2	0.26 (0.01)	90.80%	63.7 (13.0)	PGC_26K	Retrospective
7	NSS1	OmniExpressExome8 + Custom	Self-administered questionnaire	7995	0-3	0.48 (0.009)	82.70%	21.0 (3.3)	PGC_26K	Retrospective
8	NSS2	PsychArray	Self-administered questionnaire	2833	0-3	0.67 (0.02)	79.10%	20.3 (3.1)	PGC_26K	Retrospective
9	PPDS	OmniExpressExome8 + Custom	Self-administered questionnaire	7853	0-3	0.28 (0.008)	93.70%	26.0 (6.0)	PGC_26K	Retrospective
10	KSUD	PsychArray 1.1	CTQ	220	0-5	2.21 (0.11)	60%	34.9 (11.0)	PGC_26K	Retrospective
11	BOBA	PsychArray 1.1	Measure created for use in this study.	138	0-5	1.17 (0.09)	35.50%	14.7 (1.7)	PGC_26K	Retrospective
12	GUTS	PsychArray 1.1	Gallup Poll, CTS	515	0-5	0.90 (0.05)	26.60%	26.2 (1.7)	PGC_26K	Retrospective
13	NHSY	PsychArray 1.1	Gallup Poll, CTS	5408	0-6	2.00 (0.02)	0%	51.7 (4.3)	PGC_26K	Retrospective
14	BRY2	PsychArray 1.1	Early life stress questionnaire	121	0-6	2.07 (0.19)	43.10%	41.1 (12.5)	PGC_26K	Retrospective
15	FEEN	PsychArray 1.1	Standardized Trauma Interview	88	0-2	0.73 (0.08)	18.60%	37.3 (12.1)	PGC_26K	Retrospective
16	TEIC	PsychArray 1.1	CTQ	42	0-5	3.05 (0.32)	0%	37.5 (12.9)	PGC_26K	Retrospective

17	NUIT	PsychArray 1.1	Traumatic Life Events Questionnaire and Briere	88	1-3	1.38 (0.07)	0%	19.2 (1.1)	PGC_26K	Retrospective
18	FTCB	PsychArray	CTQ	858	0-3	0.29 (0.02)	95.40%	27.1 (5.9)	PGC_26K	Retrospective
19	UKBB	Affymetrix UK Biobank Axiom array	summary data	143,473	0-20	1.71 (0.006)	43%	63.89 (0.02)	UKB	Retrospective
20	ALSPAC	Illumina HumanHap550	Childhood maltreatment questions	8,346	0-1	0.45(0.004)	49%	NA	ALSPAC	Prospective
21	ABCD	Smokescreen	KSADS-PTSD and CRPBI	5,400	0-13	1.89(0.034)	53%	9.93(0.62)	ABCD	Prospective
22	Generation R	Illumina HumanHap 610 or 660 Quad chips	Stressful life events and trauma interview	1,905	0-2	0.20(0.043)	49%	9.76(0.28)	Generation R	Prospective

Table modified from Dalvie et al., 2020[2]. Key : MRSC- Marine Resiliency Study; ONGA - Ohio National Guard; NHS2 - Nurses Health Study II; GSDC - Yale-Penn Study; BRYA - Ash Wednesday and IVS; NHRV - National Health and Resilience in Veterans Study; NSS1 - Army Study to Assess Risk and Resilience in Service members ; NSS2 - Army Study to Assess Risk and Resilience in Service members ; PPDS - Army Study to Assess Risk and Resilience in Service members ; KSUD - Genetics of Posttraumatic Stress Disorder/Substance Use Disorder Comorbidity ; BOBA - Bounce Back Now; GUTS - Growing Up Today Study; NHSY - Nurses Health Study II; BRY2 – Sydney Neuroimaging; FEEN - OPT and CHOICE; TEIC - McLean Trauma Sample; NUIT - NIU Trauma Orcutt; FTCB - Fort Campbell study; UKBB - UK Biobank; ALSPAC – Avon Longitudinal Study of Parents And Children; ABCD – Adolescent Brain Cognitive Development. ALSPAC measures of childhood maltreatment were derived longitudinally and so have no mean age.

Supplementary Table 2: GCTA-GREML based SNP heritability for operationalizations of childhood maltreatment in the UKBB

	GCTA (GREML)			
Phenotype	h^2_{SNP}	SE	P-value	N
childhood maltreatment (log)	0.093	0.019	6.54E-08	19559
childhood maltreatment (binary)	0.089	0.019	4.10E-07	19559
Severe childhood maltreatment (binary)	0.056	0.018	2.51E-03	19559
Severe childhood abuse (binary)	0.028	0.018	1.06E-01	19559

The table provides the SNP heritability (h^2_{SNP}), associated standard error (SE), p-value, and sample size (N).

Supplementary Table 3: GCTA-GREML based genetic correlations between operationalizations of childhood maltreatment in the UKBB

	childhood maltreatment (log)	childhood maltreatment (binary)	Severe childhood maltreatment (binary)	Severe childhood abuse (binary)
childhood maltreatment (log)	NA	0.95 (0.032)	0.68 (0.11)	0.90 (0.23)
childhood maltreatment (binary)	1.11E-193	NA	0.47 (0.16)	0.60 (0.27)
Severe childhood maltreatment (binary)	6.4E-10	0.003	NA	1 (0.17)
Severe childhood abuse (binary)	9.2E-5	0.02	4.1E-09	NA

The table provides the genetic correlations and associated standard errors in parenthesis between different operationalizations of childhood maltreatment in the UKBB (upper triangle). The associated p-values are provided in the lower triangles. All results were significant after correcting for multiple testing using Benjamini-Hochberg FDR correction.

Supplementary Table 4: GCTA-GREML based SNP heritability for different subtypes of childhood maltreatment in the UKBB

	No deprivation			With deprivation			
	h^2_{SNP}	SE	p-value	h^2_{SNP}	SE	p-value	N
Childhood maltreatment	0.093	0.018	6.54E-08	0.090	0.09	1.53E-07	19559
Sexual abuse (binary)	0.068	0.018	6.76E-05	0.068	0.018	7.78E-05	19559
Emotional abuse (binary)	0.036	0.017	0.016	0.035	0.017	0.01912	19559
Physical abuse (binary)	0.057	0.018	0.0007	0.055	0.018	0.00108	19559
Physical neglect (binary)	0.031	0.018	0.037	0.031	0.017	0.03455	19559
Emotional neglect (binary)	0.052	0.018	0.0014	0.049	0.018	0.00233	19559

The table provides the SNP heritability and associated standard errors and p-values for childhood maltreatment (log-transformed) and subtypes in the UKBB. SNP heritability has also been calculated after including Townsend Deprivation Index as a covariate. All results were significant after correcting for multiple tests using Benjamini-Hochberg FDR correction.

Supplementary Table 5: GCTA-GREML based genetic correlations between subtypes of childhood maltreatment in the UKBB

	Childhood maltreatment	Sexual abuse (binary)	Emotional abuse (binary)	Physical abuse (binary)	Physical neglect (binary)	Emotional neglect (binary)
Childhood maltreatment	NA	0.62 (0.13)	0.98 (0.13)	1.00 (0.11)	0.66 (0.18)	0.91 (0.06)
Sexual abuse (binary)	1.84E-6*	NA	0.68 (0.26)	0.71 (0.21)	0.50 (0.31)	0.24 (0.21)
Emotional abuse (binary)	4.75E-14*	8.91E-3	NA	1.00 (0.22)	0.38 (0.36)	1.00 (0.28)
Physical abuse (binary)	9.82E-20*	7.22E-4*	5.48E-6*	NA	0.67 (0.32)	0.82 (0.22)
Physical neglect (binary)	2.45E-4*	0.10	0.29	0.036*	NA	0.59 (0.27)
Emotional neglect (binary)	5.87E-52*	0.25	3.55E-4*	1.93E-4*	0.028*	NA

The table provides the genetic correlations and associated standard errors in parenthesis between different subtypes of childhood maltreatment in the UKBB. The associated p-values are provided in the lower triangles. *Indicates p-values that survived multiple testing correction after Benjamini-Hochberg FDR correction.

Supplementary Table 6: Lead SNPs from all independent loci with p-value < 1E-6 in the GWAS meta-analysis of retrospectively reported childhood maltreatment

SNP	Chr	Pos	EA	OA	EA freq	INFO	Beta UKBB	SE UKBB	p-value UKBB	Beta PGC_26K	SE PGC_26K	p-value PGC_26K
rs13090329	3	94121904	G	A	0.66	0.96	0.0160	0.003	4.60E-08	0.018	0.016	2.67E-01
rs35077679	4	1.19E+08	A	AT	0.25	0.97	-0.0190	0.003	9.50E-10	0.009	0.018	6.21E-01
rs1015511	7	1.14E+08	A	G	0.41	0.99	-0.0170	0.003	1.90E-10	-0.007	0.011	5.57E-01
rs6954551	7	1.15E+08	G	A	0.85	1.00	0.0200	0.004	4.70E-08	0.020	0.015	2.00E-01
rs192149019	9	98213947	T	C	0.99	0.80	-0.2360	0.042	1.40E-08	NA	NA	NA
rs3843947	13	67020957	G	A	0.47	0.96	-0.0170	0.003	5.00E-10	-0.013	0.014	3.68E-01
rs12436785	14	98550490	T	C	0.59	1.00	-0.0150	0.003	4.40E-08	-0.020	0.013	1.20E-01
rs4702	15	91426560	G	A	0.45	1.00	0.0200	0.003	2.30E-13	0.005	0.014	7.00E-01
rs1378559	23	21380266	T	C	0.86	0.94	0.0180	0.003	1.30E-08	NA	NA	NA
rs5928362	23	29802539	T	A	0.45	0.98	0.0120	0.002	4.20E-08	NA	NA	NA
rs10437537	10	8375765	A	G	0.48	0.99	-0.0131	0.003	9.10E-07	-0.040	0.016	1.20E-02
rs10753937	1	1.90E+08	T	C	0.53	0.97	-0.0136	0.003	4.30E-07	-0.009	0.016	5.91E-01
rs10799923	1	1.63E+08	A	G	0.41	1.00	-0.0135	0.003	6.00E-07	0.001	0.013	9.55E-01
rs11174338	12	40323641	G	T	0.88	0.99	0.0216	0.004	1.10E-07	0.005	0.018	7.95E-01
rs11596241	10	1.07E+08	G	A	0.83	0.99	0.0188	0.004	8.50E-08	0.041	0.017	1.94E-02
rs11990610	8	9964875	C	G	0.71	0.97	-0.0160	0.003	6.50E-08	-0.025	0.017	1.44E-01
rs12031035	1	76631858	A	T	0.20	0.99	0.0166	0.003	5.90E-07	0.033	0.015	2.45E-02
rs12355141	10	1.03E+08	G	T	0.57	0.99	-0.0143	0.003	1.00E-07	0.007	0.015	6.20E-01
rs1350269	14	98542339	G	A	0.59	1.00	-0.0149	0.003	4.40E-08	-0.021	0.013	1.15E-01
rs2043596	16	60605875	A	C	0.67	0.99	0.0145	0.003	2.90E-07	0.004	0.014	7.69E-01
rs2209151	6	1.31E+08	T	G	0.76	0.99	0.0156	0.003	6.40E-07	0.007	0.014	6.11E-01
rs447751	3	1.55E+08	A	G	0.68	1.00	0.0142	0.003	6.10E-07	0.007	0.013	5.81E-01
rs4571923	1	73736562	G	A	0.53	0.99	-0.0140	0.003	1.60E-07	-0.006	0.013	6.43E-01
rs611531	4	1.19E+08	A	G	0.22	1.00	-0.0175	0.003	4.60E-08	-0.007	0.016	6.47E-01

rs62474713	7	1.15E+08	G	A	0.50	0.99	0.0143	0.003	9.40E-08	0.029	0.012	1.52E-02
rs6659411	1	80864062	A	T	0.64	0.96	-0.0148	0.003	1.50E-07	0.002	0.017	9.04E-01
rs6925748	6	50930041	A	G	0.58	1.00	-0.0132	0.003	8.70E-07	-0.015	0.013	2.55E-01
rs7714147	5	1.63E+08	G	A	0.63	1.00	-0.0148	0.003	7.70E-08	0.007	0.014	6.01E-01
rs7763390	6	28714761	A	C	0.84	1.00	-0.0193	0.004	1.40E-07	-0.011	0.014	4.13E-01

The table provides the lead SNPs with p < 1E-06 in the GWAS meta-analysis of retrospectively reported childhood maltreatment. We provide the chromosome (CHR), position (Pos), effect allele (EA), non-effect allele (OA), effect allele frequency (EA freq), imputation r^2 (INFO) in the UKBB, regression beta (Beta) and associated standard error (SE) and p-value for the UKBB and the PGC_26K.

Supplementary Table 7: Variance explained by PGS for retrospective childhood maltreatment in a hold-out sample in the UKBB

P-value threshold	Beta	SE	p-value	R^2 (%)
1	0.066	0.007	< 2E-16	0.912
0.75	0.067	0.007	< 2E-16	0.900
0.5	0.067	0.007	< 2E-16	0.895
0.25	0.066	0.007	< 2E-16	0.879
0.1	0.065	0.007	< 2E-16	0.832
0.01	0.049	0.007	4.22-E12	0.480
0.001	0.036	0.007	5.21E-07	0.252
0.0001	0.030	0.007	3.00E-05	0.174
0.00001	0.018	0.007	7.08E-03	0.063
0.000001	0.017	0.007	1.43E-02	0.060
5.00E-08	0.020	0.007	4.31E-03	0.082

The table provides the percentage of variance explained (R^2 (%)) by PGS for retrospectively reported childhood maltreatment in a hold-out sample of the UKBB (N = 9,924) at 11 different p-value thresholds. Also provided are the regression beta, and the associated standard error and p-value for PGS constructed at each p-value threshold. All p-values were statistically significant after correcting for multiple testing using Benjamini-Hochberg FDR correction (FDR < 0.05).

Supplementary Table 8: Variance explained by PGS for retrospective childhood maltreatment in ALSPAC

Childhood maltreatment PRS (PT=1)						
Age	OR (95% CIs)	SE	P	P_adjusted	R2	N
0-5 years	1.13 (1.06, 1.19)	0.03	0.00005	0.0001385	0.23%	7424
5-10 years	1.12 (1.07, 1.18)	0.03	7.59E-06	3.42E-05	0.23%	6881
11-17 years	1.10 (1.05, 1.17)	0.03	0.0004	0.0006545	0.16%	6303
0-17 years	1.13 (1.08, 1.18)	0.03	2.04E-07	2.45E-06	0.26%	7453
Adjusted for parental depression in the first trimester						
Age	OR (95% CIs)	SE	P	P_adjusted	R^2	N
0-5 years	1.12 (1.04, 1.20)	0.04	0.002	0.0021176	0.19%	5058
5-10 years	1.13 (1.06, 1.20)	0.03	6.00E-05	0.0001543	0.26%	4830
11-17 years	1.10 (1.03, 1.17)	0.04	0.003	0.003	0.16%	4508
0-17 years	1.15 (1.08, 1.21)	0.03	1.32E-06	1.19E-05	0.34%	5060
Adjusted for parental depressive symptoms in the second trimester						
Age	OR (95% CIs)	SE	P	P_adjusted	R^2	N
0-5 years	1.14 (1.06, 1.22)	0.04	0.00019	0.0004235	0.27%	5320
5-10 years	1.11 (1.05, 1.18)	0.03	0.0004	0.0006545	0.19%	5034
11-17 years	1.11 (1.04, 1.18)	0.04	0.002	0.0021176	0.18%	4688
0-17 years	1.14 (1.08, 1.20)	0.03	2.13E-06	1.53E-05	0.31%	5320
Adjusted for maternal depressive symptoms (third trimester) and paternal depressive symptoms (second trimester)						
Age	OR (95% CIs)	SE	P	P_adjusted	R^2	N
0-5 years	1.13 (1.06, 1.21)	0.04	0.0004	0.0006545	0.24%	5398
5-10 years	1.11 (1.05, 1.18)	0.03	0.0003	6.00E-04	0.20%	5134
11-17 years	1.10 (1.04, 1.17)	0.03	0.002	0.0021176	0.16%	4781
0-17 years	1.14 (1.08, 1.20)	0.03	3.31E-06	1.70E-05	0.29%	5398
Adjusted for parental alcohol consumption in the first trimester						
Age	OR (95% CIs)	SE	P	P_adjusted	R^2	N
0-5 years	1.15 (1.07, 1.22)	0.04	0.00007	0.000168	0.28%	5627
5-10 years	1.12 (1.05, 1.18)	0.03	0.0002	0.0004235	0.21%	5313
11-17 years	1.10 (1.04, 1.17)	0.03	0.002	0.0021176	0.16%	4936
0-17 years	1.14 (1.08, 1.20)	0.03	2.63E-06	1.58E-05	0.29%	5627
Adjusted for parental alcohol consumption in the third trimester						
Age	OR (95% CIs)	SE	P	P_adjusted	R^2	N
0-5 years	1.13 (1.05, 1.21)	0.04	0.001	0.00144	0.23%	5075
5-10 years	1.14 (1.07, 1.20)	0.03	0.00001	3.60E-05	0.30%	4860
11-17 years	1.10 (1.03, 1.17)	0.04	0.003	0.003	0.16%	4546
0-17 years	1.17 (1.10, 1.23)	0.03	4.08E-08	1.47E-06	0.43%	5075
Adjusted for parental smoking in the second trimester						

Age	OR (95% CIs)	SE	P	P_adjusted	R^2	N
0-5 years	1.13 (1.06, 1.21)	0.04	0.0004	0.0006545	0.24%	5468
5-10 years	1.11 (1.05, 1.18)	0.03	0.0005	0.0007826	0.18%	5151
11-17 years	1.10 (1.04, 1.18)	0.04	0.002	0.0021176	0.17%	4790
0-17 years	1.13 (1.07, 1.19)	0.03	9.57E-06	3.60E-05	0.26%	5468
Adjusted for parental smoking in the third trimester						
Age	OR (95% CIs)	SE	P	P_adjusted	R^2	N
0-5 years	1.12 (1.04, 1.19)	0.04	0.002	0.0021176	0.19%	5117
5-10 years	1.13 (1.07, 1.20)	0.03	0.00004	0.00012	0.27%	4904
11-17 years	1.11 (1.04, 1.18)	0.04	0.002	0.0021176	0.18%	4582
0-17 years	1.16 (1.10, 1.22)	0.03	1.19E-07	2.14E-06	0.40%	5117
Adjusted for maternal and paternal history of childhood trauma (assessed across pregnancy)						
Age	OR (95% CIs)	SE	P	P_adjusted	R^2	N
0-5 years	1.11 (1.04, 1.18)	0.04	0.002	0.0021176	0.16%	5987
5-10 years	1.10 (1.04, 1.16)	0.03	0.001	0.00144	0.16%	5656
11-17 years	1.10 (1.04, 1.17)	0.03	0.002	0.0021176	0.16%	5242
0-17 years	1.11 (1.06, 1.17)	0.03	4.00E-05	0.00012	0.20%	5988

The table provides the percentage of variance explained (R^2 (%)) by PGS for retrospectively reported childhood maltreatment for prospectively reported childhood maltreatment in ALSPAC. Also provided are the odds ratio (log regression beta), and the associated 95% confidence interval, standard error, and p-value for childhood maltreatment reported at four different age ranges. Adjusted p-values are provided after correcting for multiple tests using Benjamini-Hochberg FDR correction. In additional models, we additionally accounted for prenatal (i.e. before the child was born) parental depression, childhood maltreatment, alcohol consumption, and smoking - familial factors associated with childhood maltreatment. All PGS were constructed at a p-value threshold = 1 (i.e. all SNPs after clumping for LD), as this explained the maximum variance in the hold-out sample in the UKBB (see Supplementary Table 7).

Supplementary Table 9: Lead SNPs from all independent loci with p-value < 5E-08 in the GWAS meta-analysis of childhood maltreatment

					Meta-analysis				Prospective childhood maltreatment				Retrospective childhood maltreatment			
SNP	Chr:Pos	EA	OA	EA freq	Beta	SE	p-value	N	Beta	SE	p-value	N	Beta	SE	p-value	N
rs12031035	1:76631858	a	t	0.20	0.024	0.004	2.28E-08	185414	0.018	0.014	0.205	15651	0.024	0.004	4.74E-08	169763
rs61818983	1:189921727	a	g	0.52	0.020	0.004	1.64E-08	171956	0.031	0.019	0.102	5400	0.020	0.004	5.38E-08	166556
rs3851357	3:155267440	a	g	0.18	-0.025	0.004	2.79E-08	180622	-0.043	0.015	0.004	15651	-0.023	0.005	9.69E-07	164971
rs611531	4:119187632	a	g	0.22	-0.024	0.004	1.02E-08	182382	-0.027	0.014	0.048	15651	-0.023	0.004	7.41E-08	166731
rs4895718	6:147973296	t	c	0.66	-0.020	0.004	4.59E-08	185414	-0.029	0.012	0.014	15651	-0.019	0.004	7.10E-07	169763
rs7763390	6:28714761	a	c	0.84	-0.027	0.005	7.45E-09	182382	-0.039	0.016	0.013	15651	-0.026	0.005	1.31E-07	166731
rs1859100	7:114194615	t	g	0.40	-0.021	0.003	8.70E-10	185414	-0.005	0.012	0.666	15651	-0.023	0.004	3.13E-10	169763
rs4305836	7:115008063	c	g	0.50	0.020	0.003	3.40E-09	185414	0.019	0.011	0.098	15651	0.020	0.004	1.37E-08	169763
rs3896224	10:106467853	a	g	0.58	0.020	0.003	7.10E-09	182382	0.012	0.011	0.288	15651	0.021	0.004	9.68E-09	166731
rs35560901	13:67054045	a	g	0.32	-0.024	0.004	4.84E-11	182382	-0.026	0.012	0.032	15651	-0.024	0.004	5.06E-10	166731
rs77987546	14:98582625	t	c	0.38	0.021	0.004	7.28E-09	172131	0.013	0.020	0.523	5400	0.021	0.004	8.16E-09	166731
rs4702	15:91426560	a	g	0.55	-0.026	0.003	1.01E-13	181033	-0.023	0.011	0.048	15651	-0.026	0.004	7.11E-13	165382
rs5928362	X:29802539	a	t	0.55	-0.021	0.004	4.30E-08	155268	NA	NA	NA	NA	-0.021	0.004	4.30E-08	155268
rs6633421	X:21569920	a	g	0.86	0.031	0.005	7.70E-09	155268	NA	NA	NA	NA	0.031	0.005	7.70E-09	155268

The table provides the lead SNPs identified in the 14 independent loci associated with childhood maltreatment (meta-analysis of prospectively and retrospectively reported childhood maltreatment). We provide the Chromosomal position (Chr: Pos), effect allele (EA), non-effect allele (OA), effect allele frequency (EA freq), regression beta (Beta) and associated standard error (SE) and p-value and sample size (N) for the meta-analysis, GWAS of prospectively and retrospectively reported childhood maltreatment respectively.

Supplementary Table 10: GWAS associations of SNPs in the independent loci with other phenotypes

IndSigSNP	Chr	BP	SNP	p-value	PMID	Trait
rs4895718	6	1.48E+08	rs725616	1.00E-09	30718901	Depression
rs4895718	6	1.48E+08	rs1147851	5.00E-09	29500382	Feeling tense
rs1859100	7	1.14E+08	rs10228494	1.00E-19	30643258	Adventurousness
rs1859101	7	1.14E+08	rs1015511	1.00E-13	30643258	Number of sexual partners
rs1859102	7	1.14E+08	rs727644	2.00E-12	30181555	Self-reported risk-taking behaviour
rs1859103	7	1.14E+08	rs727644	4.00E-14	30271922	Self-reported risk-taking behaviour
rs1859104	7	1.14E+08	rs1476535	6.00E-10	30610198	Attention deficit hyperactivity disorder or cannabis use
rs1859105	7	1.14E+08	rs2189012	1.00E-08	30610198	Attention deficit hyperactivity disorder or cannabis use
rs1859106	7	1.14E+08	rs727644	1.00E-34	30643258	General risk tolerance (MTAG)
rs1859108	7	1.14E+08	rs10280045	2.00E-11	30804566	Insomnia symptoms (never/rarely vs. usually)
rs1859109	7	1.14E+08	rs2189008	7.00E-09	30595370	Morning person
rs1859110	7	1.14E+08	rs1229762	1.00E-12	30846698	Sleep duration (short sleep)
rs4305836	7	1.15E+08	rs4377898	8.00E-31	30643258	General risk tolerance (MTAG)
rs4305836	7	1.15E+08	rs2106525	2.00E-10	30038396	Highest math class taken (MTAG)
rs4305836	7	1.15E+08	rs10251192	8.00E-15	30643258	Adventurousness
rs4305836	7	1.15E+08	rs4377898	2.00E-11	30643258	Number of sexual partners
rs4305836	7	1.15E+08	rs2401924	2.00E-08	30643258	Risk-taking tendency (4-domain principal component model)
rs4305836	7	1.15E+08	rs1358391	2.00E-08	30271922	Self-reported risk-taking behaviour
rs4305836	7	1.15E+08	rs2401924	1.00E-12	30595370	Smoking status
rs3896224	10	1.06E+08	rs3896224	5.00E-14	30038396	Cognitive performance (MTAG)
rs3896224	10	1.06E+08	rs11599236	3.00E-11	29942085	Depressed affect
rs3896224	10	1.06E+08	rs11599236	1.00E-13	30038396	Educational attainment (years of education)
rs3896224	10	1.06E+08	rs11599236	2.00E-12	29500382	Feeling miserable
rs3896224	10	1.06E+08	rs11596214	7.00E-09	30867560	General factor of neuroticism
rs3896224	10	1.06E+08	rs3896224	3.00E-08	29942086	Intelligence

rs3896224	10	1.06E+08	rs9787523	1.00E-09	30643251	Smoking initiation (ever regular vs never regular)
rs3896224	10	1.06E+08	rs9787523	6.00E-14	30643251	Smoking initiation (ever regular vs never regular) (MTAG)
rs3896224	10	1.06E+08	rs3896224	7.00E-10	30595370	Smoking status
rs3896224	10	1.06E+08	rs11599236	1.00E-15	30643256	Well-being spectrum (multivariate analysis)
rs35560901	13	67013049	rs9592470	3.00E-08	30643256	Neuroticism
rs77987546	14	98588321	rs7152623	3.00E-15	22068335	Aortic stiffness
rs77987546	14	98532540	rs710284	2.00E-08	30696823	Chronotype
rs77987546	14	98532540	rs710284	4.00E-11	30643251	Cigarettes smoked per day (MTAG)
rs77987546	14	98597552	rs1381287	1.00E-16	30643258	General risk tolerance (MTAG)
rs77987546	14	98587630	rs9323988	4.00E-11	28135244	Pulse pressure
rs77987546	14	98619968	rs17700977	6.00E-09	30643258	Adventurousness
rs77987546	14	98549383	rs10782490	5.00E-19	30643258	Number of sexual partners
rs77987546	14	98597552	rs1381287	1.00E-08	30271922	Self-reported risk-taking behaviour
rs77987546	14	98621512	rs10139768	3.00E-09	30643251	Smoking cessation (MTAG)
rs77987546	14	98597552	rs1381287	2.00E-12	30643251	Smoking initiation (ever regular vs never regular)
rs77987546	14	98597552	rs1381287	3.00E-17	30643251	Smoking initiation (ever regular vs never regular) (MTAG)
rs77987546	14	98588735	rs10147464	3.00E-14	30595370	Systolic blood pressure
rs77987546	14	98586162	rs7161578	4.00E-08	28928442	Yeast infection
rs4702	15	91426560	rs4702	1.00E-10	28540026	Autism spectrum disorder or schizophrenia
rs4702	15	91426560	rs4702	2.00E-09	29500382	Feeling hurt
rs4702	15	91426560	rs4702	3.00E-09	30643258	General risk tolerance (MTAG)
rs4702	15	91426560	rs4702	8.00E-14	25056061	Schizophrenia
rs4702	15	91426560	rs4702	3.00E-12	26198764	Schizophrenia
rs4702	15	91426560	rs4702	3.00E-10	27089180	Age at first sexual intercourse
rs4702	15	91426560	rs4702	6.00E-17	30643258	Number of sexual partners
rs4702	15	91423543	rs6224	5.00E-11	30595370	Balding type 1
rs4702	15	91416550	rs17514846	8.00E-27	29212778	Coronary artery disease
rs4702	15	91416550	rs17514846	1.00E-26	29212778	Coronary artery disease
rs4702	15	91428197	rs2071382	7.00E-13	28714975	Coronary artery disease

rs4702	15	91416550	rs17514846	3.00E-09	30487518	Hypertension
rs4702	15	91422543	rs8039305	7.00E-09	30487518	Hypertension
rs4702	15	91428197	rs2071382	1.00E-13	30573740	Male-pattern baldness
rs4702	15	91416550	rs17514846	2.00E-10	27618448	Mean arterial pressure
rs4702	15	91416550	rs17514846	7.00E-10	29227965	Parental longevity
rs4702	15	91428197	rs2071382	2.00E-19	30578418	Pulse pressure
rs4702	15	91416550	rs17514846	3.00E-12	29483656	Schizophrenia
rs4702	15	91416550	rs17514846	6.00E-11	30285260	Schizophrenia
rs4702	15	91428290	rs11539637	4.00E-09	30285260	Schizophrenia
rs4702	15	91416550	rs17514846	3.00E-08	29403010	Systolic blood pressure
rs4702	15	91416550	rs17514846	5.00E-09	30487518	Systolic blood pressure
rs4702	15	91428197	rs2071382	2.00E-32	30578418	Systolic blood pressure
rs4702	15	91418297	rs8032315	2.00E-08	28540026	Autism spectrum disorder or schizophrenia
rs4702	15	91427612	rs12906125	1.00E-08	27680694	Birth weight
rs4702	15	91437388	rs2521501	3.00E-08	21909110	Blood pressure
rs4702	15	91428955	rs1894400	1.00E-30	30595370	Cardiovascular disease
rs4702	15	91429287	rs4932373	2.00E-25	29212778	Coronary artery disease
rs4702	15	91404788	rs4932371	1.00E-17	27841878	Diastolic blood pressure
rs4702	15	91437388	rs2521501	2.00E-15	21909115	Diastolic blood pressure
rs4702	15	91437388	rs2521501	1.00E-13	28739976	Diastolic blood pressure
rs4702	15	91437388	rs2521501	2.00E-17	27618452	Diastolic blood pressure
rs4702	15	91437388	rs2521501	2.00E-22	27841878	Diastolic blood pressure
rs4702	15	91437388	rs2521501	3.00E-40	29455858	Diastolic blood pressure (cigarette smoking interaction)
rs4702	15	91409514	rs8029440	1.00E-17	29912962	Diastolic blood pressure x alcohol consumption
rs4702	15	91428955	rs1894400	2.00E-19	29912962	Diastolic blood pressure x alcohol consumption
rs4702	15	91420973	rs1573643	6.00E-37	29912962	Diastolic blood pressure x alcohol consumption
rs4702	15	91437388	rs2521501	3.00E-31	29912962	Diastolic blood pressure x alcohol consumption
rs4702	15	91429287	rs4932373	9.00E-10	30595370	Hair colour
rs4702	15	91404705	rs4932370	3.00E-08	29531354	Ischemic stroke

rs4702	15	91428955	rs1894400	6.00E-23	29912962	Mean arterial pressure x alcohol consumption
rs4702	15	91420973	rs1573643	1.00E-24	29912962	Mean arterial pressure x alcohol consumption
rs4702	15	91427872	rs35346340	5.00E-19	29912962	Mean arterial pressure x alcohol consumption interaction
rs4702	15	91437388	rs2521501	7.00E-19	29912962	Mean arterial pressure x alcohol consumption interaction
rs4702	15	91404788	rs4932371	2.00E-13	27841878	Pulse pressure
rs4702	15	91437388	rs2521501	7.00E-14	27841878	Pulse pressure
rs4702	15	91428955	rs1894400	4.00E-19	29912962	Pulse pressure x alcohol consumption interaction (2df test)
rs4702	15	91418297	rs8032315	6.00E-09	30643258	Risk-taking tendency (4-domain principal component model)
rs4702	15	91404788	rs4932371	1.00E-23	27841878	Systolic blood pressure
rs4702	15	91404788	rs4932371	1.00E-08	27841878	Systolic blood pressure
rs4702	15	91437388	rs2521501	5.00E-19	21909115	Systolic blood pressure
rs4702	15	91437388	rs2521501	1.00E-12	28739976	Systolic blood pressure
rs4702	15	91437388	rs2521501	3.00E-20	27618452	Systolic blood pressure
rs4702	15	91437388	rs2521501	1.00E-59	30595370	Systolic blood pressure
rs4702	15	91437388	rs2521501	2.00E-26	27841878	Systolic blood pressure
rs4702	15	91437388	rs2521501	6.00E-45	29455858	Systolic blood pressure (cigarette smoking interaction)
rs4702	15	91420973	rs1573643	5.00E-22	29912962	Systolic blood pressure x alcohol consumption
rs4702	15	91420940	rs2071410	1.00E-40	29912962	Systolic blood pressure x alcohol consumption interaction
rs4702	15	91429176	rs7497304	8.00E-41	29912962	Systolic blood pressure x alcohol consumption interaction
rs6633421	23	21380266	rs1378559	2.00E-12	25056061	Schizophrenia

The table provides the significant associations of SNPs in the top loci that are significantly genetically associated with other phenotypes (GWAS, $p < 5E-08$).

Supplementary Table 11: GWAS associations of SNPs in the independent loci with other phenotypes

Gene Symbol	Chr	Start	End	Pos Map SNPs	Eqtl map min q-value	eqtlMapts	ciMapts	Min GwasP	IndSigSNPs
ACADM	1	76190036	76253260	0	0.040	PsychENCODE_eQTLs	NA	1.49E-07	rs12031035
ST6GALNAC3	1	76540404	77100286	75	0.029	GTEx_v8 Brain_Cerebellar_Hemisphere	NA	2.28E-08	rs12031035
ST6GALNAC5	1	77333126	77531396	0	NA	NA	Adult Cortex, Fetal Cortex, Neural Progenitor Cell	2.28E-08	rs12031035
PLCH1	3	1.55E+08	1.55E+08	7	NA	NA	NA	2.79E-08	rs3851357
TRAM1L1	4	1.18E+08	1.18E+08	0	NA	NA	Fetal Cortex	1.02E-08	rs611531
NDST3	4	1.19E+08	1.19E+08	37	NA	NA	Adult Cortex, Fetal Cortex	1.02E-08	rs611531
PRSS12	4	1.19E+08	1.19E+08	12	8.9E-11	CMC_SVA_cis, GTEx_v8Brain_Nucleus_accumbens_basal_ganglia	NA	1.02E-08	rs611531
ZSCAN9	6	28192664	28201260	0	0.009	CMC_SVA_cis, CMC_NoSVA_cis	NA	7.45E-09	rs7763390
ZSCAN31	6	28292470	28324048	0	4.3E-20	CMC_SVA_cis, CMC_NoSVA_cis, BRAINEAC/CRBL, BRAINEAC/FCTX, BRAINEAC/aveALL,	NA	7.45E-09	rs7763390

						GTEx/v8/Brain_Anterior_cingulate_cortex_BA24, GTEx/v8/Brain_Caudate_basal_ganglia, GTEx/v8/Brain_Cerebellar_Hemisphere:GTEx/v8/Brain_Cerebellum, GTEx/v8/Brain_Cortex:GTEx/v8/Brain_Frontal_Cortex_BA9, GTEx/v8/Brain_Hippocampus, GTEx/v8/Brain_Putamen_basal_ganglia, GTEx/v7/Brain_Anterior_cingulate_cortex_BA24, GTEx/v7/Brain_Cortex			
ZKSCAN3	6	28317691	28336947	0	0.049	CMC_SVA_cis	NA	7.45E-09	rs7763390
ZSCAN23	6	28399707	28411279	0	1.5E-18	CMC_SVA_cis, BRAINEAC/CRBL, GTEx/v8/Brain_Cerebellum, GTEx/v7/Brain_Caudate_basal_ganglia, GTEx/v7/Brain_Cerebellar_Hemisphere, GTEx/v6/Brain_Cerebellar_Hemisphere	NA	7.45E-09	rs7763390

C6orf100	6	28911654	28912314	0	9.2E-09	GTEx/v8/Brain_Nucleus_accumbens_basal_ganglia	NA	1.95E-06	rs7763390
ZNF311	6	28962562	28973093	0	NA	NA	Neural Progenitor Cell	1.47E-06	rs7763390
SAMD5	6	1.48E+08	1.48E+08	63	2.8E-10	PsychENCODE_eQTLs, GTEx/v8/Brain_Cerebellar_Hemisphere, GTEx/v8/Brain_Cerebellum, GTEx/v8/Brain_Hypothalamus, GTEx/v7/Brain_Cerebellar_Hemisphere, GTEx/v7/Brain_Cerebellum, GTEx/v6/Brain_Cerebellum	Fetal Cortex	4.59E-08	rs4895718
FOXP2	7	1.14E+08	1.14E+08	49	0.049	CMC_SVA_cis	Adult Cortex, Fetal Cortex, Neural Progenitor Cell	6.32E-10	rs34948690; rs34948690: rs4305836
MDFIC	7	1.15E+08	1.15E+08	0	NA	NA	Fetal_Cortex, Neural Progenitor Cell	6.50E-09	rs34948690: rs4305836
SORCS3	10	1.06E+08	1.07E+08	25	0.007	PsychENCODE_eQTLs	Promoter anchored loops, Adult Cortex	7.10E-09	rs3896224; rs17185757; rs17185757: rs3896224

PCDH9	13	66876967	67804468	54	NA	NA	NA	4.84E-11	rs9529067; rs35560901
VRK1	14	97263641	97398059	0	NA	NA	Fetal Cortex	1.45E-08	rs79019742
GABARAPL3	15	90890819	90892669	0	NA	NA	Fetal Cortex	3.80E-10	rs772992986
ZNF774	15	90895477	90909324	0	NA	NA	Fetal Cortex	3.80E-10	rs772992986
BLM	15	91260558	91358859	0	NA	NA	Fetal Cortex	3.80E-10	rs772992986
FURIN	15	91411822	91426688	32	3.3E-05	PsychENCODE_eQTLs, CMC_SVA_cis, CMC_NoSVA_cis, GTEx/v8/Brain_Frontal_Cortex_BA9	NA	1.01E-13	rs772992986; rs6145676; rs4702
FES	15	91426925	91439006	23	1.9E-05	PsychENCODE_eQTLs, CMC_SVA_cis	Promoter anchored loops	1.01E-13	rs772992986; rs4702; rs6145676
MAN2A2	15	91445448	91465814	1	NA	NA	NA	3.66E-10	rs772992986
UNC45A	15	91473410	91497323	0	NA	NA	Adult Cortex, Fetal Cortex	3.80E-10	rs772992986
HDDC3	15	91474148	91475799	0	NA	NA	Adult Cortex, Fetal Cortex	3.80E-10	rs772992986
RCCD1	15	91498100	91506349	0	4.3E-22	GTEx/Brain_Nucleus_accumbens_basal_ganglia, GTEx/v7/Brain_Cerebellar_Hemisphere	NA	1.01E-13	rs772992986; rs4702
CNKSR2	23	21392536	21672813	3	NA	NA	NA	7.70E-09	rs6633421

The table provides the significant genes identified from for the top loci (p < 5E-08) from the GWAS meta-analysis of childhood maltreatment. Genes were identified using positional mapping, neuronal eQTLs, chromatin conformation data from neuronal tissues.

Supplementary Table 12: MAGMA – gene-based association statistics

GENE	CHR	START	STOP	NSNPS	NPARAM	N	ZSTAT	p-value	SYMBOL
ENSG00000182511	15	91426925	91439006	37	7	185000	6.7895	5.63E-12	FES
ENSG00000140564	15	91411822	91426688	35	8	185000	6.3869	8.47E-11	FURIN
ENSG00000184226	13	66876967	67804468	2694	196	185000	5.8898	1.93E-09	PCDH9
ENSG00000156395	10	106400859	107024993	2902	105	185000	5.8742	2.12E-09	SORCS3
ENSG00000128573	7	113726382	114333827	1474	105	185000	5.6294	9.04E-09	FOXP2
ENSG00000114861	3	71003844	71633140	1856	205	185000	5.3062	5.60E-08	FOXP1
ENSG00000265303	17	57274927	57292587	53	12	185000	5.2528	7.49E-08	CTD-2510F5.6
ENSG00000068489	17	57232860	57282066	113	18	185000	5.1412	1.36E-07	PRR11
ENSG00000153982	17	57297828	57353328	141	17	185000	5.1196	1.53E-07	GDPD1
ENSG00000100242	22	39130730	39190148	150	31	185000	5.0838	1.85E-07	SUN2
ENSG00000203727	6	147830063	148058683	1297	103	185000	5.0729	1.96E-07	SAMD5
ENSG00000184005	1	76540404	77100286	2071	180	185000	4.9536	3.64E-07	ST6GALNAC3
ENSG00000145934	5	166711804	167691162	2884	316	185000	4.8561	5.98E-07	TENM2
ENSG00000106536	7	39017598	39532694	2030	104	185000	4.8545	6.03E-07	POU6F2
ENSG00000111725	12	120105558	120119435	31	7	185000	4.8366	6.60E-07	PRKAB1
ENSG00000138738	4	121606074	121844025	1167	57	185000	4.8357	6.63E-07	PRDM5
ENSG00000166947	15	43398423	43513481	220	57	185000	4.6909	1.36E-06	EPB42
ENSG00000164483	6	130465460	130686570	1278	54	185000	4.6692	1.51E-06	SAMD3
ENSG00000137872	15	47476298	48066420	2241	100	185000	4.6092	2.02E-06	SEMA6D

The table provides the significant genes identified from MAGMA after Bonferroni correction. For each gene, we provide the Z statistics, and the association p-value, alongside the chromosomal location and number of SNPs in the gene.

Supplementary Table 13: LDSC-SEG based enrichment analyses for chromatin marks

Name	Coefficient	SE	p-value
Fetal_Brain_Male__DNase	1.30E-07	2.47E-08	7.03E-08
Fetal_Brain_Female__DNase	1.45E-07	2.92E-08	3.76E-07
Brain_Germinal_Matrix__H3K4me3	1.78E-07	4.64E-08	6.30E-05
Fetal_Brain_Female__H3K4me3	1.51E-07	3.98E-08	7.47E-05
Fetal_Brain_Male__H3K4me1	4.05E-08	1.10E-08	1.13E-04
Brain_Angular_Gyrus__H3K27ac	4.88E-08	1.33E-08	1.28E-04
Fetal_Brain_Female__H3K4me1	6.79E-08	1.88E-08	1.48E-04
Brain_Dorsolateral_Prefrontal_Cortex__H3K27ac	5.14E-08	1.49E-08	2.75E-04
Brain_Angular_Gyrus__H3K9ac	9.42E-08	2.83E-08	4.41E-04
Brain_Inferior_Temporal_Lobe__H3K9ac	7.64E-08	2.37E-08	6.43E-04

The table provides the results of the enrichment analyses for tissue specific chromatin marks in multiple. We display only the significant results (after Benjamini-Hochberg FDR correction). We provide the coefficients, the associated standard errors, and the p-values. No

Supplementary Table 14: MAGMA based enrichment analyses for genes with cell-specific expression

Cell type	N genes	Beta	SE	P
Ex4	14339	0.35564	0.1224	0.001837
In4a	14339	0.19312	0.11921	0.052627
In1a	14339	0.14691	0.11561	0.10193
Ex6a	14339	0.093696	0.082975	0.12942
OPC	14339	0.11222	0.11824	0.1713
Oligo	14339	0.09731	0.12336	0.21512
Per	14339	0.13714	0.18155	0.22501
In8	14339	0.085933	0.14123	0.27145
Microglia	14339	0.078812	0.16271	0.31407
In1b	14339	0.059341	0.13638	0.33175
Astro	14339	0.015626	0.08832	0.42979
Ex5b	14339	0.010896	0.093989	0.45386
Ex2	14339	0.009459	0.081893	0.45402
Ex8	14339	0.003891	0.076769	0.47979
In4b	14339	-0.00818	0.098407	0.53312
In3	14339	-0.01481	0.14025	0.54204
Ex3e	14339	-0.03333	0.10532	0.62418
Ex9	14339	-0.04053	0.06	0.75034
In6b	14339	-0.06927	0.10131	0.75293
Ex6b	14339	-0.0807	0.098887	0.79277
In6a	14339	-0.10326	0.10359	0.84055
In1c	14339	-0.14365	0.13611	0.85438
In7	14339	-0.14821	0.11904	0.89343
Ex1	14339	-0.123	0.096957	0.89769
Endo	14339	-0.08953	0.054654	0.94929

The table provides the results of the enrichment analyses for multiple neuronal cell types (top 10%). We provide the coefficients, the associated standard errors, and the p-values. Significant enrichments after Bonferroni correction are highlighted in red.

Supplementary Table 15: MAGMA based enrichment analyses for genes with tissue-specific expression

Tissue type	N genes	Beta	SE	p-value
Cells_EBV-transformed_lympho...	18236	0.011132	0.004811	0.010341
Spleen	18236	0.014636	0.007029	0.018671
Brain_Caudate_basal_ganglia	18236	0.013864	0.007742	0.036666
Brain_Nucleus_accumbens_basa...	18236	0.011527	0.007347	0.058334
Brain_Putamen_basal_ganglia	18236	0.011609	0.007716	0.066221
Whole_Blood	18236	0.007879	0.005468	0.074818
Brain_Cerebellum	18236	0.008784	0.006109	0.075231
Brain_Cerebellar_Hemisphere	18236	0.008384	0.005904	0.077796
Pituitary	18236	0.0094	0.00864	0.1383
Brain_Spinal_cord_cervical_c...	18236	0.008824	0.008487	0.14924
Small_Intestine_Terminal_Ile...	18236	0.008181	0.008914	0.17939
Cells_Transformed_fibroblasts	18236	0.006023	0.006573	0.17975
Colon_Transverse	18236	0.009371	0.010819	0.1932
Brain_Substantia_nigra	18236	0.006998	0.008357	0.20119
Brain_Hippocampus	18236	0.006499	0.007787	0.202
Brain_Hypothalamus	18236	0.005959	0.007788	0.22207
Brain_Amygdala	18236	0.005645	0.007613	0.22919
Brain_Anterior_cingulate_cor...	18236	0.005002	0.007158	0.24235
Brain_Cortex	18236	0.00447	0.006914	0.25899
Brain_Frontal_Cortex_BA9	18236	0.00399	0.006633	0.27377
Testis	18236	0.00321	0.005449	0.27793
Adrenal_Gland	18236	0.003621	0.009482	0.35127
Pancreas	18236	0.001449	0.008144	0.42941
Heart_Atrial_Appendage	18236	0.001056	0.009267	0.45463
Liver	18236	0.000593	0.006203	0.46191
Colon_Sigmoid	18236	0.000644	0.011927	0.47847
Kidney_Cortex	18236	-0.00068	0.008875	0.53065
Lung	18236	-0.00237	0.00887	0.60527
Stomach	18236	-0.00429	0.011346	0.64719
Artery_Aorta	18236	-0.00565	0.010016	0.71378
Esophagus_Muscularis	18236	-0.00781	0.012001	0.74241
Artery_Tibial	18236	-0.00673	0.009875	0.75226
Nerve_Tibial	18236	-0.00735	0.009992	0.76901
Bladder	18236	-0.00901	0.011218	0.78901
Esophagus_Mucosa	18236	-0.00653	0.007383	0.81175
Ovary	18236	-0.00845	0.009248	0.81944
Esophagus_Gastroesophageal_J...	18236	-0.01171	0.01238	0.82785
Thyroid	18236	-0.00912	0.00937	0.83477
Heart_Left_Ventricle	18236	-0.00859	0.008712	0.83791
Artery_Coronary	18236	-0.01181	0.01156	0.84652
Uterus	18236	-0.01194	0.010574	0.8705

Fallopian_Tube	18236	-0.01559	0.011295	0.91617
Prostate	18236	-0.02006	0.011681	0.957
Adipose_Subcutaneous	18236	-0.01833	0.010366	0.9615
Vagina	18236	-0.01916	0.010651	0.96396
Cervix_Ectocervix	18236	-0.0226	0.012261	0.96735
Muscle_Skeletal	18236	-0.01248	0.00671	0.96852
Cervix_Endocervix	18236	-0.02185	0.010944	0.97707
Minor_Salivary_Gland	18236	-0.01943	0.009407	0.98057
Skin_Sun_Exposed_Lower_leg	18236	-0.01664	0.007518	0.98656
Skin_Not_Sun_Exposed_Suprapu...	18236	-0.01751	0.007567	0.98966
Breast_Mammary_Tissue	18236	-0.03048	0.012174	0.99385
Adipose_Visceral_Omentum	18236	-0.03111	0.010795	0.99802

The table provides the results of the enrichment analyses for genes with tissue specific expression across multiple tissues (top 10%). We provide the coefficients, the associated standard errors, and the p-values. No results were statistically significant after Bonferroni correction.

Supplementary Table 16: LDSC based enrichment analyses for genes with tissue-specific expression

Name	Coefficient	SE	p-value
Brain_Anterior_cingulate_cortex_(BA24)	2.84E-09	1.44E-09	0.02
Brain_Cortex	2.45E-09	1.60E-09	0.06
Brain_Frontal_Cortex_(BA9)	1.28E-09	1.37E-09	0.17
Brain_Cerebellum	1.31E-09	1.51E-09	0.19
Brain_Caudate_(basal_ganglia)	6.28E-10	1.38E-09	0.32
Brain_Cerebellar_Hemisphere	3.78E-10	1.52E-09	0.40
Brain_Hippocampus	1.15E-10	1.35E-09	0.46
Brain_Amygdala	9.03E-11	1.57E-09	0.47
Brain_Hypothalamus	-1.15E-10	1.42E-09	0.53
Brain_Nucleus_accumbens_(basal_ganglia)	-7.86E-10	1.26E-09	0.73
Brain_Spinal_cord_(cervical_c-1)	-8.41E-10	1.32E-09	0.73
Brain_Substantia_nigra	-9.91E-10	1.24E-09	0.78
Brain_Putamen_(basal_ganglia)	-1.37E-09	1.43E-09	0.83

The table provides the results of the enrichment analyses for genes with tissue specific expression in brain regions (top 10%). We provide the coefficients, the associated standard errors, and the p-values. No results were statistically significant after Bonferroni correction.

Supplementary Table 17: Genetic correlation analyses

Phenotype	PMID	r_g	SE	p-value
Major depressive disorder	29700475	0.633	0.034	4.27E-78
Neuroticism	29942085	0.392	0.023	1.70E-66
Depressive symptoms	27089181	0.649	0.043	1.03E-50
Attention deficit hyperactivity disorder	30478444	0.557	0.039	6.63E-46
Schizophrenia	29483656	0.365	0.028	8.30E-40
Age of first birth	27798627	-0.408	0.036	1.14E-29
Subjective well being	27089181	-0.519	0.050	9.59E-26
Age of smoking initiation	20418890	-0.315	0.034	1.22E-20
PGC cross-disorder analysis	23453885	0.420	0.046	3.77E-20
Autism spectrum disorder	30804558	0.412	0.047	8.82E-19
Intelligence	29942086	-0.202	0.026	1.20E-15
Years of schooling	30038396	-0.190	0.024	1.30E-15
Ever vs never smoked	20418890	0.332	0.048	3.78E-12
Insomnia	28604731	0.325	0.049	2.99E-11
Obesity class 1	23563607	0.172	0.029	2.60E-09
Fathers age at death	27015805	-0.349	0.063	2.46E-08
College completion	23722424	-0.237	0.043	5.04E-08
Number of children ever born	27798627	0.249	0.046	6.23E-08
Body mass index	20935630	0.149	0.029	2.16E-07
Obesity class 2	23563607	0.193	0.038	2.84E-07
Body fat	26833246	0.195	0.038	3.42E-07
Overweight	23563607	0.151	0.032	2.38E-06
Former vs Current smoker	20418890	-0.330	0.072	4.48E-06
Waist-to-hip ratio	25673412	0.143	0.034	2.20E-05
Waist circumference	25673412	0.130	0.031	2.88E-05
Age at Menarche	25231870	-0.111	0.028	5.30E-05
Extreme bmi	23563607	0.172	0.043	6.14E-05
Mothers age at death	27015805	-0.267	0.069	9.46E-05
Parents age at death	27015805	-0.266	0.070	2.00E-04
Coronary artery disease	26343387	0.132	0.035	2.00E-04
Bipolar disorder	21926972	0.176	0.047	2.00E-04
Smoking Initiation	30617275	0.243	0.068	3.00E-04
Childhood IQ	23358156	-0.263	0.074	4.00E-04
Obesity class 3	23563607	0.178	0.051	5.00E-04
Lung cancer	27488534	0.224	0.064	5.00E-04
Lung cancer (all)	24880342	0.218	0.065	8.00E-04
Squamous cell lung cancer	27488534	0.312	0.099	1.70E-03
HDL cholesterol	20686565	-0.113	0.037	2.20E-03
Lung cancer (squamous cell)	24880342	0.326	0.110	3.00E-03
Hip circumference	25673412	0.090	0.031	3.60E-03
Attention deficit hyperactivity disorder	20732625	0.286	0.102	4.90E-03
Childhood obesity	22484627	0.132	0.048	5.70E-03

Cigarettes smoked per day	20418890	0.223	0.081	6.00E-03
Excessive daytime sleepiness	27992416	0.116	0.043	6.70E-03
Alzheimer's disease	30617256	0.230	0.087	7.90E-03
Lung adenocarcinoma	27488534	0.253	0.099	1.03E-02
Serum cystatin c	26831199	-0.113	0.047	1.69E-02
Fasting insulin main effect	22581228	0.142	0.063	2.33E-02
Chronotype	27494321	-0.088	0.040	2.55E-02
Triglycerides	20686565	0.078	0.036	3.10E-02
Urinary albumin-to-creatinine ratio	26631737	0.148	0.070	3.47E-02
HOMA-IR	20081858	0.142	0.068	3.60E-02
Pack Years	30617275	0.322	0.156	3.92E-02
Neo-conscientiousness	21173776	-0.202	0.100	4.41E-02
Fasting proinsulin	20081858	0.179	0.095	5.98E-02
Fasting glucose main effect	22581228	0.092	0.051	7.11E-02
Urinary albumin-to-creatinine ratio (non-diabetes)	26631737	0.125	0.074	8.90E-02
Smoking Cessation	30617275	-0.231	0.143	1.06E-01
Type 2 Diabetes	22885922	0.077	0.049	1.19E-01
Child birth length	25281659	-0.093	0.060	1.21E-01
Mean Hippocampus	25607358	-0.123	0.080	1.28E-01
Age at Menopause	26414677	-0.063	0.041	1.29E-01
Sitting height ratio	25865494	-0.091	0.060	1.31E-01
Leptin not adjBMI	26833098	0.089	0.059	1.34E-01
Amyotrophic lateral sclerosis	27455348	0.125	0.084	1.34E-01
HbA1C	20858683	0.087	0.067	1.91E-01
Sleep duration	27494321	-0.065	0.051	1.98E-01
Neo-openness to experience	21173776	0.109	0.089	2.21E-01
Chronic Kidney Disease	26831199	-0.088	0.072	2.21E-01
Infant head circumference	22504419	-0.082	0.075	2.74E-01
2hr glucose adjusted for BMI	20081857	-0.089	0.086	3.01E-01
Anorexia Nervosa	31308545	-0.065	0.066	3.21E-01
Mean Caudate	25607358	0.063	0.071	3.77E-01
HOMA-B	20081858	0.049	0.057	3.88E-01
Birth weight	27680694	-0.027	0.033	4.04E-01
Serum creatinine	26831199	-0.028	0.034	4.08E-01
Mean Thalamus	25607358	-0.057	0.074	4.38E-01
Mean Accumbens	25607358	-0.078	0.112	4.86E-01
Parkinson's disease	19915575	0.037	0.053	4.86E-01
Serum creatinine (non-diabetes)	26831199	-0.023	0.035	5.10E-01
Height; Females at age 10 and males at age 12	23449627	-0.030	0.048	5.27E-01
Leptin_adjBMI	26833098	-0.038	0.063	5.46E-01
Mean Putamen	25607358	0.026	0.062	6.73E-01
Mean Pallidum	25607358	-0.027	0.073	7.10E-01
Own birth weight (foetal effect)	31043758	0.015	0.042	7.30E-01
Height_2010	20881960	-0.009	0.028	7.40E-01
Total Cholesterol	20686565	-0.013	0.039	7.42E-01

Cigarettes Per Day	30617275	0.019	0.058	7.43E-01
Extreme waist-to-hip ratio	23563607	0.023	0.072	7.54E-01
Own birth weight	31043758	0.007	0.030	8.26E-01
Offspring birth weight	31043758	0.007	0.032	8.30E-01
ICV	25607358	-0.016	0.077	8.39E-01
Adiponectin	22479202	0.010	0.065	8.79E-01
Own birth weight	31043758	-0.003	0.031	9.15E-01
Offspring birth weight (maternal effect)	31043758	0.003	0.033	9.27E-01
Extreme height	23563607	-0.002	0.035	9.50E-01
LDL cholesterol	20686565	0.002	0.043	9.62E-01

The table provides genetic correlations (r_g) between childhood maltreatment and a number of other phenotypes, associated standard errors (SE), and p-value. Significant genetic correlations after Bonferroni correction are highlighted in red.

Supplementary Table 18: MR analyses

				MR			Reverse MR				
S No.	Phenotype 1	Phenotype 2	Nsnps	Beta	SE	p-value	Nsnps	Beta	SE	p-value	Methods
1	Childhood maltreatment	Depression (without UKB and 23andMe, P < 1E-7)	10	0.598	0.145	3.63E-05	6	0.061	0.058	2.92E-01	IVW
			10	0.577	0.189	2.23E-03	6	0.004	0.043	9.34E-01	Weighted Median
			10	-0.984	1.51	5.33E-01	6	0.282	0.565	6.45E-01	Egger Regression
			10	0.598	0.145	3.63E-05	5	0.025	0.052	6.61E-01	MR PRESSO
2	Childhood maltreatment	Schizophrenia	14	1.167	0.268	1.35E-05	192	0.048	0.006	4.83E-15	IVW
			14	0.886	0.213	3.45E-05	192	0.042	0.007	7.27E-10	Weighted Median
			14	5.693*	1.59	3.78E-03	192	0.055	0.025	2.83E-02	Egger Regression
			11	0.887	0.164	2.98E-04	188	0.044	0.005	9.77E-16	MR PRESSO
3	Childhood maltreatment	ADHD	12	1.04	0.362	4.02E-03	11	0.083	0.022	1.22E-04	IVW
			12	1.097	0.291	1.63E-04	11	0.06	0.022	5.83E-03	Weighted Median
			12	-2.798	3.327	4.20E-01	11	-0.011	0.108	9.21E-01	Egger Regression
			10	1.054	0.277	4.20E-03	10	0.071	0.02	5.42E-03	MR PRESSO
4	Childhood maltreatment	Autism	12	0.359	0.29	2.16E-01	4	0.003	0.037	9.41E-01	IVW
			12	0.461	0.299	1.23E-01	4	-0.001	0.03	9.73E-01	Weighted Median
			12	-0.773	2.808	7.89E-01	4	0.008	0.148	9.63E-01	Egger Regression
			11	0.538	0.233	4.34E-02	4	0.003	0.037	9.41E-01	MR PRESSO
5	Childhood maltreatment	Bipolar Disorder	10	0.563	0.298	5.90E-02	21	0.027	0.01	8.60E-03	IVW
			10	0.638	0.327	5.10E-02	21	0.029	0.014	3.55E-02	Weighted Median
			10	2.085	3.271	5.24E-01	21	0.058	0.052	2.74E-01	Egger Regression
			10	0.563	0.298	5.90E-02	21	0.027	0.01	8.60E-03	MR PRESSO
6		Coronary Artery Disease	10	-0.024	0.254	9.24E-01	57	0.003	0.008	6.82E-01	IVW
			10	0.027	0.219	9.01E-01	57	0.022	0.01	2.92E-02	Weighted Median

	Childhood maltreatment		10	-6.283*	1.766	7.42E-03	57	-0.004	0.018	8.16E-01	Egger Regression
			9	0.193	0.196	3.55E-01	55	0.01	0.007	1.59E-01	MR PRESSO
7	Childhood maltreatment	Type 2 diabetes	13	0.21	0.231	3.65E-01	60	0.011	0.006	7.02E-02	IVW
			13	0.121	0.252	6.30E-01	60	0.016	0.008	4.32E-02	Weighted Median
			13	-0.205	2.285	9.30E-01	60	0.023	0.016	1.49E-01	Egger Regression
			13	0.21	0.231	3.65E-01	60	0.011	0.006	7.02E-02	MR PRESSO
8	Childhood maltreatment	C- Reactive Protein	14	-0.047	0.113	6.80E-01	58	0.007	0.007	3.63E-01	IVW
			14	0	0.15	9.98E-01	58	0.003	0.01	7.43E-01	Weighted Median
			14	0.308	0.927	9.98E-01	58	-0.002	0.015	8.77E-01	Egger Regression
			14	-0.047	0.113	6.80E-01	58	0.007	0.007	3.63E-01	MR PRESSO

The table provides the results of the Mendelian Randomization analyses. For each pair of phenotypes, we conduct MR (Childhood maltreatment causal for the secondary phenotype) and reverse MR (secondary phenotype causal for childhood maltreatment) using four different MR analyses: IVW, Weighted Median, MR Egger Regression, and MR PRESSO. For each of these MR analyses, we provide the number of SNPs included in the analyses, the regression beta, and the associated standard error (SE) and p-value. *Beta values with an asterisk indicates that the regression intercept in the MR Egger analysis was statistically significant. Please refer to Supplementary Figures 19 – 34 for plots (scatterplots, forest plots, and leave-one-out plots), and MR Egger intercept values and results of the Steiger analyses to test directionality of the instrument.

FAQs

1. What is this study about?

Childhood maltreatment is complex and is associated with several mental and physical health conditions. However, most of the current evidence for this is from observational studies. These studies do not provide sufficient evidence to say that childhood maltreatment *causes* adverse mental and physical health conditions.

Twin and familial studies suggest that childhood maltreatment is *partly* genetic. In other words, differences in people's experiences of childhood maltreatment can be attributed partly to the differences in their genetics. This genetic signal is thought to arise primarily through gene-environment correlations, where a person's genetic propensities correlate with their environment. This can happen passively as an individual inherits approximately half of their parents' DNA who in turn create the familial environment. This can also happen actively if an individual seeks out or creates certain environments partly as a result of genetic propensities. Finally, this can happen reactively, where an individual's behaviour or physical features (which is also partly genetic) causes other people to react to them in a certain manner.

By carefully studying the genetics signal associated with childhood maltreatment, we can use a recently developed method called Mendelian Randomization to identify potential causal effects of childhood maltreatment. Furthermore, we can use genetically informed methods to understand what the genetic component of childhood maltreatment means and how it differs between different subtypes and operationalizations of childhood maltreatment. This study was conducted to address these questions.

2. What did the study find?

We conducted the largest genetic association study of childhood maltreatment to date in over 140,000 individuals who provided reports on childhood maltreatment either measured during adulthood (called retrospectively measured childhood maltreatment) or measured during childhood (called prospectively measured childhood maltreatment). We identified 14 independent genetic regions (called loci) that are associated with childhood maltreatment.

We show that there is relatively high shared genetics between different subtypes, operationalizations and reports of childhood maltreatment.

We find that approximately 8% of the differences between people's experience of childhood maltreatment is due to the genetic variants that we studied. Further, using different within-family designs, we find that approximately 60% of the genetic effect (i.e. 60% of the 8%) is due to active and reactive gene-environment correlation. However, this does not indicate that there is no role for passive gene-environment correlation, and better methods in larger samples are needed to estimate the contributions of the various types of gene-environment correlations and interactions.

Finally, using Mendelian Randomization, we find evidence to suggest that childhood maltreatment is causal for depression, ADHD and schizophrenia. We do not, in this study, find

evidence for a causal effect of childhood maltreatment on physical health conditions like coronary artery disease, type 2 diabetes, or inflammation. Furthermore, we find some evidence to suggest that schizophrenia and ADHD is in a bidirectional way causal for childhood maltreatment.

3. Does the study find a gene for childhood maltreatment?
No, the study does not find a gene for childhood maltreatment. Genes do not directly shape childhood traumatic experiences. Instead, they contribute to how our brains develop, and how brains process information from the world. As a result, they partly shape our personalities, our behaviour and can influence how others interact with us. Thus, the genetic factors we have identified represent several biological processes which heavily interact with the environment to drive the association with childhood maltreatment.

4. Is the environment unimportant?
First, it is important to emphasize that the majority of the differences in reporting childhood maltreatment is still not captured by the genetic variants in this study. A substantial proportion may be environmental. So, it is crucial not to underestimate the contribution of environmental or social factors.

Second, these findings reflect gene-environment correlations. These associations, as mentioned above, represent a series of biological processes which interact with the environment.

Finally, it is plausible that modifying the environment can decrease or even mitigate the risk for childhood maltreatment as is shown in experimental intervention studies.

5. Can you use these findings to predict childhood maltreatment?
No, these findings cannot used for genetic testing. Less than 1% of the total variance in the population can be predicted using genetic scores. Furthermore, even with the same genetic predisposition, the risk of experiencing childhood maltreatment will differ across individuals depending on the environment. In that sense, genetics alone is not predictive and would lead to many false positives (and false negatives) in predictive testing.

6. Does evidence for active and reactive rGE indicate that a person is blame for their childhood maltreatment?
This is incorrect and the results absolutely and unequivocally do not suggest this. Victim blaming or discrimination of people who have experienced childhood maltreatment is not implied by our findings. It is important to remember that people experience childhood maltreatment when they are children, and the responsibility of protecting a child lies primarily with their parents and additionally with the wider social environment and society at large. As mentioned earlier, the genetic signal represents gene-environment correlation, and a supportive environment may decrease or even minimize the risk for childhood maltreatment. The aim of this research is to understand the causes and consequences of childhood maltreatment, knowledge needed to mitigate them. It is important to provide support to individuals who have experienced childhood maltreatment to counteract the adverse impacts of childhood maltreatment.

In sum, the factors that cause traumatic event of child maltreatment are mostly extrinsic, either other people acting to harm a person, or a situation that is harmful. By the same token, society more broadly and social groups such as families and schools can provide supportive environments that prevent traumatic events from occurring in the first place.

7. Are there not multiple subtypes and operationalizations of childhood trauma?
Childhood trauma can be measured in multiple ways, and there are multiple types of childhood trauma. This study looked at five measures of childhood trauma – emotional abuse, physical abuse, sexual abuse, physical neglect, and emotional neglect, which were measured either prospectively or retrospectively. The study identified that there was substantial shared genetics between different subtypes, reports, and between different operationalizations of childhood maltreatment.

8. Is it ethically justified to study the genetics of childhood maltreatment?
We are aware that the findings in this study have the potential to be misinterpreted. We want to clarify that the aim of this study is not to identify the genetics of childhood maltreatment per se. Rather, it is to use genetics as a tool to provide better knowledge of the causes and consequences of childhood maltreatment, which may be used to inform practices and policy to decrease or even minimize the risk of childhood maltreatment and mitigate its consequences. In this context, a broader discourse within and outside the scientific community is needed to interpret and communicate the results of this work and related studies appropriately.

While there is already considerable evidence that childhood maltreatment is associated with several adverse mental and physical health conditions, we do not know whether this relationship is causal . For example, these associations can instead arise due to genetic confounding (e.g., parents who have mental health conditions may be more likely to maltreat their children and also pass on genetic variants that increase the likelihood of developing mental health conditions) or due to environmental confounding (e.g., individuals in more deprived backgrounds may be more likely to experience childhood maltreatment, and independently, develop health conditions). It is important to understand the causal relationship for developing appropriate strategies to minimize adverse health risks in individuals who have experienced childhood maltreatment. Genetics provides one tool to investigate this, using methods such as Mendelian Randomization. Other typically used methods to investigate causality such as Randomised Control Trials may be unethical and infeasible for some of the most pertinent question arising in this scenario.

Second, there is well-replicated evidence to suggest that parents who have experienced childhood maltreatment themselves may be more likely to maltreat their children although the risk is certainly not even near to deterministic. It is vital to understand the mechanisms behind this to break the intergenerational cycle of childhood maltreatment. By understanding the mechanisms of gene-environment correlations in childhood maltreatment, we can help to improve parent- and child-targeted interventions to decrease or minimize the risk of childhood maltreatment. Genetics provides a tool to investigate this.
